# CDK6 Is a Potential Prognostic Biomarker in Acute Myeloid Leukemia

**DOI:** 10.3389/fgene.2020.600227

**Published:** 2021-02-01

**Authors:** Wei Liu, Jin-Mou Yi, Yi Liu, Cong Chen, Kai-Xuan Zhang, Cheng Zhou, Hui-En Zhan, Liang Zhao, Stephanie Morales, Xie-Lan Zhao, Hui Zeng

**Affiliations:** ^1^Department of Hematology, Xiangya Hospital, Central South University, Changsha, China; ^2^Department of Hematology, The First Affiliated Hospital of Jinan University, Guangzhou, China; ^3^College of Pharmacy, Western University of Health Sciences, Pomona, CA, United States

**Keywords:** AML, prognosis, *CDK6*, expression, target therapy

## Abstract

Acute myeloid leukemia (AML) is a threatening hematological malignant disease in which new successful approaches in therapy are needed. Cyclin-dependent kinase 6 (*CDK6*), a regulatory enzyme of the cell cycle that plays an important role in leukemogenesis and the maintenance of leukemia stem cells (LSC), has the potential to predict the prognosis of AML. By analyzing public databases, we observed that the messenger RNA (mRNA) levels of *CDK6* were significantly overexpressed in AML cell lines and non-acute promyelocytic leukemia (non-APL) AML patients when compared to healthy donors. Furthermore, *CDK6* expression was significantly reduced in AML patients who achieved complete remission (CR) compared to that at the time of diagnosis in our validated cohort. The expression of *CDK6* was tightly correlated with peripheral blood blasts, French–American–British (FAB) subtypes, CCAAT-enhancer-binding protein α (CEBPA) mutation, and chromosomal abnormalities of t(8;21). However, the clinical significance and effects of *CDK6* expression on the prognosis of non-APL AML patients remain uncertain. We found that *CDK6* expression was inversely correlated with overall survival (OS) among non-APL AML patients using the Kaplan–Meier analysis. *CDK6* was also found to be positively associated with genes identified to contribute to the development of leukemia, including *CCND2*, *DNMT3B*, *SOX4*, and *IKZF2*, as well as being negatively associated with anticancer microRNAs, including miR-187, miR-9, miR-582, miR708, and miR-362. In summary, our study revealed that *CDK6* might be a potential diagnostic and prognostic biomarker in non-APL AML patients.

## Introduction

Acute myeloid leukemia (AML) is a malignant hematological disease with poor prognosis characterized by cytogenetic and molecular heterogeneity. Most APL patients could be cured under all-trans retinoic acid (ATRA) and arsenic trioxide (ATO) chemo-combination regimen. Due to the heterogeneity of leukemia, the general therapeutic strategy of non-APL AML is still a challenge (Walter et al., 2015). Even with transplantations or intensive chemotherapy, some patients still face the risk of relapse. Emerging new therapeutic compounds for AML hold promising prospects, such as targeted inhibition of mutated molecules, tyrosine kinases, and key components of signaling pathways (Abdel-Wahab and Levine, 2013; Gasiorowski et al., 2014).

Compelling research on the biology of AML has provided us with a more comprehensive understanding of cancer-targeted therapies to improve the clinical outcome of AML. Researchers have identified 23 genetic mutations, including *FLT3*, *IDH1/2*, *TET2*, *TP53*, and *DNMT3A*, which have been closely linked to prognosis. Clinically, *FLT3* and *IDH1/2* mutations occur in 20–30% AML patients (Leung et al., 2013; Dohner et al., 2015). With further studies emerging, the presence of *FLT3-ITD* or *IDH1/2* mutation is linked to reduced overall survival rates (Reindl et al., 2006; Eisfeld et al., 2020). In an advanced breakthrough in hematological cancer therapy, the Food and Drug Administration (FDA) has approved the following drugs for AML patients with specific mutations: multitargeted kinase inhibitor midostaurin (Stone et al., 2017), FLT3-ITD, FLT3-TKD, c-Kit activity inhibitor gilteritinib (Perl et al., 2019), IDH1 mutant enzymes inhibitor ivosidenib (DiNardo et al., 2018), and *IDH2* mutant enzymes inhibitor enasidenib (Stein et al., 2017).

Aberrant cell cycle control is also a significant hallmark of cancer cells (Deshpande et al., 2005). During mitotic cell division, cyclin-dependent kinases (CDKs) are critical regulators of G1-S transition. Researchers found that CDK6 and CDK4 have approximately 70% homology in their sequences and closely related biochemical properties (Chan et al., 1995). CDK4 and CDK6 are important regulators for initiation of the cell cycle. Lacking CDK4 and CDK6 resulted in late embryonic lethality due to defects in hematopoiesis (Kozar and Sicinski, 2005). However, evidence shows that the two kinases have different functions beyond cell cycle regulation. *CDK6*, not *CDK4*, is involved in both cell cycle and tumor-promoting progression (Kollmann et al., 2013). During cell cycle progression, changes in oncogene expression are regulated by CDK6 activity (Meyerson and Harlow, 1994). Previous studies have found that *CDK6* is often overexpressed in both leukemia and lymphoma (Chilosi et al., 1998).

Up until now, the complete role of *CDK6* expression in non-APL AML has not been elucidated. In this study, we sought to investigate the pattern of CKD6 expression and to infer the clinical implications of CDK6 in AML patients. To accomplish this, we conducted an analysis on data obtained from Gene Expression Omnibus (GEO) (Barrett et al., 2013), Oncomine (Rhodes et al., 2004), and The Cancer Genome Atlas (TCGA) database (Weinstein et al., 2013) and found that *CDK6* is overexpressed in AML patients. We also validated the expression of *CDK6* in bone marrow (BM) samples of AML patients and healthy donors via quantitative real-time PCR (qRT-PCR). The Kyoto Encyclopedia of Genes and Genomes (KEGG) and Gene Ontology (GO) enrichment analyses were utilized to investigate the underlying molecular mechanisms of *CDK6*. Exploring the clinical features of *CDK6* can help us target AML synergistically with other potential therapies and reduce the frequency of resistance.

## Methods

### Public Database

The gene expression databases ONCOMINE^1^, Gene Expression Profiling Interactive Analysis (GEPIA)^2^, GEO^3^, Xena^4^, Cancer Cell line Encyclopedia (CCLE)^5^, Human Protein Atlas (HPA)^6^, and TCGA^7^ are publicly accessible. The analyses of messenger RNA (mRNA) expressions of CDK6 in human cancer cells were assessed using online tools associated with HPA and CCLE databases. Comparisons between mRNA expressions of CDK6 in subjects with AML cancer and healthy donors were analyzed using ONCOMINE, GEO, and GEPIA. The TCGA database was screened for 173 adult AML patients with CDK6 expression data, complete corresponding clinical features, and non-zero overall survival time. Among them, 135 non-APL AML patients who met the criteria were included in the study; 72 received chemotherapy treatment only, and 63 patients received auto-/allo-HSCT. The major clinical features used in the diagnosis of AML patients are presented in Table 2. In addition, the AML gene expression datasets GSE13159, GSE15061, and GSE34577 from the GEO database were also included. The online web tool GenomicScape^8^ was used to explore the prognostic value of CDK6 expression in CN-AML patients(GSE12417).

### Patients and Ethics

A cohort of 127 newly diagnosed non-APL AML patients, 48 AML-RR patients, 146 AML-CR patients, and 54 healthy donors were enrolled between March 2016 and December 2019 in this study. The French–American–British (FAB) classification of AML patients was according to the 2016 World Health Organization (WHO) criteria. This study was approved by the Xiangya Hospital, Central South University and The First Affiliated Hospital of Jinan University.

### RT-qPCR

Bone marrow mononuclear cells (BMMNCs) were separated using Ficoll-Hypaque (GE Healthcare, United States). Total RNA was extracted from BMMNCs with Trizol reagent (Life Technologies, United States) and reverse transcription to complementary DNA (cDNA) was performed using PrimeScript Kit (TaKaRa, Japan) as described in our previous reports (Liang et al., 2017b). Real-time PCR using ChamQ Universal SYBR Green Master Mix (Vazyme, China) was completed on the ViiATM7 RT-PCR system (Applied Biosystems, United States). The primers used for CDK6 expression were the following: forward, 5′–3′ CTGAATGCTCTTGCTCCTTT; reverse, 5′–3′ AAAGTTTTGGTGGTCCTTGA. Relative CDK6 expression mRNA levels were calculated by 2^–ΔΔCT^ and were normalized to internal control (β-actin).

### Functional Analysis

The differentially expressed genes (DEGs) of RNA and microRNA expression data were analyzed by Rstudio (“edgeR package”). Biological process, molecular function, and cellular component analysis of DEGs were performed using STRING^9^; genomes (KEGG) pathways of CDK6 showed enrichment when analyzed using Gene Set Enrichment Analysis (GSEA)^10^. The microRNAs that could target CDK6 were predicted by online tools miRDB^11^, miRwalk^12^, TargetScan^13^, mirDIP^14^, and DIANA^15^.

### Statistical Analyses

All statistical analyses were completed using SPSS 22.0 and GraphPad Prism 8.0. Either Pearson chi-square analysis or Fisher’s exact test was used for the comparison of categorical variables, whereas Mann–Whitney’s *U*-test was used for the comparison of continuous variables. The prognostic effect of *CDK6* expression was analyzed through Kaplan–Meier analysis using the log-rank test. Univariate and multivariate proportional hazard regression analysis was performed using Cox regression. The *p* < 0.05 (two-tailed) in all statistical analyses was defined as statistically significant.

## Results

### CDK6 Is Overexpressed in AML Patients From the Public Database

**FIGURE 1:**
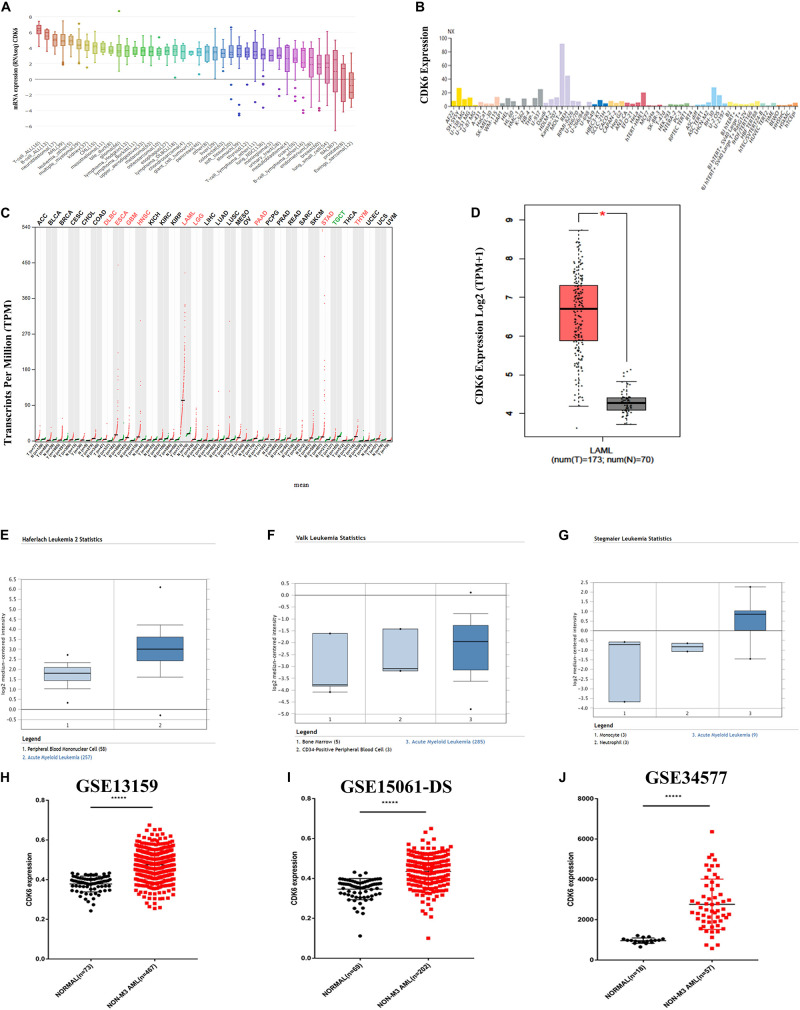
CDK6 overexpression in non-APL AML in the public database. **(A)** The expression of CDK6 in 40 types of human cancer cell line in the Cancer Cell Line Encyclopedia (CCLE) database. **(B)** The expression of CDK6 in human cancer cell lines in Human Protein Atlas (HPA) database. **(C)** The expression of CDK6 in 33 types compared with normal subjects in Gene Expression Profiling Interactive Analysis (GEPIA) database. **(D)** The expression of CDK6 in AML patients (*n* = 173) from TCGA and normal (*n* = 70) from GTX database by using (GEPIA) (*p* < 0.01). **(E–G)** In Haferlach leukemia2, Valk Leukemia, and Stegmaier leukemia statistics, CDK6 was overexpressed in AML patients than normal with a fold change = 2.388, *p* = 2.88E-30, fold change = 1.786, *p* = 0.025 and fold change = 3.179, *p* = 0.011, respectively. **(H)** The expression levels of CDK6 mRNA in non-APL AML patients(*n* = 467) compared with the normal samples (*n* = 73) (mean ± SEM level: 0.4748 ± 0.003325 vs. 0.3783 ± 0.004646, *p* < 0.0001) in GSE13159. **(I)** The expression level of CDK6 mRNA in non-APL AML patients (*n* = 202) compared with the normal samples (*n* = 69) (mean ± SEM level: 613.6 ± 30.5 vs. 253.2 ± 12.21, *n* = 69, *p* < 0.0001) in GSE15061-DS. **(J)** The expression level of CDK6 mRNA in non-APL AML patients (*n* = 57) compared with normal (*n* = 18) (mean ± SEM level: 961.7 ± 31.09 vs. 961.7 ± 31.09, *p* < 0.0001) in GSE34577.

By analyzing 40 different types of the human cancer cell lines in the CCLE database, the expression of *CDK6* was found to be highly expressed in both acute lymphoblastic leukemia (ALL) and AML cell lines (Figure 1A). The HPA public database also presented *CDK6* overexpression in AML cell lines (Figure 1B). We further screened for *CDK6* expression by analyzing the Gene Expression Profiling Interactive Analysis (GEPIA) database and found that the aberrant expression of *CDK6* was observed in AML patients among 33 types of human cancer (Figure 1C). The mRNA expression of *CDK6* in 173 newly diagnosed AML patients was significantly increased compared to the 70 GTEx normal samples (*p* < 0.01) (Figure 1D). In Haferlach leukemia 2, Valk leukemia, and Stegmaier leukemia statistics, the *CDK6* expression was significantly higher in AML patients than in normal samples, using the ONCOMINE database (Figures 1H–J). To further validate the expression of *CDK6* in non-APL AML patients, we analyzed the three independent validation cohorts of the GEO database. Among the GSE13159, GSE34577, and GSE15061-DS GEO sets, the CDK6 levels were significantly upregulated in non-APL AML patients when compared with normal ones (Figures 1E–G).

### CDK6 Is Overexpressed in AML Patients in the Clinical Cohort

**FIGURE 2:**
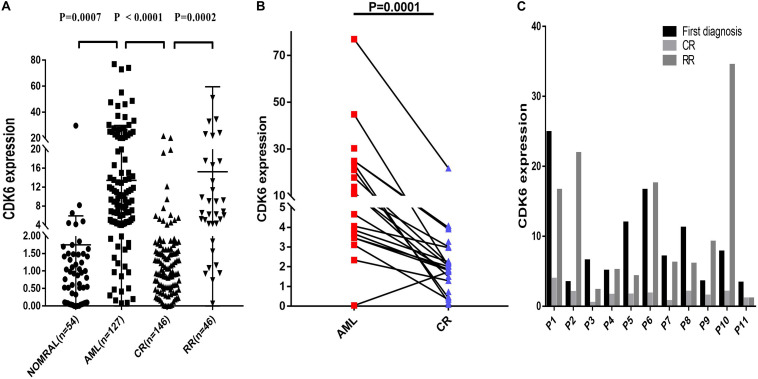
Relative levels of CDK6 expression in the validated non-APL AML patients. **(A)** The CDK6 expression in healthy donors (*n* = 54), de novo non-APL patients (*n* = 127), AML-CR patients (*n* = 146), and AML-RR (*n* = 48) patients from Xiangya Hospital and The First Affiliated Hospital of Jinan University Hospital. The distributions of CDK6 expression were presented with scatter plots. The expression level of CDK6 mRNA in de novo non-APL AML patients (*n* = 127) compared with the normal samples (*n* = 54) (mean ± SEM level: 13.42 ± 1.46 vs. 1.75 ± 0.5703, *p* = 0.007). The expression level of CDK6 mRNA in de novo non-APL AML patients (*n* = 127) compared with AML-CR patients (*n* = 146) (mean ± SEM level: 13.42 ± 1.46 vs. 2.257 ± 0.2894, *p* < 0.0001). The expression level of CDK6 mRNA in AML-RR patients (*n* = 48) compared with AML-CR patients (*n* = 146) (mean ± SEM level: 15.11 ± 6.256 vs. 2.257 ± 0.2894, *p* = 0.0002). **(B)** The CDK6 expression at the time of diagnosis higher than at the time of evaluation for response following standard induction (3 + 7) chemotherapy in patients with AML who achieved CR (*p* = 0.0001) (*n* = paired groups of 29). **(C)** P1–P11 represented 11 paired AML non-APL patients with available follow-up data in first diagnosed time, complete remission (CR) and refractory remission (RR) time. (First diagnosed time vs. CR time: *p* = 0.002; RR vs. CR time: *p* = 0.0086, respectively) (*p*-Values were adjusted by the Holm–Sidak method. HD, healthy donor).

Multiple datasets were utilized as validate sets, and the expression of *CDK6* was elevated in patients with non-APL AML. In order to confirm the public database results, we analyzed another cohort consisting of 54 healthy donors, 127 *de novo* non-APL patients, 146 AML patients who achieved complete remission (CR), and 46 AML refractory remission (RR) patients from Xiangya Hospital and The First Affiliated Hospital of Jinan University. The disease state of AML patients were classified according to the 2016 World Health Organization (WHO) criteria. The relative *CDK6* expression was higher in newly diagnosed AML patients than healthy donors (*p* = 0.0007). The relative *CDK6* expression was noticeably downregulated in AML-CR patients compared to AML-RR patients and AML-*de novo* patients (*p* < 0.0001 and *p* = 0.0002) (Figure 2A). Moreover, we observed that the *CDK6* expression in AML CR patients was significantly reduced compared to the original measurement when first diagnosed (*p* = 0.0001) (Figure 2B). To monitor the dynamic change of CDK6 expression in non-APL AML patients in different clinical stages, we tested CDK6 expression in 11 paired patients with available follow-up data at first diagnosis, complete remission (CR), and refractory remission (RR) time (*de novo* vs. CR: *p* = 0.002; RR vs. CR: *p* = 0.0086, respectively) (Figure 2C).

### The Expression of CDK6 Is Associated With the Prognosis of CN-AML Patients

**FIGURE 3:**
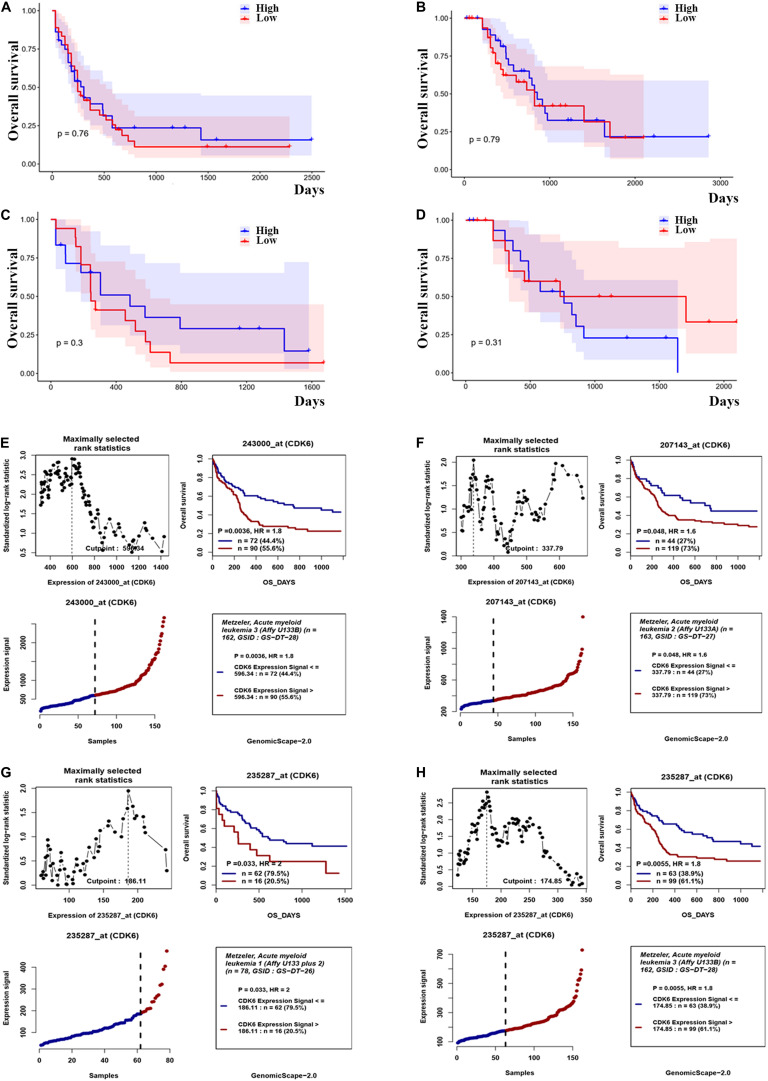
The effect of CDK6 expression on overall survival in non-APL AML patients. **(A)** Kaplan–Meier analysis of OS in chemotherapy only group in non-APL AML patients [CDK6high (*n* = 36) vs. CDK6low (*n* = 36): *p* = 0.76] (cut point: median CDK6 expression level). **(B)** Kaplan–Meier analysis of OS in auto-/allo-HSCT group in non-APL AML patients [CDK6high (*n* = 31) vs. CDK6low (*n* = 32): *p* = 0.79] (cut point: median CDK6 expression level). **(C)** Kaplan–Meier analysis of OS in chemotherapy only group in cytogenetically normal AML patients [CDK6high (*n* = 17) vs. CDK6low (*n* = 18): *p* = 0.3] (cut point: median CDK6 expression level). **(D)** Kaplan–Meier analysis of OS in auto-/allo-HSCT group in cytogenetically normal AML patients [CDK6high (*n* = 17) vs. CDK6low (*n* = 18): *p* = 0.3] (cut point: median CDK6 expression level). **(E)** The survival analysis of probe 243000_at of 162 cytogenetically normal AML (CN-AML) patients using the online web tool GenomicScape (cut point: 596.34, *p* = 0.0036, HR = 1.8); **(F)** the survival analysis of probe 207143_at of 162 cytogenetically normal AML (CN-AML) patient (cut point: 337.79, *p* = 0.048, HR = 1.6). **(G)** The survival analysis of probe 235287_at of 78 cytogenetically normal AML (CN-AML) patients using the online web tool Genomicscape (cut off: 596.34, *p* = 0.033, HR = 2). **(H)** The survival analysis of probe 235287_at of 162 cytogenetically normal AML (CN-AML) patients using the online web tool Genomicscape (cut point: 596.34, *p* = 0.0055, HR = 1.8).

Based on the above observations of increased CDK6 in AML patients, we hypothesized that there was a relationship between abnormal *CDK6* expression and the therapeutic outcomes of AML patients. We analyzed the relative expression of CDK6 to AML patient survival in previously published datasets. Among the TCGA AML patients, 72 non-APL patients received only standard induction chemotherapy, whereas the 63 non-APL patients received auto-/allo-HSCT after induction chemotherapy. In both groups, *CDK6* expression did not affect overall prognosis based on Kaplan–Meier analysis. The OS of *CDK6*^high^ patients showed no significant difference compared with the OS of *CDK6*^low^ patients in auto-/allo-HSCT and chemotherapy only groups (OS median: 822 vs. 854 days, *p* = 0.79; 245 vs. 304 days, *p* = 0.76, respectively). In cytogenetically normal AML (CN-AML) patients, there was also no difference between *CDK6*^high^ and *CDK6*^low^ group, no matter with auto-/allo-HSCT or chemotherapy only (chemotherapy only group: OS median, 245 vs. 485 days, *p* = 0.3; auto-/allo-HSCT group: OS median, 731 vs. 761 days, *p* = 0.31) (Figures 3A–D). Cox regression analysis also identified that *CDK6* expression could not be an independent factor of OS in whole-TCGA-AML patients (Table 1). To further explore the impact of high *CDK6* in CN-AML, we analyzed the overall survival of three CN-AML patient cohorts from the GEO database (GSE12417) by using an online tool GenomicScape. Each set’s patients were divided into two groups: those with above-cutoff *CDK6* expression and those with below-cutoff *CDK6* expression. Above-cutoff *CDK6* expression was associated with negative OS in CN-AML patients (Figures 3E–H; Supplementary Figure 1).

**TABLE 1:** Univariate and multivariate analysis of variables of OS in non-M3 AML patients.

**Variables**				
	**Univariate analysis**		**Multivariate analysis**	
	**HR(95%CI)**	**P**	**HR(95%CI)**	***P***
CDK6 expression	1.000(1.000-1.000)	0.047	1.000(1.000-1.000)	0.069
Age	1.033(1.019-1.048)	0	1.018(1.000-1.036)	0.045
BM_Blast_Percentage	1.004(0.994-1.014)	0.46	1.013(1.001-1.026)	0.039
WBC	1.003(0.999-1.007)	0.191	1.002(0.997-1.008)	0.411
PB Blast_Percentage	0.998(0.992-1.005)	0.617	1.005(0.996-1.014)	0.265
Risk cytogenetic-poor	1.859(1.229-2.813)	0.003	2.444(1.417-4.215)	0.001
Treatment transplant	0.414(0.281-0.611)	0	0.367(0.223-0.604)	0
WT1	0.871(0.404-1.875)	0.723	1.14(0.490-2.650)	0.761
TP53	3.167(1.817-5.520)	0	2.761(1.314-5.798)	0.007
FLT3	1.234(0.811-1.876)	0.326	1.258(0.727-2.176)	0.411
TET2	0.896(0.468-1.718)	0.742	0.691(0.341-1.400)	0.305
RUNX1	1.472(0.821-2.638)	0.194	3.290(1.619-6.686)	0.001
IDH2	0.929(0.509-1.697)	0.811	0.521(0.261-1.040)	0.065
IDH1	0.629(0.318-1.246)	0.184	0.497(0.215-1.144)	0.1
NRAS	0.810(0.376-1.745)	0.591	0.632(0.274-1.456)	0.281
NPM1	1.014(0.674-1.526)	0.946	0.872(0.471-1.616)	0.664
KRAS	1.528(0.670-3.482)	0.314	1.876(0.710-4.955)	0.205
DNMT3A	1.393(0.923-2.102)	0.114	1.874(1.145-3.067)	0.012
CEBPA	0.833(0.421-1.648)	0.599	2.504(1.134-5.528)	0.023

### Association Between CDK6 Expression and Clinical Characteristics

To explore the clinical feature of *CDK6* expression in non-APL AML patients, we compared the clinical characteristics of non-APL AML patients between *CDK6*^high^ and *CDK6*^low^ groups (divided by the median level of *CDK6* expression) (Table 2). CDK6^high^ patients had higher peripheral blood blasts compared with *CDK6*^*low*^ group (*p* < 0.0001). However, no significant differences were found in gender, white blood cell (WBC), percentage of BM blasts, treatment, and risk cytogenetics between the two groups. In addition, significant differences were observed in the distributions of FAB subtypes. The *CDK6*^high^ group frequently occurred in FAB-M0/M1/M2 (*p* < 0.0001, *p* = 0.005, and *p* = 0.001, respectively) and less frequently occurred in FAB M4/M5 (*p* < 0.0001 and *p* < 0.0001). Among cytogenetics and gene mutations, high *CDK6* expression was associated with CEBPA mutations and chromosomal abnormalities of *t*(8;21) (*p* = 0.01 and *p* = 0.039), and low *CDK6*expression was associated with inv(16) (*p* = 0.021).

**TABLE 2:** Correlation of CDK6 expression of clinical characteristics in non-APL AML patients.

**Characteristics**	**Low (*n* = 78)**	**High (*n* = 79)**	***P*-value**
Male/Female	42/36	43/36	0.941
Median age, years (range)	62 (31-88)	56 (18-82)	0.006
Median WBC (10^9^/l, range)	25.9 (1.2-137.2)	14.3 (0.6-297)	0.281
Median BM blasts (range)	71 (30-98)	72 (32-100)	0.209
Median PB blasts (range)	17 (0-90)	54 (0-98)	0.000
**FAB**			
M0	1	15	0.000
M1	14	30	0.005
M2	12	26	0.01
M4	32	2	0.000
M5	16	2	0.000
M6	2	0	0.471
M7	0	3	0.248
No data	1	1	
**Cytogenetics**			
normal	40	37	0.377
inv(16)	9	1	0.021
-7/7q-	1	4	0.371
t(8;21)	0	6	0.039
del(5)	0	1	1
11q23	4	0	0.125
+8	3	5	0.731
complex	10	14	0.394
Other	9	8	0.776
No data	1	2	
**Risk cytogenetic**			
Good	9	8	0.776
Intermediate	51	49	0.662
Poor	18	22	0.493
**Mutation**			
FLT3	23	21	0.685
NPM1	29	19	0.074
DNMT3A	22	21	0.820
IDH2	7	10	0.458
TET2	6	9	0.430
RUNX1	7	9	0.617
TP53	6	9	0.430
NRAS	6	5	0.738
CEBPA	2	11	0.01
WT1	3	7	0.337
KRAS	5	2	0.429
**Treatment**			
CR	24	32	0.203

### Molecular Feature of CDK6 in AML

**FIGURE 4:**
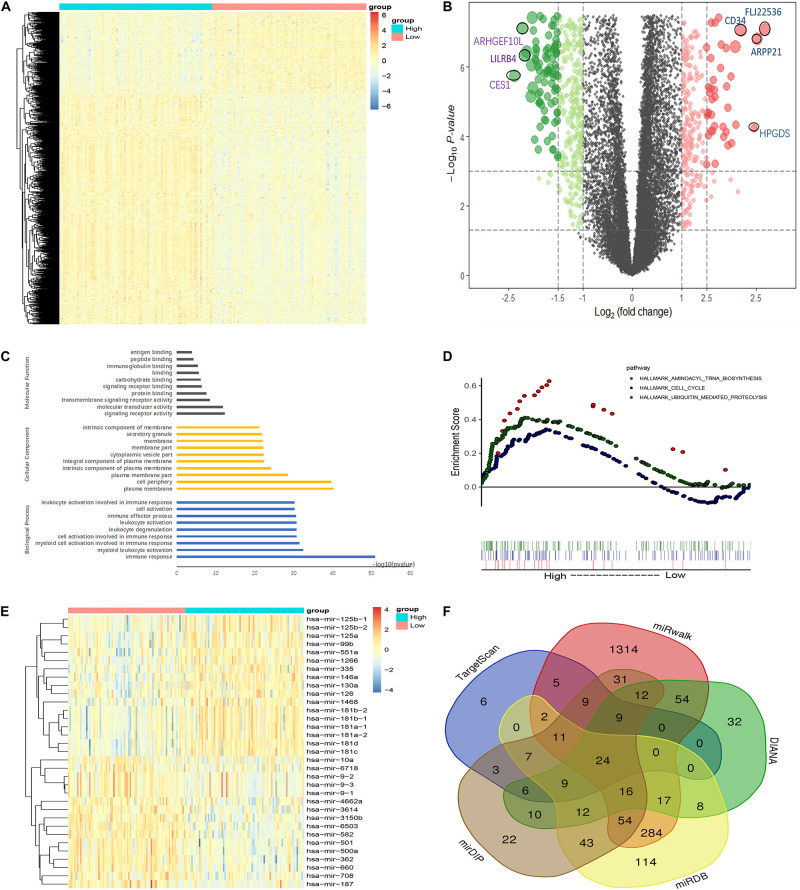
Molecular signatures associated with CDK6 in non-APL AML. **(A)** Heatmap of differentially expressed genes (DEGs) between CDK6^*high*^ and CDK6^*low*^ non-APL AML patients. **(B)** Volcano plot of DEGs between CDK6^*high*^ and CDK6^*low*^ non-APL AML patients. **(C)** Biological process, molecular functions, and cellular component analysis of DEGs using the online website of Search Tool for the Retrieval of Interacting Genes/Proteins. **(D)** KEGG pathway analysis between CDK6^*high*^ and CDK6^*low*^ group by using the online tool Gene Set Enrichment Analysis (GSEA); the red dot represents aminoacyl tRNA biosynthesis pathway (NES = 1.78, *p* = 0.003), the blue dot represents the cell cycle pathway (NES = 1.35, *p* = 0.021), the green dot represents ubiquitin-mediated proteolysis pathway (NES = 1.64, *p* < 0.0001). **(E)** Heatmap of DEGs miRNAs CDK6^*high*^ and CDK6^*low*^ AML patients. **(F)** Venn results of microRNAs that could target CDK6 precited by online tools miRDB, miRwalk, TargetScan, mirDIP, and DIANA.

To investigate the biological function of *CDK6*, we compared the transcriptome between *CDK6*^high^ and *CDK6*^low^ group divided by median *CDK6* expression. The comparison resulted in 1,280 DEGs [false discovery rate (FDR) < 0.05, Log2FC > 1, Figures 4A,B and Supplementary Table 1], in which 331 positively correlated with *CDK6* expression and 949 negatively correlated with *CDK6* expression. *CCND2, DNMT3B, SOX4*, and *IKZF2*, all previously reported to associate with leukemia development, were found within the positively correlated genes (Hanamura et al., 2006; Zhang et al., 2013; Niederwieser et al., 2015; Park et al., 2019). To further explore the role of *CDK6* in AML, Gene Ontology analysis showed that DEGs were involved in signaling receptor activity, cell activation, immune response, and molecular transducer activity (Figure 4C). GSEA between *CDK6*^high^ and *CDK6*^low^ group identified more noticeable changes in the aminoacyl transfer RNA (tRNA) biosynthesis pathway, cell cycle pathway, and ubiquitin-mediated proteolysis pathway (Figure 4D). MicroRNAs are known as regulators of the oncogenesis of AML. The comparison between two groups also yielded 33 correlated microRNAs (Supplementary Table 2); among them, negatively correlated microRNAs, including miR-187, miR-9, miR-582, miR708, and miR-362, have been reported to have anticancer effects in previous studies (Liang et al., 2017a; Shi and Zhang, 2017; Li et al., 2019; Schneider et al., 2019; Xu et al., 2019) and were predicted to be able to directly target *CDK6* (Figures 4E,F).

## Discussion

Abnormal regulation of *CDK6* is a hallmark of cancer. Increased *CDK6* expression has been found in several human cancer. In our study, the significance of *CDK6* expression in prognosis and the clinical relevance were investigated in non-APL AML. We determined that the expression of *CDK6* was positively upregulated in *de novo* AML patients compared to healthy donors found in the public databases. The results of our study cohort remained consistent with those of previous studies, as well.

*CDK6* has critical roles in multiple aspects of cell biology. It has been shown that *CDK6* is involved in the biological function of leukemogenesis such as cell proliferation, cell apoptosis, and cell cycle regulation. *CDK6* was not only known to be a cell-cycle kinase but also has a unique role of directly regulating transcription factors, which include *AP-1* and vascular endothelial growth factor (*VEGF-A*), promoting tumor angiogenesis (Otto and Sicinski, 2013). *CDK6* also plays a crucial role in malignant hematopoietic tumors. It is not only required for maintenance of NUP98-fusion AML but also could act an important role in MLL (KMT2A) fusions-mediated myeloid leukemogenesis (Schmoellerl et al., 2020). Some AML patients with FLT3-ITD mutation failed to induce a persistent response via FLT3 inhibitor. However, *CDK6* showed a regulatory role in cell survival and cell apoptosis of FLT3 + AML cells (Uras et al., 2016). Reduced CDK6 kinase activity represents an encouraging target for anticancer drugs. Palbociclib, a *CDK4/CDK6* inhibitor, demonstrated a potent effect in some cancer drugs. In patients with estrogen-receptor (ER)-positive breast cancer, treatment using palbociclib with fulvestrant resulted in prolonged progression-free survival (Turner et al., 2018). Based on the result of a phase Ib clinical trial, palbociclib has a positive effect in refractory/relapsed MLL (KMT2A)-rearranged leukemia patients with no incidence of limiting toxicities (NCT02310243) (Fröhling et al., 2016). A clinical trial is ongoing to estimate the efficacy and safety of palbociclib alone or in combination with sorafenib, decitabine, and dexamethasone in AML patients with relapsed and refractory (RR) leukemias (NCT03132454) (Kadia et al., 2018). Targeting *CDK6* can act as a novel approach to increase chemotherapy response in AML patients.

MicroRNAs were recognized to act as oncomiRs and disease biomarkers in hematological malignancy (Wallace and O’Connell, 2017). *CDK6* is targeted by many miRNAs, such as miR-29b, miR-218, miR-582, and miR-187. A recent report found miR-29b, which upregulates *CDK6*, to be a tumor suppressor via targeting multiple important oncogenic pathways (Huang et al., 2013). The expression of *CDK6* was found to be negatively correlated with miR-187and miR-582.

The correlation between the expression of *CDK6* and clinical features of AML patients has not yet been reported. The expression of *CDK6* and correlation with AML clinical feature was also not examined in non-APL AML patients. In our study, we aimed to identify the clinical significance of *CDK6* in non-APL AML. A significant adverse effect of high *CDK6* expression on OS was observed among CN-AML patients, which indicated that *CDK6* could be a potential prognostic and therapeutic value in AML.

## Data Availability Statement

Publicly available datasets were analyzed in this study. This data can be found here: ONCOMINE (https://www.oncomine.org/), GEPIA(http://gepia.cancer-pku.cn/), GEO (https://www.ncbi.nlm.nih.gov/geo/), Xena (https://xenabrowser.net/), CCLE (https://portals.broadinstitute. org/ccle), HPA (https://www.proteinatlas.org/), and TCGA (https://www.cancer.gov/tcga).

## Ethics Statement

The studies involving human participants were reviewed and approved by the Xiangya Hospital, Central South University and Huaqiao Hospital, The First Affiliated Hospital of Jinan University. The patients/participants provided their written informed consent to participate in this study.

## Author Contributions

HZ designed the study and approved the final manuscript. WL performed the experiments and analyzed the data. J-MY, YL, CC, K-XZ, and CZ collected the clinical sample and data. H-EZ, LZ, and X-LZ performed the rest of necessary experiments. WL, SM, and HZ wrote and edited the manuscript. All authors contributed to the article and approved the submitted version.

## Conflict of Interest

The authors declare that the research was conducted in the absence of any commercial or financial relationships that could be construed as a potential conflict of interest.

## Footnotes

1 https://www.oncomine.org/

2 http://gepia.cancer-pku.cn/

3 https://www.ncbi.nlm.nih.gov/geo/

4 https://xenabrowser.net/

5 https://portals.broadinstitute.org/ccle

6 https://www.proteinatlas.org/

7 https://www.cancer.gov/tcga

8 http://genomicscape.com/microarray/survival.php

9 https://string-db.org/

10 https://www.gsea-msigdb.org/gsea/

11 http://mirdb.org/

12 http://zmf.umm.uni-heidelberg.de/apps/zmf/mirwalk2/

13 http://www.targetscan.org

14 http://ophid.utoronto.ca/mirDIP/

15 http://diana.imis.athena-innovation.gr/DianaTools/index.php

## Abbreviations

AML: acute myeloid leukemia
GO: Gene Ontology
KEGG: Kyoto Encyclopedia of Genes and Genomes
GEPIA: Gene Expression Profiling Interactive Analysis
TCGA: The Cancer Genome Atlas
HSCT: hematopoietic stem cell transplantation
OS: overall survival
CDKs: cyclin-dependent kinases
CR: complete remission
RR: refractory remission
WHO: World Health Organization
CCLE: Cancer Cell line Encyclopedia
HPA: Human Protein Atlas
APL: acute promyelocytic leukemia
ATRA: all-trans retinoic acid
ATO: arsenic trioxide
CEBPA: CCAAT-enhancer-binding protein α.
