# Supplementary material for: CDK6 Is a Potential Prognostic Biomarker in Acute Myeloid Leukemia
Source: Front Genet. 2021 Feb 1;11:600227. doi: 10.3389/fgene.2020.600227 (PMC7882723; doi:10.3389/fgene.2020.600227)
Supplement: Supplementary file 1 [file Data_Sheet_1.docx]

**
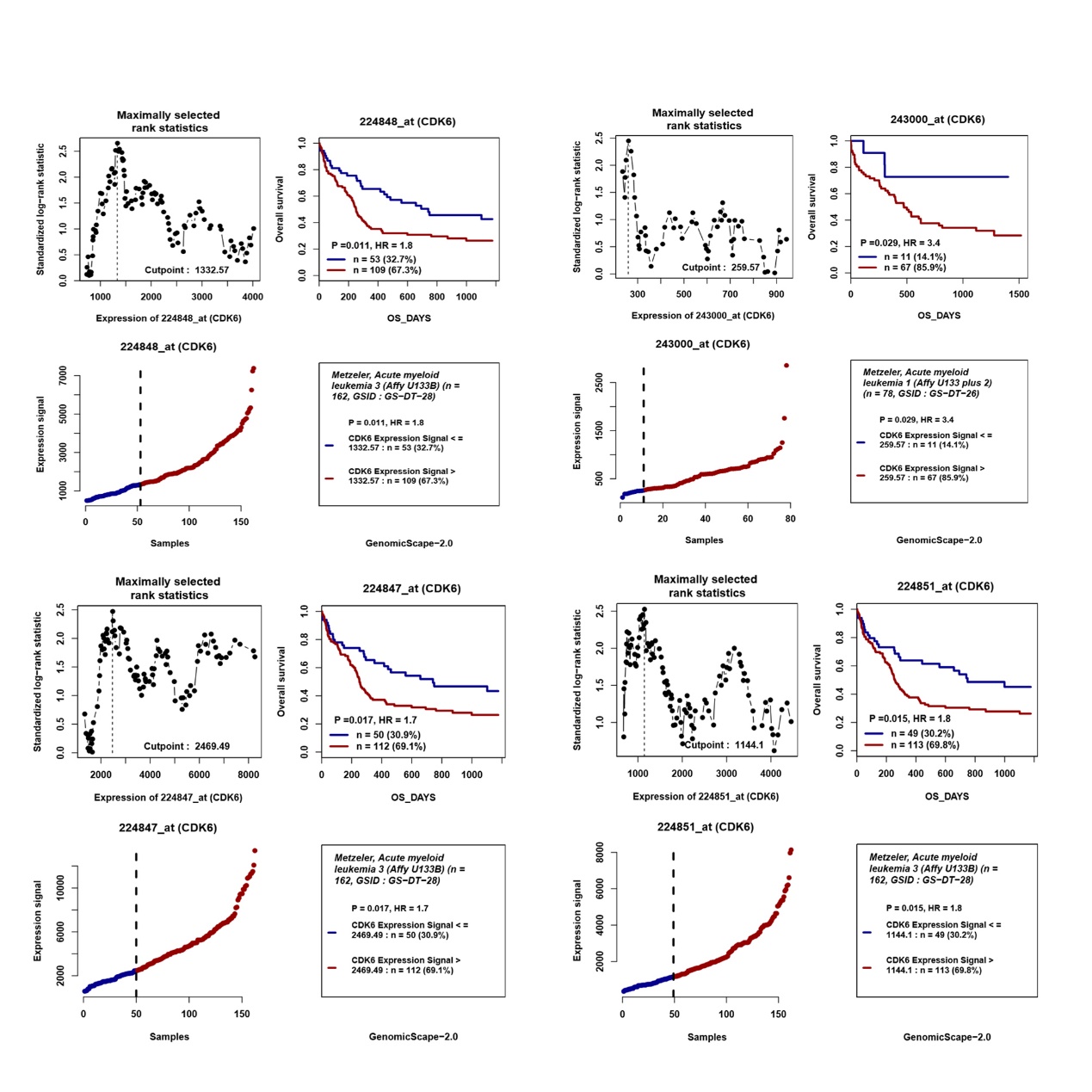
**

Supplementary Figure 1 The effect of CDK6 expression on OS in CN-AML patients, which obtained from GSE12417 database by analyzing online web-tool GenomicScape. (A) probe224848_at in 162 CN-AML patients (cut off:1332.57,p=0.011,HR=1.8);(B) probe243000_at in 87 CN-AML patients (cut off:259.57, p=0.029,HR=3.4). (C) probe224847_at in 162 CN-AML patients (cut off:2469.49,p=0.017,HR=1.7).(D) probe224851_at in 162 CN-AML patients (cut off:1144.1, p=0.015, HR=1.8).
