# Supplementary material for: CDK6 Is a Potential Prognostic Biomarker in Acute Myeloid Leukemia
Source: Front Genet. 2021 Feb 1;11:600227. doi: 10.3389/fgene.2020.600227 (PMC7882723; doi:10.3389/fgene.2020.600227)
Supplement: Supplementary file 2 [file Table_1.docx]

Supplementary Table1: DEGS of mRNA between C*DK6*^high^  and  *CDK6*^low^ in non-APL AML patients

|  | logFC | AveExpr | t | P.Value | adj.P.Val | B |
| --- | --- | --- | --- | --- | --- | --- |
| CES1 | -4.6311113 | 4.390359806 | -9.8378391 | 4.30E-18 | 5.05E-16 | 30.55985 |
| ARHGEF10L | -4.4135901 | 6.187030161 | -11.619804 | 6.40E-23 | 3.99E-20 | 41.4728 |
| LILRB4 | -4.2815265 | 7.281061723 | -10.350108 | 1.81E-19 | 3.34E-17 | 33.6681 |
| CD14 | -4.2292829 | 8.389166598 | -11.627964 | 6.08E-23 | 3.99E-20 | 41.52324 |
| CD300E | -4.1916312 | 5.210344624 | -10.821283 | 9.61E-21 | 2.55E-18 | 36.55137 |
| HK3 | -4.1447174 | 8.531673695 | -12.455135 | 3.31E-25 | 6.70E-22 | 46.64281 |
| S100A9 | -4.115151 | 11.14241434 | -10.435867 | 1.06E-19 | 2.18E-17 | 34.19136 |
| MEFV | -4.038334 | 7.267097284 | -12.434639 | 3.76E-25 | 6.78E-22 | 46.5159 |
| MARCO | -4.0063844 | 3.743369906 | -11.008194 | 2.98E-21 | 9.68E-19 | 37.70011 |
| S100A12 | -3.9678542 | 7.073959356 | -9.1057308 | 3.72E-16 | 2.43E-14 | 26.18353 |
| PLBD1 | -3.9641249 | 7.627783943 | -9.3947001 | 6.46E-17 | 5.19E-15 | 27.90015 |
| S100A8 | -3.9419053 | 10.64245991 | -9.7688751 | 6.56E-18 | 6.96E-16 | 30.14398 |
| LILRA5 | -3.779989 | 6.61847811 | -10.114475 | 7.80E-19 | 1.21E-16 | 32.23444 |
| PRLR | -3.7243877 | 5.457152582 | -8.6437158 | 5.90E-15 | 2.82E-13 | 23.47349 |
| CDA | -3.7129286 | 6.596646984 | -11.233439 | 7.26E-22 | 3.10E-19 | 39.08739 |
| MAFB | -3.6687929 | 8.156088211 | -11.575946 | 8.43E-23 | 5.07E-20 | 41.20173 |
| VCAN | -3.6677012 | 12.12455206 | -9.1546718 | 2.77E-16 | 1.86E-14 | 26.47317 |
| MYOF | -3.602245 | 7.728223746 | -9.5608861 | 2.35E-17 | 2.15E-15 | 28.894 |
| ASGR2 | -3.5849127 | 4.892827432 | -10.731254 | 1.69E-20 | 4.14E-18 | 35.99898 |
| MPEG1 | -3.5812249 | 11.3209163 | -10.951997 | 4.24E-21 | 1.25E-18 | 37.35447 |
| TLR8 | -3.5689234 | 8.140225054 | -10.024519 | 1.36E-18 | 1.84E-16 | 31.68883 |
| CD1D | -3.5623007 | 7.36395443 | -11.209184 | 8.45E-22 | 3.52E-19 | 38.93787 |
| SERPINA1 | -3.526456 | 10.12850374 | -8.9595908 | 8.95E-16 | 5.46E-14 | 25.32144 |
| FCN1 | -3.4923708 | 9.516900447 | -9.4147448 | 5.72E-17 | 4.71E-15 | 28.01978 |
| CD163 | -3.47325 | 8.194969711 | -8.2116777 | 7.49E-14 | 2.64E-12 | 20.98421 |
| C5AR1 | -3.4399737 | 8.557845102 | -10.586699 | 4.15E-20 | 9.23E-18 | 35.1134 |
| SLC11A1 | -3.4139034 | 8.883252036 | -11.755741 | 2.72E-23 | 2.32E-20 | 42.31335 |
| C17orf55 | -3.4067144 | 4.354946567 | -10.273472 | 2.91E-19 | 4.73E-17 | 33.20115 |
| SIGLEC9 | -3.3798267 | 6.876251475 | -11.082789 | 1.87E-21 | 6.89E-19 | 38.15921 |
| IL1RN | -3.3628016 | 7.186796099 | -11.386815 | 2.77E-22 | 1.28E-19 | 40.03357 |
| LRP1 | -3.3611404 | 10.81590572 | -10.03175 | 1.30E-18 | 1.77E-16 | 31.73265 |
| LILRA6 | -3.3586648 | 7.973622784 | -10.680104 | 2.32E-20 | 5.46E-18 | 35.68542 |
| NCF1 | -3.3493353 | 8.773335527 | -12.660048 | 9.08E-26 | 2.95E-22 | 47.91139 |
| NCF1C | -3.3480468 | 7.12710952 | -12.001448 | 5.78E-24 | 5.51E-21 | 43.83376 |
| SERPINB2 | -3.3376077 | 6.411661988 | -8.5646461 | 9.42E-15 | 4.28E-13 | 23.01445 |
| NLRP12 | -3.3348988 | 7.532283044 | -11.01684 | 2.82E-21 | 9.48E-19 | 37.7533 |
| MYCL1 | -3.3224924 | 5.246981924 | -9.5044213 | 3.31E-17 | 2.87E-15 | 28.55581 |
| KLF4 | -3.2881414 | 9.320499154 | -12.536831 | 1.97E-25 | 4.58E-22 | 47.14863 |
| FPR1 | -3.2809871 | 7.59266088 | -8.865904 | 1.57E-15 | 8.87E-14 | 24.77108 |
| MYO7A | -3.2569545 | 5.406649654 | -9.828681 | 4.54E-18 | 5.26E-16 | 30.50459 |
| LGALS2 | -3.2485292 | 4.217630483 | -7.717669 | 1.29E-12 | 3.32E-11 | 18.20059 |
| AQP9 | -3.2323865 | 6.752726459 | -8.3800024 | 2.80E-14 | 1.13E-12 | 21.94842 |
| CLEC10A | -3.224209 | 4.469778541 | -7.4351175 | 6.34E-12 | 1.39E-10 | 16.64277 |
| CYP27A1 | -3.1942265 | 4.948422461 | -8.3815477 | 2.77E-14 | 1.12E-12 | 21.95731 |
| MTMR11 | -3.1903223 | 5.665572741 | -9.8838174 | 3.24E-18 | 4.01E-16 | 30.83748 |
| SLC15A3 | -3.1871059 | 7.00872141 | -12.734174 | 5.69E-26 | 2.95E-22 | 48.37017 |
| FCAR | -3.1765391 | 6.855576319 | -8.874346 | 1.49E-15 | 8.59E-14 | 24.8206 |
| EPB41L3 | -3.1749764 | 5.389599752 | -8.1978082 | 8.12E-14 | 2.82E-12 | 20.9051 |
| VNN3 | -3.165652 | 4.499953655 | -10.287589 | 2.67E-19 | 4.42E-17 | 33.28712 |
| NFAM1 | -3.1564242 | 9.649722848 | -11.542687 | 1.04E-22 | 5.70E-20 | 40.99621 |
| LILRA3 | -3.1292604 | 5.08479087 | -7.6518249 | 1.87E-12 | 4.63E-11 | 17.8352 |
| LYZ | -3.1207154 | 15.19933785 | -9.6751805 | 1.17E-17 | 1.18E-15 | 29.58008 |
| SLC7A7 | -3.1124984 | 8.512045498 | -10.936777 | 4.66E-21 | 1.33E-18 | 37.2609 |
| FBP1 | -3.0938168 | 6.909318764 | -10.329511 | 2.06E-19 | 3.66E-17 | 33.54254 |
| RBM47 | -3.0910376 | 8.698393714 | -10.973123 | 3.71E-21 | 1.12E-18 | 37.48438 |
| IFI30 | -3.0871805 | 10.35874261 | -12.020582 | 5.12E-24 | 5.21E-21 | 43.9522 |
| NAPSB | -3.061132 | 7.204053706 | -9.2185159 | 1.88E-16 | 1.32E-14 | 26.8517 |
| SIRPD | -3.0296253 | 3.542954055 | -11.715667 | 3.50E-23 | 2.84E-20 | 42.06551 |
| APOBEC3A | -3.0105293 | 6.346094334 | -9.3725163 | 7.39E-17 | 5.82E-15 | 27.76784 |
| SLC6A12 | -2.9790068 | 3.484050273 | -10.063846 | 1.07E-18 | 1.53E-16 | 31.92723 |
| MS4A4A | -2.977737 | 6.527353426 | -9.5776138 | 2.12E-17 | 1.99E-15 | 28.99428 |
| CYBB | -2.9762755 | 11.52280104 | -9.8278594 | 4.57E-18 | 5.26E-16 | 30.49963 |
| TLR5 | -2.971221 | 6.816578639 | -11.431786 | 2.09E-22 | 1.06E-19 | 40.31121 |
| SIGLEC7 | -2.9682744 | 6.0153163 | -10.373369 | 1.57E-19 | 3.03E-17 | 33.80995 |
| HSPA7 | -2.9606608 | 6.029258233 | -9.5903702 | 1.96E-17 | 1.86E-15 | 29.07079 |
| LRRC25 | -2.955699 | 8.727433479 | -12.775544 | 4.38E-26 | 2.95E-22 | 48.62618 |
| SECTM1 | -2.9542122 | 6.805033776 | -9.8491943 | 4.01E-18 | 4.81E-16 | 30.62839 |
| UNC5A | -2.9523417 | 3.768704142 | -10.992205 | 3.30E-21 | 1.04E-18 | 37.60175 |
| LILRB2 | -2.9301735 | 9.160893541 | -10.322021 | 2.16E-19 | 3.76E-17 | 33.49689 |
| C2 | -2.9172891 | 5.859181158 | -6.83902 | 1.67E-10 | 2.60E-09 | 13.45015 |
| MS4A6A | -2.9115211 | 9.921781965 | -9.5901417 | 1.96E-17 | 1.86E-15 | 29.06942 |
| NCF1B | -2.9015667 | 7.853826196 | -12.578741 | 1.52E-25 | 4.10E-22 | 47.40809 |
| KCNC3 | -2.8969553 | 5.326925255 | -9.0527703 | 5.11E-16 | 3.23E-14 | 25.87062 |
| KIAA1598 | -2.8920102 | 6.79716468 | -8.3102619 | 4.21E-14 | 1.60E-12 | 21.54802 |
| TMEM105 | -2.8727337 | 3.764900385 | -7.8049222 | 7.84E-13 | 2.15E-11 | 18.68691 |
| IL31RA | -2.8679245 | 2.901838199 | -8.8324104 | 1.92E-15 | 1.06E-13 | 24.57478 |
| CHST15 | -2.8606446 | 8.415963829 | -10.497005 | 7.26E-20 | 1.55E-17 | 34.56484 |
| SIGLEC1 | -2.860553 | 5.976483807 | -6.6808593 | 3.90E-10 | 5.52E-09 | 12.62661 |
| FGD2 | -2.8419194 | 9.228326236 | -12.020144 | 5.14E-24 | 5.21E-21 | 43.94949 |
| LGALS3 | -2.8251047 | 7.74593645 | -11.33667 | 3.80E-22 | 1.71E-19 | 39.7241 |
| LRRK2 | -2.8061468 | 8.358170295 | -8.6858828 | 4.59E-15 | 2.24E-13 | 23.7189 |
| TLR7 | -2.7969699 | 7.07557178 | -8.748034 | 3.17E-15 | 1.62E-13 | 24.08134 |
| LILRB3 | -2.7945861 | 8.346144118 | -10.499906 | 7.13E-20 | 1.54E-17 | 34.58257 |
| CX3CR1 | -2.7887035 | 9.789475875 | -9.770059 | 6.51E-18 | 6.95E-16 | 30.15112 |
| MSR1 | -2.7768065 | 4.906454503 | -6.8209798 | 1.84E-10 | 2.84E-09 | 13.35569 |
| CLEC7A | -2.7728498 | 8.125815392 | -9.2959303 | 1.18E-16 | 8.80E-15 | 27.3117 |
| FGR | -2.7709168 | 10.83785209 | -12.80169 | 3.72E-26 | 2.95E-22 | 48.78795 |
| NOD2 | -2.7659471 | 7.939646847 | -10.987064 | 3.40E-21 | 1.04E-18 | 37.57013 |
| CCR1 | -2.7630514 | 8.574961884 | -10.920375 | 5.17E-21 | 1.42E-18 | 37.16008 |
| TREM1 | -2.7618032 | 6.784299532 | -9.700043 | 1.00E-17 | 1.03E-15 | 29.72959 |
| PADI2 | -2.7613966 | 7.47096754 | -9.1231681 | 3.35E-16 | 2.20E-14 | 26.28667 |
| CACNA2D3 | -2.7543642 | 4.632036622 | -7.1071366 | 3.90E-11 | 7.10E-10 | 14.86945 |
| SORT1 | -2.7495893 | 9.243654826 | -8.2012836 | 7.96E-14 | 2.78E-12 | 20.92492 |
| ADAMTS2 | -2.7461148 | 4.467177449 | -5.8414716 | 2.89E-08 | 2.54E-07 | 8.445629 |
| CRISPLD2 | -2.7438654 | 7.111755044 | -9.0709775 | 4.58E-16 | 2.94E-14 | 25.97813 |
| CD300LB | -2.7402518 | 7.371909197 | -10.24133 | 3.56E-19 | 5.61E-17 | 33.00549 |
| DTNA | -2.7311827 | 4.313773364 | -6.2001447 | 4.77E-09 | 5.11E-08 | 10.19106 |
| S1PR3 | -2.7182246 | 6.183658486 | -7.9598431 | 3.22E-13 | 9.66E-12 | 19.55617 |
| SPOCK1 | -2.7086346 | 3.89226665 | -9.231534 | 1.74E-16 | 1.23E-14 | 26.92898 |
| KCTD12 | -2.7063111 | 10.21818129 | -9.5072232 | 3.25E-17 | 2.84E-15 | 28.57258 |
| FPR2 | -2.7030179 | 6.079217603 | -7.6339509 | 2.07E-12 | 5.05E-11 | 17.73626 |
| KCNQ1 | -2.6991201 | 7.064452545 | -11.146967 | 1.25E-21 | 5.07E-19 | 38.55446 |
| PDK4 | -2.6845349 | 6.362396085 | -7.2222159 | 2.07E-11 | 4.03E-10 | 15.48717 |
| SLC8A1 | -2.683218 | 7.23012288 | -8.6166067 | 6.93E-15 | 3.25E-13 | 23.31595 |
| HSPA6 | -2.6718757 | 6.568146779 | -10.7092 | 1.93E-20 | 4.69E-18 | 35.86376 |
| C19orf59 | -2.6659659 | 7.057019537 | -9.4671075 | 4.16E-17 | 3.53E-15 | 28.33261 |
| CDC42EP1 | -2.650734 | 4.801102446 | -8.6589362 | 5.39E-15 | 2.59E-13 | 23.56203 |
| FCGR2C | -2.6472408 | 6.673233603 | -9.2907575 | 1.21E-16 | 9.04E-15 | 27.28093 |
| TYMP | -2.6471187 | 9.03751387 | -11.784926 | 2.26E-23 | 2.04E-20 | 42.49388 |
| FFAR2 | -2.6431961 | 4.588668635 | -9.0786997 | 4.37E-16 | 2.83E-14 | 26.02375 |
| ANXA5 | -2.640592 | 9.516792033 | -9.4716904 | 4.04E-17 | 3.47E-15 | 28.36001 |
| SIRPB1 | -2.639747 | 9.445445713 | -11.517039 | 1.22E-22 | 6.39E-20 | 40.83775 |
| FAM20C | -2.6354484 | 6.655142028 | -9.3439729 | 8.79E-17 | 6.79E-15 | 27.59772 |
| FCER1G | -2.6312445 | 9.897035877 | -12.208592 | 1.56E-24 | 1.95E-21 | 45.11621 |
| HMOX1 | -2.6241276 | 7.769606132 | -10.040009 | 1.24E-18 | 1.70E-16 | 31.78271 |
| PTAFR | -2.6154664 | 8.742393464 | -10.793006 | 1.15E-20 | 3.00E-18 | 36.3778 |
| TGFBI | -2.6141321 | 7.094175526 | -6.5171651 | 9.24E-10 | 1.20E-08 | 11.78554 |
| C17orf87 | -2.6138432 | 6.897943337 | -12.406425 | 4.49E-25 | 7.29E-22 | 46.34121 |
| CD300C | -2.6082941 | 7.440113263 | -11.625114 | 6.19E-23 | 3.99E-20 | 41.50562 |
| SLC37A2 | -2.604319 | 7.31521926 | -11.014697 | 2.86E-21 | 9.48E-19 | 37.74012 |
| FGL2 | -2.5886684 | 10.39817932 | -9.3794 | 7.09E-17 | 5.64E-15 | 27.80889 |
| CR1 | -2.5824171 | 8.919409554 | -7.9479317 | 3.45E-13 | 1.03E-11 | 19.48908 |
| FAM198B | -2.5782809 | 8.837186848 | -8.1422728 | 1.12E-13 | 3.78E-12 | 20.58887 |
| SULF2 | -2.5727668 | 8.678751135 | -9.5095541 | 3.21E-17 | 2.83E-15 | 28.58653 |
| VNN2 | -2.5721114 | 7.7481422 | -9.0425392 | 5.44E-16 | 3.39E-14 | 25.81023 |
| PNPLA1 | -2.5694491 | 3.584930362 | -9.3395373 | 9.03E-17 | 6.94E-15 | 27.57129 |
| BASP1 | -2.5666521 | 8.055062604 | -8.2501525 | 5.98E-14 | 2.14E-12 | 21.20394 |
| CLEC4E | -2.5538371 | 4.747264888 | -6.1376099 | 6.56E-09 | 6.77E-08 | 9.882142 |
| CPM | -2.5425103 | 7.313128886 | -9.3013447 | 1.14E-16 | 8.59E-15 | 27.34392 |
| CST3 | -2.5337342 | 11.23243316 | -11.648233 | 5.35E-23 | 3.80E-20 | 41.64855 |
| NCF2 | -2.528301 | 10.25905766 | -9.7940508 | 5.62E-18 | 6.16E-16 | 30.29572 |
| C3 | -2.5248756 | 3.776013523 | -9.356167 | 8.16E-17 | 6.37E-15 | 27.67038 |
| EPHB2 | -2.522719 | 3.655649376 | -7.0238065 | 6.15E-11 | 1.06E-09 | 14.4253 |
| SYT17 | -2.5181064 | 3.689677757 | -8.7363264 | 3.40E-15 | 1.71E-13 | 24.013 |
| CD101 | -2.5178967 | 6.782823755 | -9.4032155 | 6.13E-17 | 5.00E-15 | 27.95097 |
| MNDA | -2.516707 | 10.72714731 | -8.8342036 | 1.90E-15 | 1.06E-13 | 24.58528 |
| CCR2 | -2.5143477 | 8.658152866 | -7.8633672 | 5.61E-13 | 1.60E-11 | 19.01399 |
| CD86 | -2.5139985 | 8.285083785 | -8.9126275 | 1.19E-15 | 7.08E-14 | 25.04533 |
| ABCC3 | -2.510564 | 5.201113961 | -8.0505904 | 1.91E-13 | 6.05E-12 | 20.06868 |
| DHRS9 | -2.5087463 | 7.222320225 | -7.8554275 | 5.87E-13 | 1.66E-11 | 18.96949 |
| ITGA7 | -2.5052718 | 6.521177385 | -9.1931513 | 2.19E-16 | 1.51E-14 | 26.70122 |
| GPBAR1 | -2.4879225 | 6.213981586 | -11.126501 | 1.42E-21 | 5.62E-19 | 38.42839 |
| ODF3B | -2.4826282 | 5.229622274 | -9.4675907 | 4.14E-17 | 3.53E-15 | 28.3355 |
| GLT1D1 | -2.4812854 | 4.609076383 | -6.353729 | 2.17E-09 | 2.56E-08 | 10.95771 |
| FXYD6 | -2.4744161 | 6.368361847 | -7.2344784 | 1.94E-11 | 3.81E-10 | 15.55328 |
| ARAP3 | -2.4698989 | 7.185929671 | -8.1013344 | 1.42E-13 | 4.68E-12 | 20.3563 |
| TICAM2 | -2.4667358 | 6.247192821 | -7.4420517 | 6.10E-12 | 1.33E-10 | 16.68068 |
| ADAP2 | -2.4666961 | 7.629275081 | -10.749899 | 1.50E-20 | 3.77E-18 | 36.11332 |
| KCNE1 | -2.4650951 | 5.430645033 | -8.1676395 | 9.68E-14 | 3.33E-12 | 20.73321 |
| PHACTR3 | -2.4647584 | 2.780540092 | -5.8567925 | 2.67E-08 | 2.37E-07 | 8.518849 |
| CTSH | -2.4642415 | 8.714514849 | -11.673409 | 4.56E-23 | 3.53E-20 | 41.8042 |
| HTR7 | -2.4614516 | 4.90654414 | -7.362972 | 9.49E-12 | 2.01E-10 | 16.24936 |
| CSTA | -2.4488039 | 8.247249419 | -7.2429732 | 1.85E-11 | 3.66E-10 | 15.59912 |
| EPHB3 | -2.4473919 | 4.749375547 | -7.5407378 | 3.51E-12 | 8.21E-11 | 17.22197 |
| AMICA1 | -2.4376155 | 9.314776859 | -8.6434357 | 5.91E-15 | 2.82E-13 | 23.47187 |
| LY86 | -2.4338008 | 7.955309017 | -7.14872 | 3.11E-11 | 5.81E-10 | 15.09208 |
| MYBPH | -2.429825 | 2.11770215 | -8.6376134 | 6.12E-15 | 2.90E-13 | 23.43801 |
| EMR3 | -2.4286856 | 4.803377635 | -7.0683505 | 4.82E-11 | 8.62E-10 | 14.66239 |
| CYP1B1 | -2.4222305 | 7.034656015 | -6.5521288 | 7.69E-10 | 1.02E-08 | 11.9642 |
| SIGLEC11 | -2.4110302 | 4.030720444 | -9.1840939 | 2.32E-16 | 1.58E-14 | 26.64752 |
| KCNJ2 | -2.4054456 | 3.714849514 | -7.2733293 | 1.56E-11 | 3.15E-10 | 15.76312 |
| SERINC2 | -2.4030686 | 5.482656551 | -7.161236 | 2.90E-11 | 5.46E-10 | 15.15922 |
| LILRB1 | -2.3944794 | 8.37660114 | -10.32794 | 2.08E-19 | 3.66E-17 | 33.53296 |
| RNASE6 | -2.3912545 | 9.03038972 | -9.1396662 | 3.03E-16 | 2.03E-14 | 26.38432 |
| MS4A14 | -2.386906 | 7.713343801 | -8.8039717 | 2.27E-15 | 1.23E-13 | 24.40829 |
| SGSH | -2.3741205 | 8.021171952 | -10.764353 | 1.37E-20 | 3.53E-18 | 36.20199 |
| SMPDL3A | -2.3735168 | 3.79408522 | -6.7825044 | 2.27E-10 | 3.40E-09 | 13.15467 |
| BPI | -2.3727235 | 6.000235591 | -4.9602782 | 1.81E-06 | 1.03E-05 | 4.453909 |
| FOLR3 | -2.3679935 | 2.844097878 | -7.1593155 | 2.93E-11 | 5.51E-10 | 15.14892 |
| CTSL1 | -2.3613869 | 6.747624969 | -8.254725 | 5.83E-14 | 2.11E-12 | 21.23008 |
| CLEC4A | -2.3588372 | 6.342028617 | -8.3029744 | 4.39E-14 | 1.67E-12 | 21.50626 |
| CXCL16 | -2.3507472 | 6.676920187 | -8.9478998 | 9.61E-16 | 5.79E-14 | 25.25266 |
| RAB39 | -2.3430985 | 3.888253562 | -9.2521885 | 1.53E-16 | 1.10E-14 | 27.05165 |
| FCGR2A | -2.3177057 | 9.851164827 | -9.4166065 | 5.65E-17 | 4.68E-15 | 28.0309 |
| CATSPER1 | -2.3075679 | 3.68134458 | -9.8355773 | 4.36E-18 | 5.08E-16 | 30.5462 |
| ZNF703 | -2.2997393 | 4.257783401 | -8.5019967 | 1.36E-14 | 6.03E-13 | 22.6518 |
| ARHGEF11 | -2.2989707 | 8.783863395 | -7.7864215 | 8.71E-13 | 2.35E-11 | 18.58359 |
| PID1 | -2.2928624 | 3.734254421 | -7.6449163 | 1.95E-12 | 4.77E-11 | 17.79695 |
| MOSC1 | -2.283312 | 7.291612396 | -6.2590455 | 3.53E-09 | 3.92E-08 | 10.48376 |
| C1QA | -2.2816473 | 5.839105502 | -5.8690465 | 2.52E-08 | 2.25E-07 | 8.577501 |
| METTL7B | -2.2702306 | 6.593849884 | -6.9011675 | 1.20E-10 | 1.93E-09 | 13.77658 |
| SCPEP1 | -2.2537729 | 9.828159933 | -8.908517 | 1.22E-15 | 7.20E-14 | 25.02118 |
| RIN2 | -2.2529608 | 6.760629485 | -9.0473411 | 5.28E-16 | 3.32E-14 | 25.83857 |
| CD68 | -2.2510386 | 11.33829883 | -11.647298 | 5.38E-23 | 3.80E-20 | 41.64276 |
| RASSF4 | -2.2461768 | 9.719846223 | -9.7763843 | 6.27E-18 | 6.78E-16 | 30.18923 |
| SERPINB10 | -2.2451753 | 6.036839136 | -5.9643193 | 1.57E-08 | 1.47E-07 | 9.03615 |
| LILRA1 | -2.2374299 | 8.018640932 | -9.7774319 | 6.23E-18 | 6.78E-16 | 30.19555 |
| SAMHD1 | -2.2365603 | 11.57587747 | -9.7743071 | 6.35E-18 | 6.82E-16 | 30.17672 |
| TMEM150B | -2.2348599 | 5.429264231 | -7.944328 | 3.52E-13 | 1.04E-11 | 19.46879 |
| ITGAM | -2.2324998 | 10.75922723 | -9.9527956 | 2.12E-18 | 2.75E-16 | 31.25452 |
| ZNF503 | -2.2237423 | 6.104604584 | -7.0902172 | 4.28E-11 | 7.72E-10 | 14.77905 |
| HNMT | -2.2120824 | 6.518507796 | -5.6354789 | 7.88E-08 | 6.25E-07 | 7.473239 |
| C9orf47 | -2.2015637 | 3.88446468 | -6.9384168 | 9.78E-11 | 1.61E-09 | 13.97298 |
| ANXA2 | -2.2002332 | 10.81576528 | -9.8456604 | 4.09E-18 | 4.85E-16 | 30.60706 |
| FCER2 | -2.1997603 | 6.652094752 | -7.5854889 | 2.72E-12 | 6.51E-11 | 17.46852 |
| GPR77 | -2.1896296 | 4.62763797 | -8.4483552 | 1.87E-14 | 7.93E-13 | 22.34206 |
| NID1 | -2.1885392 | 8.254638478 | -5.9091272 | 2.06E-08 | 1.88E-07 | 8.769883 |
| EGR2 | -2.1851309 | 6.45510559 | -7.557497 | 3.19E-12 | 7.52E-11 | 17.31422 |
| TNFRSF1B | -2.1820462 | 11.01711439 | -12.233203 | 1.34E-24 | 1.81E-21 | 45.2686 |
| MS4A7 | -2.1717953 | 9.230967145 | -9.297954 | 1.16E-16 | 8.73E-15 | 27.32374 |
| RAB31 | -2.1623376 | 9.550360225 | -7.8103031 | 7.60E-13 | 2.10E-11 | 18.71698 |
| ACVRL1 | -2.159071 | 4.119680445 | -7.0231343 | 6.17E-11 | 1.06E-09 | 14.42173 |
| RIN1 | -2.1572479 | 6.653311654 | -10.86423 | 7.34E-21 | 1.99E-18 | 36.8151 |
| FAM157B | -2.1553352 | 2.85547493 | -8.4018892 | 2.46E-14 | 1.01E-12 | 22.07434 |
| TNNT1 | -2.151742 | 3.165768216 | -5.2620426 | 4.60E-07 | 3.05E-06 | 5.770243 |
| MT1E | -2.1514016 | 3.414389948 | -7.2056991 | 2.27E-11 | 4.36E-10 | 15.39821 |
| CD4 | -2.1425867 | 10.73412869 | -10.988115 | 3.38E-21 | 1.04E-18 | 37.57659 |
| SGMS2 | -2.1407656 | 7.855703185 | -7.035494 | 5.77E-11 | 1.00E-09 | 14.48743 |
| OSCAR | -2.1336232 | 8.626178011 | -7.4437829 | 6.04E-12 | 1.32E-10 | 16.69014 |
| NPL | -2.1307797 | 6.812758086 | -7.9484089 | 3.44E-13 | 1.02E-11 | 19.49177 |
| STAB1 | -2.1251642 | 11.68335942 | -5.3132292 | 3.63E-07 | 2.46E-06 | 5.998942 |
| C19orf38 | -2.123313 | 7.640164625 | -9.6612952 | 1.27E-17 | 1.27E-15 | 29.49662 |
| FAM129B | -2.1226218 | 8.508072562 | -8.4736885 | 1.61E-14 | 7.01E-13 | 22.48825 |
| MMP17 | -2.1225032 | 6.238786011 | -7.2196019 | 2.10E-11 | 4.08E-10 | 15.47308 |
| RCN3 | -2.116944 | 5.748735477 | -8.0292857 | 2.16E-13 | 6.75E-12 | 19.94814 |
| SLC26A11 | -2.1145993 | 5.330085851 | -9.130922 | 3.19E-16 | 2.12E-14 | 26.33256 |
| CD36 | -2.1049539 | 10.92388413 | -6.4983821 | 1.02E-09 | 1.31E-08 | 11.68978 |
| KYNU | -2.1012647 | 7.453765021 | -5.9066604 | 2.09E-08 | 1.90E-07 | 8.758019 |
| ANXA2P2 | -2.0987407 | 9.411750414 | -9.8004947 | 5.40E-18 | 6.00E-16 | 30.33457 |
| CUEDC1 | -2.0976735 | 5.225818123 | -9.1940658 | 2.18E-16 | 1.51E-14 | 26.70665 |
| IRF8 | -2.0881282 | 10.14151937 | -7.2468125 | 1.81E-11 | 3.60E-10 | 15.61984 |
| SLC24A4 | -2.0870741 | 7.600008849 | -6.356632 | 2.13E-09 | 2.52E-08 | 10.9723 |
| PDE4A | -2.0863243 | 6.831083734 | -8.2697544 | 5.34E-14 | 1.95E-12 | 21.31604 |
| IGSF6 | -2.08528 | 7.983428028 | -10.012058 | 1.47E-18 | 1.95E-16 | 31.61332 |
| ABI3 | -2.0846941 | 6.938106775 | -9.010889 | 6.58E-16 | 4.04E-14 | 25.62356 |
| PTPRO | -2.0815519 | 6.612754729 | -8.2600761 | 5.65E-14 | 2.06E-12 | 21.26068 |
| CD209 | -2.0798881 | 3.404442623 | -6.4848494 | 1.09E-09 | 1.39E-08 | 11.62089 |
| VDR | -2.0761435 | 8.020138475 | -11.387609 | 2.76E-22 | 1.28E-19 | 40.03847 |
| C20orf118 | -2.0758407 | 7.535336209 | -10.356905 | 1.74E-19 | 3.24E-17 | 33.70954 |
| PLD2 | -2.0702895 | 6.572192137 | -9.0286836 | 5.91E-16 | 3.66E-14 | 25.72849 |
| HPSE | -2.0672124 | 6.115535316 | -7.5032038 | 4.33E-12 | 9.83E-11 | 17.0157 |
| UPP1 | -2.0622338 | 6.962747105 | -9.4418567 | 4.85E-17 | 4.05E-15 | 28.1817 |
| PILRA | -2.0618991 | 8.452175965 | -11.540592 | 1.05E-22 | 5.70E-20 | 40.98326 |
| IL13RA1 | -2.0606013 | 9.707300668 | -8.4455244 | 1.90E-14 | 8.04E-13 | 22.32574 |
| SGK1 | -2.0543939 | 8.127423204 | -8.1210273 | 1.27E-13 | 4.24E-12 | 20.46811 |
| RBP7 | -2.0521751 | 2.622958245 | -7.0206387 | 6.26E-11 | 1.07E-09 | 14.40847 |
| CD276 | -2.0511545 | 5.077185904 | -6.0543911 | 9.98E-09 | 9.77E-08 | 9.474007 |
| F5 | -2.0473168 | 7.087000076 | -7.0324821 | 5.87E-11 | 1.02E-09 | 14.47141 |
| DYSF | -2.0437259 | 9.544894593 | -5.9276116 | 1.88E-08 | 1.73E-07 | 8.858885 |
| ARHGAP24 | -2.0391089 | 5.892711202 | -8.283608 | 4.92E-14 | 1.81E-12 | 21.39533 |
| IER3 | -2.0386274 | 6.129453326 | -8.0189895 | 2.29E-13 | 7.13E-12 | 19.88994 |
| C5orf20 | -2.038412 | 6.291085645 | -4.6458316 | 7.12E-06 | 3.53E-05 | 3.14287 |
| ASGR1 | -2.037383 | 4.303135965 | -9.7987848 | 5.46E-18 | 6.03E-16 | 30.32426 |
| FCGR2B | -2.0362941 | 5.900516905 | -7.5788629 | 2.83E-12 | 6.73E-11 | 17.43197 |
| TDRD9 | -2.0359715 | 5.034456465 | -5.1206297 | 8.80E-07 | 5.42E-06 | 5.146498 |
| NRGN | -2.0355851 | 8.744223572 | -7.4143337 | 7.12E-12 | 1.53E-10 | 16.52925 |
| STX11 | -2.0297348 | 9.334940775 | -11.395568 | 2.62E-22 | 1.28E-19 | 40.08761 |
| CLEC1A | -2.0241502 | 2.31852146 | -8.7087721 | 4.01E-15 | 1.98E-13 | 23.85228 |
| GIMAP4 | -2.0233981 | 8.294678614 | -7.5563701 | 3.21E-12 | 7.56E-11 | 17.30802 |
| CKAP4 | -2.0203817 | 8.160900351 | -8.4225196 | 2.18E-14 | 9.11E-13 | 22.19314 |
| CPVL | -2.0185421 | 8.799688284 | -5.7862141 | 3.79E-08 | 3.25E-07 | 8.182567 |
| BCL6 | -2.0113134 | 9.649305155 | -9.9205893 | 2.58E-18 | 3.30E-16 | 31.05973 |
| PRKCDBP | -2.0083785 | 2.363045152 | -6.309214 | 2.73E-09 | 3.14E-08 | 10.73436 |
| ZNF366 | -2.0038923 | 3.471771332 | -7.0767686 | 4.61E-11 | 8.28E-10 | 14.70728 |
| IL1R2 | -2.0038918 | 3.144197933 | -4.7805551 | 3.99E-06 | 2.09E-05 | 3.696778 |
| SDC2 | -1.9972388 | 2.821639821 | -5.6054274 | 9.11E-08 | 7.13E-07 | 7.333294 |
| ANKRD34B | -1.9970968 | 2.092290933 | -7.0470997 | 5.42E-11 | 9.50E-10 | 14.54918 |
| HCK | -1.9940254 | 10.18346792 | -8.7980233 | 2.35E-15 | 1.26E-13 | 24.37349 |
| GRN | -1.9919112 | 12.80808979 | -12.053405 | 4.16E-24 | 4.83E-21 | 44.15539 |
| LY96 | -1.9905463 | 5.411815117 | -7.5841485 | 2.75E-12 | 6.55E-11 | 17.46112 |
| EXT1 | -1.9886949 | 5.802573715 | -8.1925861 | 8.37E-14 | 2.90E-12 | 20.87533 |
| DGAT2 | -1.9837953 | 6.077668526 | -7.1083797 | 3.87E-11 | 7.06E-10 | 14.8761 |
| MFSD2A | -1.9739411 | 6.402099957 | -10.329814 | 2.05E-19 | 3.66E-17 | 33.54438 |
| ALOX5 | -1.973219 | 9.396468761 | -7.665286 | 1.73E-12 | 4.32E-11 | 17.90979 |
| FCGR3A | -1.9726267 | 8.753639565 | -6.7604961 | 2.55E-10 | 3.78E-09 | 13.03997 |
| TNFAIP2 | -1.9723372 | 11.20135419 | -11.09789 | 1.70E-21 | 6.41E-19 | 38.25219 |
| AOAH | -1.970684 | 9.99650849 | -8.4420432 | 1.94E-14 | 8.17E-13 | 22.30566 |
| ALOX5AP | -1.9695298 | 9.503352522 | -8.7392008 | 3.34E-15 | 1.68E-13 | 24.02978 |
| LONRF3 | -1.9691204 | 5.778853858 | -8.3306895 | 3.74E-14 | 1.45E-12 | 21.66517 |
| HOXB3 | -1.9681195 | 8.640231121 | -3.9312715 | 0.0001264 | 0.0004535 | 0.41252 |
| ST14 | -1.9651302 | 7.032172675 | -5.8368807 | 2.95E-08 | 2.59E-07 | 8.423712 |
| TLR4 | -1.959481 | 10.21214434 | -8.2881316 | 4.79E-14 | 1.78E-12 | 21.42123 |
| IQSEC2 | -1.9572725 | 7.774671609 | -9.9251225 | 2.51E-18 | 3.23E-16 | 31.08714 |
| PPARG | -1.956568 | 3.723116396 | -5.2104948 | 5.84E-07 | 3.78E-06 | 5.541491 |
| DTX4 | -1.9537814 | 7.656712162 | -8.83457 | 1.89E-15 | 1.06E-13 | 24.58743 |
| SIGLEC16 | -1.9501843 | 4.707699668 | -9.8957412 | 3.01E-18 | 3.75E-16 | 30.90953 |
| GPR133 | -1.9477251 | 3.910178872 | -6.0114534 | 1.24E-08 | 1.19E-07 | 9.264769 |
| QPCT | -1.9476822 | 5.672951394 | -5.4503347 | 1.91E-07 | 1.39E-06 | 6.619017 |
| AIFM3 | -1.9476743 | 6.235358494 | -7.1816986 | 2.59E-11 | 4.91E-10 | 15.26911 |
| SLC22A15 | -1.9470667 | 7.850471704 | -5.17986 | 6.72E-07 | 4.27E-06 | 5.406294 |
| RGS2 | -1.9462726 | 9.505288988 | -7.6073183 | 2.41E-12 | 5.79E-11 | 17.58902 |
| RPH3A | -1.9459026 | 2.880099776 | -5.2810868 | 4.22E-07 | 2.82E-06 | 5.855152 |
| KRT23 | -1.94234 | 2.193395699 | -5.3566985 | 2.96E-07 | 2.06E-06 | 6.194363 |
| TNFSF12 | -1.9417763 | 6.478037957 | -8.6119218 | 7.12E-15 | 3.32E-13 | 23.28874 |
| CCR5 | -1.9375681 | 6.883069033 | -7.6170778 | 2.28E-12 | 5.51E-11 | 17.64295 |
| SLC43A2 | -1.9356169 | 8.866922066 | -10.06418 | 1.06E-18 | 1.53E-16 | 31.92926 |
| MMP14 | -1.932979 | 6.216735061 | -5.3394336 | 3.21E-07 | 2.21E-06 | 6.116615 |
| CEBPD | -1.9320114 | 9.148374691 | -8.356414 | 3.21E-14 | 1.27E-12 | 21.81285 |
| TMEM132A | -1.929083 | 3.825106712 | -8.6615613 | 5.31E-15 | 2.56E-13 | 23.5773 |
| LIPN | -1.9289244 | 2.783845487 | -6.7487587 | 2.71E-10 | 4.00E-09 | 12.97888 |
| SUCLG2 | -1.9250665 | 7.901184977 | -7.242323 | 1.85E-11 | 3.67E-10 | 15.59561 |
| ZNF385A | -1.9230039 | 9.649387891 | -11.551077 | 9.86E-23 | 5.70E-20 | 41.04805 |
| LGALS1 | -1.9222424 | 10.63922382 | -8.2565648 | 5.76E-14 | 2.09E-12 | 21.2406 |
| PTPRJ | -1.9200963 | 10.12567865 | -9.8791932 | 3.33E-18 | 4.06E-16 | 30.80955 |
| GABBR1 | -1.9118167 | 8.642613557 | -6.0663599 | 9.40E-09 | 9.25E-08 | 9.532495 |
| RNASE1 | -1.9092932 | 4.90465266 | -5.8961935 | 2.20E-08 | 1.99E-07 | 8.707712 |
| CD93 | -1.9058159 | 10.64130058 | -6.0782439 | 8.85E-09 | 8.76E-08 | 9.590639 |
| EMR4P | -1.9057645 | 5.595270344 | -5.0959318 | 9.85E-07 | 5.97E-06 | 5.038795 |
| FOLR2 | -1.9040309 | 3.664923075 | -6.5634612 | 7.25E-10 | 9.67E-09 | 12.02222 |
| TNFSF12-TNFSF13 | -1.9002608 | 8.943444498 | -8.7066511 | 4.06E-15 | 2.00E-13 | 23.83991 |
| TRPV4 | -1.8988918 | 1.77504076 | -6.5527153 | 7.67E-10 | 1.02E-08 | 11.9672 |
| PTGFR | -1.8924872 | 3.196133485 | -4.6809528 | 6.13E-06 | 3.08E-05 | 3.286123 |
| MEIS1 | -1.8905657 | 9.116531401 | -3.7537208 | 0.0002448 | 0.0008177 | -0.20867 |
| SASH1 | -1.8901372 | 3.656035378 | -4.9047052 | 2.32E-06 | 1.28E-05 | 4.217605 |
| HOXB6 | -1.8867807 | 5.235378103 | -3.2105252 | 0.0016068 | 0.0043217 | -1.95738 |
| TUBA4A | -1.8854175 | 8.86603947 | -9.0604497 | 4.88E-16 | 3.11E-14 | 25.91596 |
| TRPM4 | -1.885 | 4.144437931 | -4.5472302 | 1.08E-05 | 5.12E-05 | 2.745071 |
| CFP | -1.8749602 | 10.16838096 | -10.639959 | 2.98E-20 | 6.81E-18 | 35.43947 |
| THBS1 | -1.8732998 | 8.700887973 | -4.5485748 | 1.07E-05 | 5.09E-05 | 2.750452 |
| SORCS2 | -1.8728621 | 4.113839786 | -5.0340496 | 1.30E-06 | 7.64E-06 | 4.770574 |
| VNN1 | -1.868389 | 8.63336317 | -5.1363165 | 8.20E-07 | 5.08E-06 | 5.215097 |
| TIFAB | -1.8652959 | 5.013657618 | -4.5542383 | 1.05E-05 | 4.98E-05 | 2.77313 |
| C6orf97 | -1.863589 | 3.994462863 | -6.2528005 | 3.64E-09 | 4.03E-08 | 10.45265 |
| OLIG1 | -1.8605268 | 6.42030735 | -5.8012095 | 3.52E-08 | 3.04E-07 | 8.253796 |
| SCUBE1 | -1.8598194 | 6.580384722 | -4.7088957 | 5.44E-06 | 2.76E-05 | 3.400676 |
| PLA2G16 | -1.8573436 | 4.596817236 | -5.9671614 | 1.55E-08 | 1.46E-07 | 9.049904 |
| HOXB4 | -1.856478 | 7.254073537 | -4.1487039 | 5.46E-05 | 0.0002138 | 1.205071 |
| FCGR1A | -1.8563563 | 7.724167388 | -7.2294479 | 1.99E-11 | 3.90E-10 | 15.52615 |
| SLED1 | -1.8557326 | 4.530266015 | -7.457991 | 5.58E-12 | 1.23E-10 | 16.76788 |
| S100A11 | -1.8536258 | 10.30567915 | -10.42473 | 1.14E-19 | 2.28E-17 | 34.12337 |
| RUFY4 | -1.852906 | 3.584992103 | -6.5386373 | 8.26E-10 | 1.08E-08 | 11.89519 |
| RNASE2 | -1.8519882 | 11.5128999 | -6.5590761 | 7.42E-10 | 9.86E-09 | 11.99976 |
| NLRC4 | -1.8494117 | 7.670051581 | -8.3784134 | 2.82E-14 | 1.14E-12 | 21.93928 |
| GPR120 | -1.8430303 | 1.614585983 | -8.4138427 | 2.29E-14 | 9.52E-13 | 22.14316 |
| FCGRT | -1.8372982 | 10.05690146 | -8.7798181 | 2.63E-15 | 1.37E-13 | 24.26703 |
| OR52K2 | -1.8340714 | 3.038171402 | -6.5397578 | 8.21E-10 | 1.07E-08 | 11.90092 |
| HOXB2 | -1.8335764 | 6.909171639 | -4.5121889 | 1.25E-05 | 5.80E-05 | 2.605271 |
| CRIP3 | -1.8324344 | 2.619842339 | -9.3865041 | 6.79E-17 | 5.43E-15 | 27.85126 |
| DOK2 | -1.827331 | 8.759014451 | -10.936526 | 4.67E-21 | 1.33E-18 | 37.25936 |
| CES4 | -1.8212426 | 1.27146254 | -6.8085821 | 1.97E-10 | 3.00E-09 | 13.29085 |
| PPFIBP2 | -1.8176039 | 6.312095333 | -7.73014 | 1.20E-12 | 3.11E-11 | 18.26995 |
| P2RY13 | -1.8143809 | 8.344863446 | -7.2682737 | 1.61E-11 | 3.23E-10 | 15.73578 |
| S100A6 | -1.8141391 | 10.17322357 | -11.060273 | 2.15E-21 | 7.76E-19 | 38.0206 |
| FCGR1C | -1.8123658 | 6.289191294 | -7.6554946 | 1.83E-12 | 4.54E-11 | 17.85553 |
| FCGR1B | -1.8086107 | 7.854195811 | -7.9035995 | 4.45E-13 | 1.30E-11 | 19.23975 |
| IL10RA | -1.8052545 | 10.29292596 | -8.868422 | 1.55E-15 | 8.80E-14 | 24.78585 |
| C1orf162 | -1.8052178 | 9.710513037 | -10.101419 | 8.46E-19 | 1.27E-16 | 32.15519 |
| IGF2R | -1.8025298 | 10.43984686 | -7.9580436 | 3.26E-13 | 9.75E-12 | 19.54604 |
| COPZ2 | -1.8022896 | 2.283983168 | -6.075343 | 8.98E-09 | 8.88E-08 | 9.57644 |
| TNNI2 | -1.8002692 | 4.133124214 | -5.7612411 | 4.28E-08 | 3.63E-07 | 8.064208 |
| S100A10 | -1.7993643 | 9.714752672 | -6.457465 | 1.26E-09 | 1.58E-08 | 11.48174 |
| RRAS | -1.7960475 | 6.916826865 | -9.3374385 | 9.15E-17 | 7.00E-15 | 27.55879 |
| TMEM38A | -1.7960448 | 2.962206239 | -6.9923725 | 7.30E-11 | 1.24E-09 | 14.25846 |
| KIAA0513 | -1.7955295 | 9.146292714 | -9.3533193 | 8.31E-17 | 6.45E-15 | 27.65341 |
| RTN1 | -1.7914312 | 4.314287635 | -4.9914966 | 1.58E-06 | 9.07E-06 | 4.587502 |
| TYROBP | -1.7899694 | 10.80143266 | -9.3724968 | 7.39E-17 | 5.82E-15 | 27.76772 |
| LST1 | -1.7873442 | 9.500604016 | -8.961824 | 8.84E-16 | 5.41E-14 | 25.33458 |
| NACC2 | -1.7793009 | 6.036806271 | -7.9752984 | 2.95E-13 | 8.94E-12 | 19.64329 |
| MRAS | -1.778565 | 4.856528216 | -6.1271275 | 6.91E-09 | 7.09E-08 | 9.830544 |
| VENTX | -1.7782866 | 5.226590704 | -4.4164759 | 1.86E-05 | 8.21E-05 | 2.227667 |
| ADM | -1.7764939 | 4.553478674 | -5.9372542 | 1.79E-08 | 1.66E-07 | 8.905384 |
| TMEM176A | -1.7753836 | 5.527496561 | -4.2699574 | 3.37E-05 | 0.0001392 | 1.661874 |
| CLTCL1 | -1.7739391 | 6.815518503 | -4.8641579 | 2.77E-06 | 1.51E-05 | 4.046422 |
| CARD14 | -1.7662475 | 3.759888621 | -8.3973764 | 2.53E-14 | 1.03E-12 | 22.04836 |
| GIMAP8 | -1.7640043 | 8.001537147 | -6.6050051 | 5.82E-10 | 7.96E-09 | 12.23541 |
| CRYBG3 | -1.762299 | 7.30514888 | -6.3032422 | 2.81E-09 | 3.22E-08 | 10.70447 |
| DAGLA | -1.7583611 | 5.966744667 | -5.9657633 | 1.56E-08 | 1.46E-07 | 9.043137 |
| TBC1D9 | -1.7582045 | 7.332886432 | -7.0372528 | 5.72E-11 | 9.95E-10 | 14.49679 |
| FBN2 | -1.7521778 | 6.818139351 | -4.256739 | 3.56E-05 | 0.0001458 | 1.611569 |
| DES | -1.744322 | 1.976641965 | -5.2413513 | 5.07E-07 | 3.33E-06 | 5.678233 |
| C10orf54 | -1.7420516 | 12.26458668 | -10.748792 | 1.51E-20 | 3.77E-18 | 36.10653 |
| ADAMDEC1 | -1.7415105 | 3.200384213 | -5.3342331 | 3.29E-07 | 2.26E-06 | 6.09323 |
| OASL | -1.7386068 | 5.334416288 | -7.2301435 | 1.98E-11 | 3.89E-10 | 15.52991 |
| ELOVL3 | -1.7374867 | 2.602632124 | -6.4199225 | 1.54E-09 | 1.88E-08 | 11.29152 |
| LILRB5 | -1.7324263 | 3.963851738 | -6.3002968 | 2.85E-09 | 3.25E-08 | 10.68973 |
| CD200R1 | -1.7265312 | 5.700697621 | -6.7012895 | 3.49E-10 | 5.02E-09 | 12.7324 |
| G0S2 | -1.7261629 | 3.98250528 | -5.0299643 | 1.33E-06 | 7.77E-06 | 4.75295 |
| AIM2 | -1.7260497 | 4.769170819 | -7.4864562 | 4.76E-12 | 1.07E-10 | 16.92382 |
| ITGAX | -1.7260434 | 11.02927194 | -9.2713211 | 1.37E-16 | 9.98E-15 | 27.16535 |
| CECR1 | -1.7242471 | 12.00519605 | -8.5562108 | 9.91E-15 | 4.48E-13 | 22.96557 |
| SDC3 | -1.7222584 | 5.953414865 | -5.205655 | 5.97E-07 | 3.85E-06 | 5.520095 |
| CXCR2P1 | -1.7214273 | 5.370745584 | -4.9863152 | 1.61E-06 | 9.26E-06 | 4.565287 |
| BST1 | -1.7183743 | 8.717910092 | -7.8335838 | 6.65E-13 | 1.86E-11 | 18.84718 |
| CTSL2 | -1.7176484 | 1.405296637 | -6.2376822 | 3.94E-09 | 4.31E-08 | 10.37741 |
| TMEM176B | -1.7162042 | 6.315738982 | -3.9102188 | 0.0001369 | 0.0004872 | 0.337629 |
| CEBPB | -1.7081589 | 9.06391797 | -10.022102 | 1.38E-18 | 1.85E-16 | 31.67418 |
| VSIG4 | -1.7071307 | 6.706366546 | -4.6482859 | 7.04E-06 | 3.49E-05 | 3.152854 |
| SMARCD3 | -1.7068375 | 7.545995542 | -8.0287125 | 2.17E-13 | 6.76E-12 | 19.9449 |
| CLEC4G | -1.7016113 | 3.157086168 | -5.365215 | 2.85E-07 | 1.99E-06 | 6.232778 |
| HOXB5 | -1.7011617 | 4.857823284 | -3.1248386 | 0.0021192 | 0.0055419 | -2.2116 |
| NHS | -1.7009333 | 4.670918913 | -6.3588228 | 2.11E-09 | 2.50E-08 | 10.98332 |
| POU2F2 | -1.6977624 | 10.61403215 | -9.6676989 | 1.22E-17 | 1.23E-15 | 29.53511 |
| FZD2 | -1.6967597 | 5.797099295 | -5.2798967 | 4.24E-07 | 2.83E-06 | 5.849839 |
| IL17RA | -1.6934365 | 10.64535187 | -9.6482649 | 1.37E-17 | 1.37E-15 | 29.41833 |
| FEZ1 | -1.6911488 | 3.333079676 | -5.94527 | 1.72E-08 | 1.60E-07 | 8.944073 |
| ACOX2 | -1.6902179 | 2.672518962 | -5.5364006 | 1.27E-07 | 9.61E-07 | 7.013733 |
| CD163L1 | -1.6897686 | 2.88687695 | -6.2364592 | 3.96E-09 | 4.33E-08 | 10.37132 |
| PPARGC1A | -1.6846147 | 4.29102979 | -3.3034885 | 0.0011825 | 0.0032902 | -1.67479 |
| EPS8 | -1.6843997 | 6.602993146 | -5.0225097 | 1.37E-06 | 8.00E-06 | 4.720816 |
| VIPR1 | -1.6838896 | 6.430668527 | -6.9908619 | 7.36E-11 | 1.25E-09 | 14.25045 |
| TMTC1 | -1.6820743 | 3.105219664 | -5.0399933 | 1.27E-06 | 7.47E-06 | 4.796234 |
| TNFSF10 | -1.6801882 | 9.292223044 | -9.0406826 | 5.50E-16 | 3.42E-14 | 25.79928 |
| RPGRIP1 | -1.6798035 | 3.523755786 | -6.7854746 | 2.23E-10 | 3.35E-09 | 13.17017 |
| MFSD7 | -1.6772393 | 5.591549088 | -7.4216073 | 6.84E-12 | 1.48E-10 | 16.56896 |
| LOC729603 | -1.6767157 | 6.06837688 | -7.8751093 | 5.24E-13 | 1.50E-11 | 19.07983 |
| ACPP | -1.6764318 | 7.945105122 | -7.9560226 | 3.29E-13 | 9.84E-12 | 19.53465 |
| IL10 | -1.6755821 | 3.038266322 | -5.0220323 | 1.37E-06 | 8.01E-06 | 4.71876 |
| SLC2A6 | -1.6744606 | 7.273653738 | -7.7176717 | 1.29E-12 | 3.32E-11 | 18.20061 |
| MYO1C | -1.6682873 | 7.227498402 | -8.252689 | 5.90E-14 | 2.12E-12 | 21.21844 |
| CEACAM3 | -1.6641987 | 2.826139239 | -5.0822467 | 1.05E-06 | 6.31E-06 | 4.979277 |
| LMNA | -1.6624561 | 10.01701695 | -5.9371681 | 1.80E-08 | 1.66E-07 | 8.904968 |
| SLC46A2 | -1.6583346 | 5.727310014 | -8.2050621 | 7.78E-14 | 2.72E-12 | 20.94647 |
| CSF2RA | -1.6573007 | 8.941611611 | -9.2794388 | 1.30E-16 | 9.59E-15 | 27.21362 |
| SLC1A3 | -1.6519941 | 7.593638963 | -6.7646849 | 2.49E-10 | 3.71E-09 | 13.06178 |
| UGGT2 | -1.6518018 | 4.940487647 | -3.2838283 | 0.0012624 | 0.0034827 | -1.73514 |
| ANXA2P1 | -1.6469796 | 4.869280528 | -8.029417 | 2.16E-13 | 6.75E-12 | 19.94889 |
| SAMD4A | -1.6440436 | 5.180935675 | -6.9819611 | 7.72E-11 | 1.30E-09 | 14.20328 |
| CREB5 | -1.6400995 | 8.062923298 | -6.2201069 | 4.31E-09 | 4.67E-08 | 10.29008 |
| KLK1 | -1.6385388 | 1.848531882 | -6.7776483 | 2.32E-10 | 3.49E-09 | 13.12935 |
| ADAMTS5 | -1.6365159 | 2.902019119 | -4.5365879 | 1.13E-05 | 5.32E-05 | 2.702525 |
| HRH2 | -1.6364277 | 8.736723632 | -8.3484493 | 3.37E-14 | 1.32E-12 | 21.76711 |
| PDZD7 | -1.633169 | 4.11912802 | -6.3056659 | 2.78E-09 | 3.19E-08 | 10.7166 |
| RNASE3 | -1.6310168 | 8.566319319 | -4.8455844 | 3.00E-06 | 1.62E-05 | 3.968357 |
| MMP25 | -1.6287684 | 6.744461822 | -6.5756348 | 6.80E-10 | 9.11E-09 | 12.08461 |
| KCNMB1 | -1.6271631 | 6.423666787 | -6.3257268 | 2.50E-09 | 2.91E-08 | 10.8171 |
| PRKCA | -1.626738 | 8.984786655 | -6.9115035 | 1.13E-10 | 1.83E-09 | 13.83102 |
| C9orf110 | -1.6249396 | 5.808804823 | -5.679654 | 6.37E-08 | 5.16E-07 | 7.679849 |
| CAMK1 | -1.620968 | 6.8470201 | -7.0407393 | 5.61E-11 | 9.79E-10 | 14.51533 |
| LOC728392 | -1.6205514 | 6.884204179 | -9.2329095 | 1.72E-16 | 1.22E-14 | 26.93715 |
| TNF | -1.6174121 | 5.374530477 | -5.9195598 | 1.96E-08 | 1.80E-07 | 8.820095 |
| TFEB | -1.6155304 | 8.344849728 | -10.394789 | 1.37E-19 | 2.71E-17 | 33.94063 |
| CASP5 | -1.6144333 | 2.102888498 | -6.492528 | 1.05E-09 | 1.34E-08 | 11.65997 |
| ADAP1 | -1.612547 | 8.883578432 | -9.4480964 | 4.67E-17 | 3.92E-15 | 28.21898 |
| CCL23 | -1.6096329 | 4.703220809 | -3.9912237 | 0.0001006 | 0.0003694 | 0.627591 |
| LYNX1 | -1.6088265 | 3.680236632 | -5.8502747 | 2.76E-08 | 2.44E-07 | 8.487685 |
| ARHGAP29 | -1.608275 | 3.299513982 | -5.2066379 | 5.94E-07 | 3.83E-06 | 5.52444 |
| NUAK2 | -1.606794 | 6.741774509 | -7.531732 | 3.69E-12 | 8.58E-11 | 17.17244 |
| FAM127A | -1.6053688 | 6.338497325 | -3.7597776 | 0.0002394 | 0.0008013 | -0.18787 |
| NINJ1 | -1.6050795 | 8.520681748 | -8.8032778 | 2.28E-15 | 1.23E-13 | 24.40423 |
| RYR1 | -1.601713 | 5.010120299 | -4.9907797 | 1.58E-06 | 9.09E-06 | 4.584427 |
| SIRPB2 | -1.599859 | 9.929834074 | -6.5515192 | 7.72E-10 | 1.02E-08 | 11.96108 |
| TMED7-TICAM2 | -1.5979242 | 4.055021007 | -3.7487246 | 0.0002493 | 0.0008313 | -0.2258 |
| FGD4 | -1.594893 | 8.865089341 | -6.4319283 | 1.44E-09 | 1.79E-08 | 11.35228 |
| UPK3A | -1.5913568 | 1.5387516 | -6.5842077 | 6.50E-10 | 8.77E-09 | 12.12859 |
| CECR6 | -1.5897559 | 6.269556914 | -4.9372827 | 2.01E-06 | 1.13E-05 | 4.355893 |
| KCNK13 | -1.5858569 | 1.722233132 | -6.3030356 | 2.81E-09 | 3.22E-08 | 10.70343 |
| ZMYND15 | -1.5850634 | 2.221286083 | -6.4283278 | 1.47E-09 | 1.82E-08 | 11.33405 |
| HP | -1.5846028 | 6.094984541 | -5.3426659 | 3.17E-07 | 2.19E-06 | 6.131158 |
| LRG1 | -1.5835796 | 7.156076609 | -5.6765779 | 6.46E-08 | 5.23E-07 | 7.665428 |
| GPR109B | -1.5820552 | 5.733913713 | -7.4787076 | 4.97E-12 | 1.11E-10 | 16.88134 |
| FAM151B | -1.575086 | 5.214247361 | -7.6477133 | 1.92E-12 | 4.71E-11 | 17.81243 |
| PLAUR | -1.5742598 | 8.841949416 | -9.5455553 | 2.58E-17 | 2.33E-15 | 28.80212 |
| SHROOM1 | -1.5724348 | 3.784553138 | -5.7550137 | 4.41E-08 | 3.73E-07 | 8.034746 |
| CPAMD8 | -1.5723809 | 4.540965205 | -5.3723189 | 2.76E-07 | 1.93E-06 | 6.264854 |
| TNFRSF8 | -1.5710187 | 5.153497019 | -6.5421226 | 8.11E-10 | 1.06E-08 | 11.91301 |
| CTSS | -1.5706475 | 12.59867047 | -9.1003616 | 3.84E-16 | 2.50E-14 | 26.15178 |
| C11orf75 | -1.5669513 | 6.75357969 | -9.3161467 | 1.04E-16 | 7.89E-15 | 27.43201 |
| ONECUT2 | -1.5663612 | 2.362945222 | -4.2182024 | 4.15E-05 | 0.0001672 | 1.465616 |
| RXFP2 | -1.5638067 | 4.321974629 | -4.5657815 | 9.99E-06 | 4.78E-05 | 2.819418 |
| 43891 | -1.5638059 | 8.085756185 | -7.7193125 | 1.28E-12 | 3.30E-11 | 18.20973 |
| KIF26B | -1.5613124 | 6.824056934 | -5.3424067 | 3.17E-07 | 2.19E-06 | 6.129992 |
| NR4A1 | -1.5609585 | 8.535956112 | -5.3755545 | 2.71E-07 | 1.90E-06 | 6.279472 |
| AGPAT9 | -1.5589931 | 7.118233559 | -4.7385259 | 4.78E-06 | 2.46E-05 | 3.522705 |
| PCSK5 | -1.5534129 | 4.043254156 | -5.9571316 | 1.63E-08 | 1.52E-07 | 9.001386 |
| SLC2A9 | -1.553142 | 7.243169084 | -7.6573857 | 1.81E-12 | 4.50E-11 | 17.86601 |
| HVCN1 | -1.5517176 | 9.009649999 | -10.371493 | 1.59E-19 | 3.03E-17 | 33.79851 |
| PPFIA4 | -1.5508289 | 5.326957765 | -5.4071956 | 2.34E-07 | 1.66E-06 | 6.422751 |
| GPR84 | -1.5499027 | 4.542077122 | -6.1620547 | 5.79E-09 | 6.06E-08 | 10.00267 |
| TBC1D12 | -1.5496245 | 4.742271502 | -4.7743706 | 4.10E-06 | 2.15E-05 | 3.671092 |
| CAPN2 | -1.5489246 | 10.84743236 | -10.622371 | 3.33E-20 | 7.50E-18 | 35.33177 |
| PTGFRN | -1.5485983 | 5.681726459 | -5.1175843 | 8.93E-07 | 5.49E-06 | 5.133197 |
| VSIG2 | -1.5484333 | 3.761860446 | -6.2660093 | 3.40E-09 | 3.81E-08 | 10.51847 |
| ITGB2 | -1.5480375 | 13.15864018 | -10.359326 | 1.71E-19 | 3.23E-17 | 33.7243 |
| CFD | -1.5446916 | 10.51641033 | -4.9725036 | 1.71E-06 | 9.77E-06 | 4.506153 |
| LFNG | -1.5426153 | 8.587518589 | -8.5899377 | 8.11E-15 | 3.72E-13 | 23.16112 |
| PSAP | -1.5375521 | 13.97550652 | -11.259907 | 6.15E-22 | 2.70E-19 | 39.25059 |
| PSTPIP1 | -1.5370963 | 8.876432307 | -10.17149 | 5.48E-19 | 8.55E-17 | 32.58076 |
| LILRA2 | -1.5368865 | 9.781559334 | -7.6264757 | 2.16E-12 | 5.25E-11 | 17.69491 |
| CXCL10 | -1.5365885 | 3.202420132 | -5.477957 | 1.67E-07 | 1.23E-06 | 6.745244 |
| GPR124 | -1.536515 | 9.173209101 | -5.2813704 | 4.21E-07 | 2.81E-06 | 5.856418 |
| C1orf127 | -1.5356169 | 3.963997315 | -6.6531822 | 4.51E-10 | 6.36E-09 | 12.48359 |
| PKIB | -1.5352529 | 4.233754638 | -5.05936 | 1.16E-06 | 6.94E-06 | 4.879995 |
| KIF1C | -1.5333893 | 8.22052376 | -10.424879 | 1.14E-19 | 2.28E-17 | 34.12428 |
| CTSZ | -1.5319281 | 11.11028797 | -12.33807 | 6.92E-25 | 1.02E-21 | 45.91795 |
| BCL2A1 | -1.5298686 | 7.510591821 | -6.3543766 | 2.16E-09 | 2.55E-08 | 10.96096 |
| RAB11FIP5 | -1.5294374 | 5.339616052 | -5.2900483 | 4.04E-07 | 2.71E-06 | 5.895181 |
| C5orf32 | -1.528692 | 6.469779564 | -7.7607285 | 1.01E-12 | 2.67E-11 | 18.44029 |
| CDKN2B | -1.5255296 | 4.736441489 | -5.3149941 | 3.60E-07 | 2.44E-06 | 6.006855 |
| CSF1R | -1.5246737 | 11.28478876 | -7.1375327 | 3.30E-11 | 6.12E-10 | 15.03212 |
| SLCO4C1 | -1.5238973 | 7.433569331 | -6.080006 | 8.77E-09 | 8.70E-08 | 9.599267 |
| NLRP1 | -1.5170866 | 11.04510838 | -9.1714218 | 2.50E-16 | 1.69E-14 | 26.57241 |
| PLA2G7 | -1.516427 | 2.646281997 | -4.3839077 | 2.12E-05 | 9.22E-05 | 2.100613 |
| ARHGAP31 | -1.5146766 | 7.879430363 | -6.4365378 | 1.41E-09 | 1.75E-08 | 11.37563 |
| OPLAH | -1.5126187 | 4.627884521 | -5.583864 | 1.01E-07 | 7.84E-07 | 7.233183 |
| SLC16A6 | -1.5096325 | 4.363786265 | -5.8016096 | 3.51E-08 | 3.03E-07 | 8.255698 |
| PTPRH | -1.5094878 | 2.92311846 | -4.7571022 | 4.42E-06 | 2.29E-05 | 3.599502 |
| TPPP3 | -1.508509 | 5.513748084 | -4.2527942 | 3.61E-05 | 0.0001478 | 1.59658 |
| WLS | -1.5082256 | 2.948177735 | -4.3881692 | 2.09E-05 | 9.08E-05 | 2.117196 |
| C1QC | -1.5064947 | 5.242570999 | -3.6545147 | 0.0003508 | 0.0011227 | -0.54533 |
| HNRPLL | -1.5046052 | 7.444662971 | -5.4164279 | 2.24E-07 | 1.60E-06 | 6.464664 |
| ANKRD35 | -1.5046023 | 3.58039897 | -4.9553368 | 1.85E-06 | 1.05E-05 | 4.432819 |
| COTL1 | -1.5034793 | 11.91513962 | -11.020207 | 2.77E-21 | 9.48E-19 | 37.77402 |
| C1QB | -1.5000723 | 5.585274666 | -3.6056765 | 0.0004176 | 0.0013113 | -0.70828 |
| ZBTB7B | -1.4999362 | 10.22578963 | -10.925019 | 5.02E-21 | 1.40E-18 | 37.18862 |
| SLITRK5 | -1.4990335 | 4.273413334 | -3.058469 | 0.002616 | 0.0066931 | -2.40433 |
| GPNMB | -1.497862 | 3.948247473 | -4.6441497 | 7.17E-06 | 3.55E-05 | 3.13603 |
| NHSL2 | -1.4970691 | 8.370408635 | -5.4579716 | 1.84E-07 | 1.34E-06 | 6.653873 |
| UBTD1 | -1.4961246 | 6.289364144 | -9.6928499 | 1.05E-17 | 1.07E-15 | 29.68633 |
| CCL22 | -1.495359 | 1.634406293 | -6.3236815 | 2.53E-09 | 2.94E-08 | 10.80685 |
| HLA-DQB1 | -1.4936138 | 9.672437966 | -4.3983126 | 2.00E-05 | 8.75E-05 | 2.156719 |
| CT45A1 | -1.4922266 | 1.960197627 | -2.9209511 | 0.0040037 | 0.0097416 | -2.79195 |
| S100P | -1.4918997 | 4.920593064 | -4.22852 | 3.98E-05 | 0.0001612 | 1.50459 |
| COL9A2 | -1.4917801 | 7.618260397 | -5.584541 | 1.01E-07 | 7.82E-07 | 7.236322 |
| C17orf91 | -1.4903401 | 6.399904428 | -8.3595723 | 3.15E-14 | 1.25E-12 | 21.831 |
| SEMA6B | -1.4846028 | 4.576024669 | -5.5959372 | 9.54E-08 | 7.44E-07 | 7.289202 |
| GUCY2C | -1.4845 | 1.405530206 | -8.0846879 | 1.57E-13 | 5.10E-12 | 20.26186 |
| TSHZ3 | -1.4842654 | 5.938778985 | -4.5564406 | 1.04E-05 | 4.95E-05 | 2.781954 |
| FLJ45445 | -1.4782577 | 6.02407409 | -7.1340497 | 3.37E-11 | 6.21E-10 | 15.01347 |
| AZU1 | -1.4771439 | 10.9330131 | -3.4444568 | 0.0007338 | 0.002163 | -1.23294 |
| CAMP | -1.4758772 | 2.807068165 | -4.3740687 | 2.21E-05 | 9.56E-05 | 2.062373 |
| TRIM7 | -1.4751639 | 4.87104505 | -6.9231106 | 1.06E-10 | 1.73E-09 | 13.89221 |
| LGALS12 | -1.4711594 | 6.071504804 | -4.4746688 | 1.46E-05 | 6.67E-05 | 2.456503 |
| BAG3 | -1.4700901 | 5.45555045 | -5.7541806 | 4.43E-08 | 3.74E-07 | 8.030805 |
| C9orf109 | -1.467171 | 2.645231408 | -6.1994884 | 4.78E-09 | 5.12E-08 | 10.18781 |
| MICALCL | -1.4664522 | 4.649768506 | -6.6313692 | 5.06E-10 | 7.06E-09 | 12.3711 |
| DEFB1 | -1.4658121 | 3.063758689 | -2.9695538 | 0.0034503 | 0.0085528 | -2.65677 |
| SNX24 | -1.4653282 | 5.237266646 | -6.3935414 | 1.76E-09 | 2.13E-08 | 11.15824 |
| SIGLEC14 | -1.4645381 | 8.233039816 | -5.3148163 | 3.60E-07 | 2.45E-06 | 6.006058 |
| THBS4 | -1.4637519 | 3.674276328 | -4.5051515 | 1.29E-05 | 5.95E-05 | 2.577295 |
| GNGT2 | -1.4626179 | 3.978348821 | -7.0295297 | 5.96E-11 | 1.03E-09 | 14.45572 |
| EMR1 | -1.461757 | 8.625050715 | -4.8939322 | 2.43E-06 | 1.34E-05 | 4.172021 |
| METRNL | -1.4603102 | 7.49079383 | -6.9600128 | 8.70E-11 | 1.45E-09 | 14.08711 |
| CXCR2 | -1.4596229 | 6.837145362 | -5.6232785 | 8.36E-08 | 6.59E-07 | 7.416364 |
| CDH23 | -1.4576085 | 7.050611379 | -6.9383908 | 9.78E-11 | 1.61E-09 | 13.97285 |
| RXRA | -1.4542857 | 10.22727096 | -8.278235 | 5.08E-14 | 1.87E-12 | 21.36457 |
| MRVI1 | -1.4515257 | 6.308848338 | -7.139527 | 3.27E-11 | 6.06E-10 | 15.04281 |
| SATB2 | -1.4499531 | 4.718773807 | -4.9441808 | 1.94E-06 | 1.09E-05 | 4.385261 |
| EHD4 | -1.4487005 | 8.525274782 | -10.308017 | 2.35E-19 | 3.97E-17 | 33.41156 |
| LAMB3 | -1.4485755 | 2.839367969 | -5.7993366 | 3.55E-08 | 3.06E-07 | 8.244894 |
| STS | -1.4457282 | 8.250858695 | -8.8962481 | 1.31E-15 | 7.69E-14 | 24.94914 |
| FRMD3 | -1.4448387 | 4.956364363 | -5.308612 | 3.71E-07 | 2.51E-06 | 5.97825 |
| LOC388242 | -1.4445801 | 2.439547479 | -7.6953767 | 1.46E-12 | 3.71E-11 | 18.07673 |
| FLVCR2 | -1.4440338 | 7.126095035 | -7.8769182 | 5.19E-13 | 1.49E-11 | 19.08998 |
| ARTN | -1.4426977 | 5.750503011 | -4.6338691 | 7.49E-06 | 3.68E-05 | 3.094263 |
| OTOA | -1.4422123 | 2.55573293 | -4.6769908 | 6.23E-06 | 3.13E-05 | 3.269921 |
| PLEC | -1.4397864 | 12.61260241 | -9.6451155 | 1.40E-17 | 1.38E-15 | 29.39941 |
| TREML4 | -1.4397097 | 3.430455459 | -4.6057548 | 8.44E-06 | 4.10E-05 | 2.980401 |
| C17orf60 | -1.4372446 | 5.923548336 | -7.6374571 | 2.03E-12 | 4.95E-11 | 17.75566 |
| CCL3 | -1.4352772 | 5.736752598 | -6.2597316 | 3.52E-09 | 3.91E-08 | 10.48718 |
| GLIPR2 | -1.4309806 | 10.04391458 | -9.0900885 | 4.08E-16 | 2.65E-14 | 26.09105 |
| SH3BP5 | -1.4309165 | 7.492294176 | -5.0733844 | 1.09E-06 | 6.54E-06 | 4.940794 |
| PMM1 | -1.4304861 | 6.073167745 | -8.1562273 | 1.03E-13 | 3.53E-12 | 20.66825 |
| SIRPA | -1.429247 | 10.8753656 | -10.050011 | 1.16E-18 | 1.65E-16 | 31.84334 |
| KCNH3 | -1.4257493 | 4.870382726 | -6.1304304 | 6.80E-09 | 7.00E-08 | 9.846796 |
| CDKN1A | -1.4227344 | 9.241049354 | -6.599407 | 6.00E-10 | 8.16E-09 | 12.20664 |
| SLPI | -1.4212908 | 3.584714804 | -3.7017563 | 0.0002958 | 0.0009692 | -0.38595 |
| GIMAP1 | -1.4205191 | 7.221704314 | -8.7542456 | 3.06E-15 | 1.57E-13 | 24.11761 |
| ANKRD22 | -1.4196653 | 5.317343017 | -4.4328547 | 1.74E-05 | 7.74E-05 | 2.29184 |
| RHOU | -1.4183573 | 8.123809537 | -5.6696924 | 6.68E-08 | 5.39E-07 | 7.633166 |
| C2orf58 | -1.4163233 | 1.569055947 | -8.1251439 | 1.24E-13 | 4.15E-12 | 20.4915 |
| S100A4 | -1.4152876 | 11.15829617 | -9.1738181 | 2.46E-16 | 1.67E-14 | 26.58661 |
| HORMAD1 | -1.4148919 | 3.334386783 | -6.2401726 | 3.89E-09 | 4.27E-08 | 10.38979 |
| CSMD1 | -1.4148811 | 3.531770954 | -3.4343476 | 0.0007597 | 0.0022309 | -1.26516 |
| HOXB7 | -1.414052 | 3.179114636 | -3.6268955 | 0.0003872 | 0.0012256 | -0.63771 |
| FAM110B | -1.4136499 | 4.067364171 | -3.5265496 | 0.000552 | 0.0016781 | -0.96834 |
| DACH1 | -1.4120066 | 7.569833465 | -3.4428427 | 0.0007379 | 0.0021731 | -1.23809 |
| CD1C | -1.4098205 | 4.683796691 | -3.5508556 | 0.0005069 | 0.0015569 | -0.88897 |
| CD1A | -1.4086088 | 2.069944606 | -5.3183559 | 3.55E-07 | 2.41E-06 | 6.021933 |
| NR4A2 | -1.4054176 | 9.190141982 | -4.7957056 | 3.73E-06 | 1.97E-05 | 3.759807 |
| PRR16 | -1.4040531 | 3.291447414 | -3.3059189 | 0.001173 | 0.003267 | -1.66731 |
| CMKLR1 | -1.4032107 | 6.242105401 | -4.5589416 | 1.03E-05 | 4.90E-05 | 2.79198 |
| PFKFB4 | -1.4025204 | 8.047969721 | -7.899909 | 4.55E-13 | 1.32E-11 | 19.21902 |
| SIGLEC10 | -1.401509 | 8.847069967 | -7.110336 | 3.83E-11 | 7.00E-10 | 14.88656 |
| MT1G | -1.4008298 | 1.428831172 | -4.6270006 | 7.71E-06 | 3.78E-05 | 3.066397 |
| P2RY6 | -1.3987812 | 4.27589206 | -5.4038123 | 2.38E-07 | 1.68E-06 | 6.407403 |
| ZNF532 | -1.389015 | 6.766860253 | -4.3580703 | 2.36E-05 | 0.000101 | 2.000338 |
| PLA2G2F | -1.3880612 | 1.513016733 | -5.2300273 | 5.34E-07 | 3.49E-06 | 5.627984 |
| TRIB1 | -1.3866395 | 10.52331822 | -8.250137 | 5.98E-14 | 2.14E-12 | 21.20386 |
| IPCEF1 | -1.3853841 | 8.140524879 | -5.8485465 | 2.79E-08 | 2.46E-07 | 8.479425 |
| TBC1D8 | -1.384152 | 8.587567539 | -8.8268456 | 1.98E-15 | 1.09E-13 | 24.54219 |
| OR52K1 | -1.3840293 | 1.75109254 | -6.0889155 | 8.39E-09 | 8.36E-08 | 9.642912 |
| MT1F | -1.3807204 | 4.35581831 | -5.3300103 | 3.36E-07 | 2.30E-06 | 6.074253 |
| RASGRP4 | -1.3750298 | 10.22351711 | -7.2267238 | 2.02E-11 | 3.95E-10 | 15.51147 |
| HBEGF | -1.3746111 | 7.843645653 | -5.1779083 | 6.78E-07 | 4.30E-06 | 5.3977 |
| OAS1 | -1.3712385 | 8.644047985 | -8.1429767 | 1.12E-13 | 3.78E-12 | 20.59287 |
| CTSB | -1.3686376 | 12.44447944 | -10.656812 | 2.68E-20 | 6.22E-18 | 35.54271 |
| ADAM9 | -1.3652549 | 8.702042019 | -6.4302442 | 1.46E-09 | 1.80E-08 | 11.34376 |
| CAMSAP1L1 | -1.3629382 | 5.282723359 | -6.1571045 | 5.94E-09 | 6.20E-08 | 9.978242 |
| ADCY9 | -1.3621103 | 8.947899163 | -5.5872176 | 9.94E-08 | 7.74E-07 | 7.248735 |
| LGI2 | -1.3602003 | 3.254489396 | -4.4198906 | 1.83E-05 | 8.11E-05 | 2.241031 |
| LDLRAD3 | -1.3601778 | 6.861480661 | -3.6423751 | 0.0003664 | 0.001168 | -0.586 |
| ITGB7 | -1.3599978 | 9.923578418 | -8.103656 | 1.40E-13 | 4.64E-12 | 20.36947 |
| RELL2 | -1.3575757 | 6.404463965 | -6.4651711 | 1.21E-09 | 1.53E-08 | 11.52087 |
| UNC93B1 | -1.3568831 | 9.919335242 | -10.081563 | 9.56E-19 | 1.42E-16 | 32.0347 |
| CPNE8 | -1.356839 | 8.042053251 | -2.7559381 | 0.0065446 | 0.0149444 | -3.23591 |
| ANG | -1.3557132 | 3.489130521 | -6.1915835 | 4.98E-09 | 5.32E-08 | 10.14866 |
| CLEC4D | -1.3557002 | 4.208137779 | -3.3424876 | 0.0010378 | 0.0029339 | -1.55415 |
| TCN2 | -1.3524479 | 8.008058048 | -5.4252697 | 2.15E-07 | 1.54E-06 | 6.504852 |
| AGTRAP | -1.3510021 | 9.429519607 | -8.4548852 | 1.80E-14 | 7.71E-13 | 22.37973 |
| PI4K2A | -1.3505611 | 7.754420134 | -7.133301 | 3.38E-11 | 6.23E-10 | 15.00946 |
| ENHO | -1.3487086 | 1.538256906 | -5.1808613 | 6.69E-07 | 4.25E-06 | 5.410704 |
| FAM82A1 | -1.348559 | 5.470734846 | -5.8677953 | 2.53E-08 | 2.26E-07 | 8.571509 |
| MGAM | -1.3463909 | 6.309604826 | -3.5994628 | 0.000427 | 0.0013377 | -0.72888 |
| C15orf38 | -1.3444455 | 3.915106009 | -3.819654 | 0.000192 | 0.0006562 | 0.019239 |
| SRGN | -1.3421602 | 14.38198813 | -5.8727094 | 2.47E-08 | 2.21E-07 | 8.595048 |
| C1orf38 | -1.3420264 | 10.85975096 | -9.8809261 | 3.30E-18 | 4.05E-16 | 30.82002 |
| NMUR1 | -1.3418103 | 5.676215544 | -4.6601052 | 6.70E-06 | 3.34E-05 | 3.200991 |
| TLR2 | -1.3408705 | 10.90090235 | -6.3536314 | 2.17E-09 | 2.56E-08 | 10.95722 |
| RAB34 | -1.3394289 | 8.689157128 | -3.8976734 | 0.0001435 | 0.0005079 | 0.293158 |
| ZFP36L1 | -1.3393895 | 10.01892825 | -6.1261945 | 6.95E-09 | 7.11E-08 | 9.825955 |
| MAP3K6 | -1.337847 | 7.481616241 | -6.8105061 | 1.95E-10 | 2.98E-09 | 13.30091 |
| LPL | -1.3370849 | 5.367852422 | -3.4946764 | 0.0006169 | 0.0018522 | -1.07171 |
| CD1E | -1.3362564 | 2.480277679 | -3.2856322 | 0.0012549 | 0.0034654 | -1.72961 |
| SRGAP1 | -1.3359039 | 6.15745818 | -4.8277748 | 3.25E-06 | 1.74E-05 | 3.89371 |
| MPP7 | -1.334278 | 8.029316312 | -5.3717008 | 2.76E-07 | 1.93E-06 | 6.262061 |
| P2RY2 | -1.3323196 | 7.915734857 | -6.1183528 | 7.23E-09 | 7.35E-08 | 9.787394 |
| MEGF9 | -1.3316745 | 10.84201024 | -8.147041 | 1.09E-13 | 3.70E-12 | 20.61598 |
| SERPINB8 | -1.3314947 | 9.908296781 | -5.9483955 | 1.70E-08 | 1.58E-07 | 8.959168 |
| SEMA4A | -1.3306779 | 10.12927103 | -6.4823619 | 1.11E-09 | 1.41E-08 | 11.60824 |
| ACSL1 | -1.3290339 | 10.70792175 | -6.9415304 | 9.62E-11 | 1.59E-09 | 13.98943 |
| GPR109A | -1.3279444 | 6.105989191 | -6.2078388 | 4.59E-09 | 4.95E-08 | 10.2292 |
| GPR65 | -1.3277629 | 7.502875161 | -6.5468328 | 7.91E-10 | 1.04E-08 | 11.9371 |
| CD180 | -1.3273751 | 10.05722983 | -5.5291884 | 1.31E-07 | 9.92E-07 | 6.980497 |
| PDE2A | -1.3268943 | 3.542424887 | -4.6698877 | 6.42E-06 | 3.21E-05 | 3.240902 |
| SAGE1 | -1.3263656 | 1.862094784 | -2.7069596 | 0.0075415 | 0.0169581 | -3.36318 |
| ADAM8 | -1.3243018 | 9.855234324 | -5.6823506 | 6.28E-08 | 5.10E-07 | 7.692496 |
| UBXN11 | -1.3232784 | 9.878367933 | -10.268249 | 3.01E-19 | 4.83E-17 | 33.16934 |
| H2AFJ | -1.3228097 | 7.516283407 | -5.687412 | 6.13E-08 | 4.99E-07 | 7.716243 |
| PADI4 | -1.322751 | 8.707504396 | -5.2250636 | 5.46E-07 | 3.56E-06 | 5.605983 |
| CLEC3B | -1.3200715 | 3.310769418 | -5.4716315 | 1.73E-07 | 1.27E-06 | 6.7163 |
| MICAL2 | -1.3190643 | 9.673271166 | -7.3072646 | 1.29E-11 | 2.65E-10 | 15.94685 |
| CES8 | -1.3099078 | 4.549334763 | -4.291702 | 3.09E-05 | 0.0001286 | 1.744895 |
| MOSC2 | -1.3095986 | 3.897296464 | -3.1631673 | 0.0018737 | 0.0049679 | -2.09863 |
| SLC22A18AS | -1.3095676 | 2.542176898 | -7.501833 | 4.36E-12 | 9.87E-11 | 17.00818 |
| APCDD1 | -1.3055392 | 3.713988594 | -3.4937073 | 0.000619 | 0.0018573 | -1.07484 |
| HFE | -1.3027966 | 3.800299406 | -5.4187529 | 2.22E-07 | 1.59E-06 | 6.475228 |
| FAM127B | -1.302694 | 6.712193003 | -4.235034 | 3.88E-05 | 0.0001574 | 1.529235 |
| SLC9A9 | -1.3022362 | 6.433872067 | -5.4643374 | 1.79E-07 | 1.31E-06 | 6.682952 |
| EEF1DP3 | -1.3022232 | 3.013853607 | -5.681931 | 6.30E-08 | 5.11E-07 | 7.690528 |
| HOXA10 | -1.3017408 | 8.181804293 | -2.6883804 | 0.0079542 | 0.0177705 | -3.41092 |
| ZFP36 | -1.2994262 | 12.18144601 | -8.4843636 | 1.51E-14 | 6.66E-13 | 22.54991 |
| CARD9 | -1.2974458 | 8.771069389 | -5.3515504 | 3.04E-07 | 2.10E-06 | 6.171162 |
| C10orf41 | -1.297154 | 2.82395073 | -5.8930684 | 2.24E-08 | 2.02E-07 | 8.692703 |
| NUDT16P1 | -1.2956965 | 5.168545885 | -3.8947725 | 0.0001451 | 0.0005123 | 0.282891 |
| SEPX1 | -1.2951697 | 8.906456529 | -10.005514 | 1.53E-18 | 2.02E-16 | 31.57368 |
| CAMK2D | -1.294345 | 6.994829479 | -4.8822993 | 2.56E-06 | 1.40E-05 | 4.122882 |
| TNFSF9 | -1.2943204 | 4.967520781 | -4.8945 | 2.42E-06 | 1.34E-05 | 4.174422 |
| PTGS2 | -1.2931546 | 8.767363913 | -4.0606922 | 7.70E-05 | 0.0002906 | 0.880108 |
| CRTAM | -1.2896189 | 4.717364236 | -6.0817529 | 8.70E-09 | 8.63E-08 | 9.607821 |
| NAGA | -1.2817606 | 10.61978435 | -11.032282 | 2.56E-21 | 9.04E-19 | 37.84832 |
| TMEM170B | -1.2813956 | 10.65137215 | -5.9680886 | 1.54E-08 | 1.45E-07 | 9.054391 |
| LPPR2 | -1.2797781 | 8.966105404 | -10.333052 | 2.01E-19 | 3.66E-17 | 33.56412 |
| KCNJ15 | -1.2797542 | 3.413169521 | -3.9130127 | 0.0001354 | 0.0004826 | 0.347549 |
| SAT1 | -1.2775784 | 11.39636604 | -8.4829421 | 1.53E-14 | 6.70E-13 | 22.5417 |
| JAZF1 | -1.2775124 | 8.146510449 | -6.8649629 | 1.45E-10 | 2.30E-09 | 13.58622 |
| VSTM1 | -1.2774547 | 5.671239905 | -2.7060837 | 0.0075605 | 0.0169962 | -3.36544 |
| CADM1 | -1.2768448 | 4.056512309 | -3.9601383 | 0.0001133 | 0.0004105 | 0.515744 |
| ASB2 | -1.2765868 | 4.875117567 | -6.0776391 | 8.88E-09 | 8.78E-08 | 9.587679 |
| TIMP1 | -1.2737827 | 10.48635933 | -8.076682 | 1.64E-13 | 5.31E-12 | 20.21647 |
| PRKCD | -1.2723423 | 11.3135397 | -9.5997021 | 1.85E-17 | 1.78E-15 | 29.12677 |
| PPARD | -1.2698796 | 7.991070205 | -8.6770788 | 4.84E-15 | 2.35E-13 | 23.66762 |
| NAMPT | -1.2681972 | 10.60489619 | -6.9563147 | 8.88E-11 | 1.47E-09 | 14.06755 |
| KLHL30 | -1.2637989 | 1.640028837 | -4.9013231 | 2.35E-06 | 1.30E-05 | 4.203286 |
| BATF2 | -1.262853 | 4.050696282 | -5.7541752 | 4.43E-08 | 3.74E-07 | 8.03078 |
| TNFRSF11A | -1.2623054 | 4.390853685 | -3.8558908 | 0.0001678 | 0.0005827 | 0.145894 |
| AATK | -1.2604017 | 6.836864801 | -5.6684408 | 6.72E-08 | 5.41E-07 | 7.627304 |
| S100A16 | -1.260198 | 3.546473074 | -2.7311926 | 0.0070322 | 0.0159412 | -3.30047 |
| TCIRG1 | -1.2601353 | 11.51174526 | -9.5088294 | 3.22E-17 | 2.83E-15 | 28.58219 |
| GAA | -1.2601256 | 10.23817091 | -7.6881786 | 1.52E-12 | 3.86E-11 | 18.03677 |
| FN1 | -1.2600174 | 7.80848501 | -4.3832689 | 2.13E-05 | 9.23E-05 | 2.098128 |
| TUSC1 | -1.2599926 | 3.35225451 | -3.0293441 | 0.0028662 | 0.0072555 | -2.48774 |
| TSPO | -1.2591426 | 10.00605409 | -8.3133171 | 4.14E-14 | 1.58E-12 | 21.56554 |
| CXCR1 | -1.257973 | 4.201763235 | -4.5878936 | 9.10E-06 | 4.39E-05 | 2.908337 |
| EREG | -1.257829 | 7.954928052 | -2.4894368 | 0.0138359 | 0.0289605 | -3.90318 |
| SPR | -1.2565121 | 4.4563042 | -5.4193091 | 2.21E-07 | 1.58E-06 | 6.477755 |
| SLC22A18 | -1.2551889 | 6.676342992 | -7.1207657 | 3.62E-11 | 6.64E-10 | 14.94235 |
| FAM114A1 | -1.2544393 | 4.665059836 | -5.6396938 | 7.72E-08 | 6.14E-07 | 7.492906 |
| MMP9 | -1.2516343 | 4.099736048 | -2.8741248 | 0.0046128 | 0.0110282 | -2.92029 |
| IRS2 | -1.2514818 | 10.7407566 | -7.1411432 | 3.24E-11 | 6.01E-10 | 15.05147 |
| JOSD2 | -1.250977 | 5.752175574 | -8.3606403 | 3.13E-14 | 1.25E-12 | 21.83713 |
| KCNK10 | -1.2471473 | 1.986406338 | -3.2797855 | 0.0012795 | 0.0035231 | -1.74751 |
| CAPG | -1.2470214 | 10.04615057 | -5.2694296 | 4.45E-07 | 2.95E-06 | 5.803152 |
| SLC16A3 | -1.2451432 | 9.630370221 | -8.2927934 | 4.66E-14 | 1.75E-12 | 21.44793 |
| MSLN | -1.2445906 | 2.587881564 | -2.365576 | 0.0192221 | 0.0386155 | -4.192 |
| C17orf77 | -1.2437199 | 1.012372119 | -6.9966785 | 7.13E-11 | 1.21E-09 | 14.28129 |
| AGT | -1.2430867 | 2.175276609 | -3.7665271 | 0.0002336 | 0.0007843 | -0.16466 |
| WNT5B | -1.2426274 | 3.698672953 | -5.4078996 | 2.33E-07 | 1.66E-06 | 6.425945 |
| MMP8 | -1.2398664 | 5.512431037 | -2.4367201 | 0.0159378 | 0.0327518 | -4.02778 |
| CRIP1 | -1.2385675 | 8.302481951 | -4.6626421 | 6.63E-06 | 3.31E-05 | 3.211335 |
| C10orf105 | -1.2377537 | 6.05638655 | -5.7188759 | 5.26E-08 | 4.36E-07 | 7.864179 |
| CLDN23 | -1.235916 | 3.548079295 | -5.2070155 | 5.93E-07 | 3.83E-06 | 5.526108 |
| HOMER3 | -1.2355524 | 8.991766456 | -4.3219395 | 2.73E-05 | 0.0001152 | 1.860894 |
| DKK2 | -1.2343127 | 3.088685794 | -3.1150611 | 0.0021865 | 0.0057012 | -2.24022 |
| SLC4A3 | -1.2331229 | 3.580878085 | -3.2904158 | 0.0012351 | 0.0034178 | -1.71495 |
| MT1X | -1.2325769 | 5.146659921 | -6.0827065 | 8.66E-09 | 8.60E-08 | 9.612492 |
| ABLIM3 | -1.2317085 | 3.408394731 | -3.9019948 | 0.0001412 | 0.0005006 | 0.308463 |
| FCGR3B | -1.2295729 | 4.600946985 | -3.0240407 | 0.002914 | 0.0073657 | -2.50286 |
| HLA-DRB1 | -1.2279522 | 10.93349212 | -4.6875913 | 5.96E-06 | 3.00E-05 | 3.313291 |
| GPR132 | -1.2276002 | 8.532835228 | -8.3980415 | 2.52E-14 | 1.03E-12 | 22.05219 |
| FCGBP | -1.2274272 | 5.820659123 | -4.1694406 | 5.03E-05 | 0.0001991 | 1.28245 |
| CDC42EP3 | -1.2271562 | 10.21142983 | -7.9631787 | 3.16E-13 | 9.55E-12 | 19.57497 |
| KRT80 | -1.2258336 | 2.895643219 | -4.2198956 | 4.12E-05 | 0.0001663 | 1.472007 |
| APOA2 | -1.2252688 | 1.22291331 | -7.9428365 | 3.55E-13 | 1.05E-11 | 19.4604 |
| CCDC149 | -1.2244905 | 7.065397074 | -4.3057316 | 2.92E-05 | 0.0001222 | 1.798636 |
| BLVRA | -1.2240914 | 7.395118123 | -4.4661796 | 1.51E-05 | 6.89E-05 | 2.422976 |
| ARSG | -1.2235185 | 5.462182602 | -6.0037522 | 1.29E-08 | 1.24E-07 | 9.227339 |
| CTSD | -1.2230274 | 13.08170226 | -8.2406152 | 6.33E-14 | 2.25E-12 | 21.14944 |
| DYSFIP1 | -1.2210529 | 4.623266388 | -3.3646058 | 0.0009633 | 0.0027489 | -1.48518 |
| CTSG | -1.2202804 | 9.726410217 | -2.6347553 | 0.0092626 | 0.0203435 | -3.54702 |
| GNA14 | -1.2135232 | 3.482656092 | -4.7110405 | 5.39E-06 | 2.74E-05 | 3.40949 |
| PECR | -1.2128143 | 4.363906537 | -3.9800495 | 0.000105 | 0.000384 | 0.587304 |
| EMP3 | -1.210765 | 10.62128627 | -8.8941732 | 1.33E-15 | 7.76E-14 | 24.93695 |
| GPR88 | -1.2092136 | 3.823345618 | -4.5640743 | 1.01E-05 | 4.81E-05 | 2.812567 |
| LOC388387 | -1.2070565 | 2.451886438 | -4.3964405 | 2.02E-05 | 8.81E-05 | 2.149419 |
| PHLDA2 | -1.2065801 | 2.335409586 | -5.7053022 | 5.62E-08 | 4.62E-07 | 7.800294 |
| SHISA4 | -1.2055149 | 2.835031568 | -4.9338169 | 2.04E-06 | 1.14E-05 | 4.341149 |
| STARD8 | -1.2053005 | 8.261111837 | -4.4642653 | 1.53E-05 | 6.93E-05 | 2.415422 |
| IL4R | -1.2052967 | 9.877210877 | -9.6359568 | 1.48E-17 | 1.44E-15 | 29.3444 |
| TRIM36 | -1.205153 | 3.74500715 | -4.7811738 | 3.98E-06 | 2.09E-05 | 3.699349 |
| ME1 | -1.2046499 | 4.392975574 | -3.268412 | 0.0013286 | 0.0036416 | -1.78224 |
| KIF17 | -1.2041108 | 4.771402843 | -2.778597 | 0.0061252 | 0.0141219 | -3.17633 |
| GDF15 | -1.2038511 | 3.282683546 | -4.1518968 | 5.39E-05 | 0.0002115 | 1.216965 |
| LPHN2 | -1.2022357 | 3.219243618 | -3.5333123 | 0.0005391 | 0.0016432 | -0.9463 |
| LILRA4 | -1.2021872 | 3.906946729 | -3.4445172 | 0.0007336 | 0.0021629 | -1.23275 |
| MVP | -1.2010617 | 10.28671125 | -10.068023 | 1.04E-18 | 1.52E-16 | 31.95257 |
| TP53INP2 | -1.1991091 | 8.981644007 | -4.6105428 | 8.27E-06 | 4.02E-05 | 2.999755 |
| CACNA2D4 | -1.1965896 | 8.486717184 | -4.55053 | 1.07E-05 | 5.05E-05 | 2.758279 |
| MYO1E | -1.1963435 | 6.279913325 | -4.6732195 | 6.33E-06 | 3.17E-05 | 3.25451 |
| TBC1D2 | -1.1953985 | 9.319113409 | -10.241215 | 3.56E-19 | 5.61E-17 | 33.00479 |
| COLEC12 | -1.1944254 | 2.981489969 | -3.6434417 | 0.000365 | 0.0011638 | -0.58244 |
| GALM | -1.1936372 | 6.401222995 | -5.5609775 | 1.13E-07 | 8.65E-07 | 7.127209 |
| SH3TC1 | -1.1928093 | 9.272039338 | -5.6872664 | 6.13E-08 | 4.99E-07 | 7.71556 |
| PLIN5 | -1.1926556 | 4.349668246 | -5.6433878 | 7.59E-08 | 6.04E-07 | 7.510151 |
| BMP2 | -1.1919035 | 2.513683008 | -3.6189676 | 0.0003983 | 0.001256 | -0.66411 |
| TKTL2 | -1.1906477 | 1.635644345 | -6.4614694 | 1.24E-09 | 1.55E-08 | 11.50207 |
| AP1S2 | -1.1885362 | 10.0835974 | -8.599821 | 7.65E-15 | 3.54E-13 | 23.21848 |
| DUSP6 | -1.1868059 | 11.63029344 | -6.0901702 | 8.34E-09 | 8.32E-08 | 9.649061 |
| TACC2 | -1.1865335 | 1.453199994 | -4.3588141 | 2.35E-05 | 0.0001007 | 2.003219 |
| CYB5R2 | -1.1858121 | 1.641323026 | -4.1689018 | 5.04E-05 | 0.0001993 | 1.280436 |
| MANSC1 | -1.1850474 | 6.642466736 | -4.855077 | 2.88E-06 | 1.56E-05 | 4.008227 |
| PLA2G2D | -1.183022 | 2.49596421 | -5.1305468 | 8.41E-07 | 5.21E-06 | 5.189848 |
| SLC2A3 | -1.1828072 | 11.05791563 | -6.2135189 | 4.45E-09 | 4.82E-08 | 10.25738 |
| RAB24 | -1.1822996 | 8.669465916 | -9.5648992 | 2.29E-17 | 2.12E-15 | 28.91805 |
| TAGLN | -1.1799201 | 7.482911429 | -8.6364956 | 6.16E-15 | 2.91E-13 | 23.43152 |
| CAST | -1.1794189 | 11.11072245 | -7.2061273 | 2.26E-11 | 4.36E-10 | 15.40051 |
| HLA-DRB5 | -1.1792896 | 9.274314214 | -3.4804183 | 0.0006482 | 0.0019324 | -1.11769 |
| GGT1 | -1.1782148 | 7.411262233 | -4.3734034 | 2.22E-05 | 9.58E-05 | 2.05979 |
| FAM49A | -1.1780252 | 7.827951221 | -5.4766377 | 1.69E-07 | 1.24E-06 | 6.739205 |
| CCIN | -1.1768915 | 3.964396489 | -5.343945 | 3.15E-07 | 2.17E-06 | 6.136914 |
| THBD | -1.1737852 | 6.581568102 | -5.0438853 | 1.25E-06 | 7.37E-06 | 4.813049 |
| SIGLEC5 | -1.1698692 | 8.643712543 | -6.8606742 | 1.49E-10 | 2.34E-09 | 13.56371 |
| CLC | -1.1698352 | 6.371553863 | -2.3429318 | 0.020386 | 0.0406367 | -4.24332 |
| EMILIN2 | -1.1673838 | 11.08517159 | -6.8685735 | 1.43E-10 | 2.26E-09 | 13.60518 |
| ANKRD57 | -1.1657667 | 2.42566402 | -4.4455814 | 1.65E-05 | 7.40E-05 | 2.341831 |
| IL21R | -1.164049 | 5.878125412 | -5.6622326 | 6.93E-08 | 5.56E-07 | 7.598241 |
| PHOSPHO1 | -1.1632565 | 5.57950991 | -4.435957 | 1.72E-05 | 7.66E-05 | 2.304016 |
| PPM1M | -1.1630899 | 9.111002813 | -9.816428 | 4.90E-18 | 5.52E-16 | 30.43067 |
| LPAR1 | -1.162159 | 5.192152669 | -3.5364258 | 0.0005333 | 0.0016278 | -0.93615 |
| DFNA5 | -1.1620951 | 4.344024473 | -3.9587675 | 0.0001139 | 0.0004125 | 0.510829 |
| TNFSF15 | -1.1616936 | 2.252570512 | -5.6175606 | 8.59E-08 | 6.75E-07 | 7.389736 |
| C4orf48 | -1.1611782 | 3.93936247 | -5.3781548 | 2.68E-07 | 1.88E-06 | 6.291226 |
| MTSS1 | -1.1609761 | 9.388054328 | -5.5373607 | 1.26E-07 | 9.57E-07 | 7.01816 |
| STAC3 | -1.1594033 | 8.022327292 | -6.2225467 | 4.25E-09 | 4.62E-08 | 10.30219 |
| C7orf58 | -1.1564105 | 4.887012354 | -2.8746603 | 0.0046053 | 0.0110154 | -2.91883 |
| FAM105A | -1.1563016 | 8.598381625 | -3.7090354 | 0.0002881 | 0.0009464 | -0.36124 |
| MMEL1 | -1.1555144 | 1.665620349 | -4.9006633 | 2.36E-06 | 1.30E-05 | 4.200494 |
| BLVRB | -1.1524983 | 9.105896701 | -6.1019217 | 7.86E-09 | 7.92E-08 | 9.706696 |
| SPATS2L | -1.1511225 | 6.652936354 | -3.9856101 | 0.0001028 | 0.0003765 | 0.60734 |
| VAV2 | -1.1484213 | 8.429922713 | -5.9700607 | 1.52E-08 | 1.44E-07 | 9.063938 |
| LOC152225 | -1.1477788 | 1.345624139 | -4.5989761 | 8.68E-06 | 4.21E-05 | 2.953027 |
| ABCA9 | -1.146085 | 4.42517422 | -3.6581551 | 0.0003462 | 0.0011096 | -0.53311 |
| ITGAL | -1.1453358 | 12.32382441 | -7.611284 | 2.36E-12 | 5.68E-11 | 17.61093 |
| CASP1 | -1.1429343 | 10.14142184 | -8.1649699 | 9.83E-14 | 3.36E-12 | 20.71801 |
| CLU | -1.1418511 | 9.17428344 | -3.070994 | 0.0025147 | 0.0064636 | -2.36824 |
| EMILIN1 | -1.1399966 | 7.432779125 | -3.2486501 | 0.0014181 | 0.0038608 | -1.84234 |
| SLC27A3 | -1.1395562 | 8.332535981 | -9.6417548 | 1.43E-17 | 1.40E-15 | 29.37923 |
| HEXB | -1.1392281 | 10.28363678 | -9.1287845 | 3.23E-16 | 2.14E-14 | 26.31991 |
| ANXA8 | -1.1382212 | 2.713176573 | -3.5103918 | 0.0005841 | 0.0017623 | -1.02084 |
| CLCN5 | -1.1375203 | 8.370059143 | -5.9401045 | 1.77E-08 | 1.64E-07 | 8.919137 |
| ATOH8 | -1.1372431 | 2.78932369 | -4.8868687 | 2.51E-06 | 1.38E-05 | 4.142174 |
| C2orf62 | -1.1350414 | 1.442651155 | -6.6460403 | 4.69E-10 | 6.58E-09 | 12.44673 |
| TXNDC3 | -1.134623 | 5.074278023 | -4.700456 | 5.64E-06 | 2.86E-05 | 3.366023 |
| CT45A3 | -1.1345766 | 1.410892747 | -2.6397542 | 0.009133 | 0.020094 | -3.53444 |
| ZCCHC24 | -1.1342925 | 7.339387093 | -5.661554 | 6.95E-08 | 5.57E-07 | 7.595066 |
| OLFML2A | -1.1338691 | 6.688078088 | -2.8743588 | 0.0046095 | 0.0110221 | -2.91966 |
| LOC285780 | -1.1333226 | 2.720501445 | -4.7716033 | 4.15E-06 | 2.17E-05 | 3.659607 |
| PGA3 | -1.1328513 | 1.150384253 | -4.5679344 | 9.90E-06 | 4.74E-05 | 2.828061 |
| LOC389634 | -1.1319612 | 3.214258618 | -4.595028 | 8.83E-06 | 4.27E-05 | 2.937097 |
| LOC100133161 | -1.1304805 | 6.723891467 | -7.4340424 | 6.38E-12 | 1.39E-10 | 16.63689 |
| NOS3 | -1.1302031 | 3.759067567 | -4.930568 | 2.07E-06 | 1.16E-05 | 4.327335 |
| TLE1 | -1.1296463 | 6.876236236 | -5.1463557 | 7.83E-07 | 4.88E-06 | 5.259078 |
| CITED4 | -1.1285627 | 7.729318607 | -4.7107615 | 5.39E-06 | 2.75E-05 | 3.408343 |
| CDKN2D | -1.1275495 | 8.271355556 | -8.5794242 | 8.64E-15 | 3.95E-13 | 23.10013 |
| TPST1 | -1.1273389 | 5.359284142 | -4.0226014 | 8.92E-05 | 0.0003307 | 0.74121 |
| ATP6V0A1 | -1.1241514 | 10.05030739 | -6.322247 | 2.55E-09 | 2.95E-08 | 10.79965 |
| CST6 | -1.1238922 | 2.248878997 | -6.7140337 | 3.27E-10 | 4.73E-09 | 12.79848 |
| SLC31A2 | -1.1236729 | 8.814954355 | -8.8673961 | 1.56E-15 | 8.83E-14 | 24.77983 |
| C1orf161 | -1.1234721 | 1.53144542 | -4.6151899 | 8.11E-06 | 3.95E-05 | 3.018554 |
| BEST1 | -1.1226229 | 7.052630025 | -6.7459026 | 2.75E-10 | 4.05E-09 | 12.96402 |
| HPD | -1.1224046 | 2.042233779 | -4.5797875 | 9.42E-06 | 4.53E-05 | 2.875703 |
| TGM2 | -1.122026 | 6.632896099 | -3.6072427 | 0.0004153 | 0.0013051 | -0.70308 |
| MPZL2 | -1.1216996 | 4.262796167 | -3.3537514 | 0.0009992 | 0.0028371 | -1.51908 |
| ALDH2 | -1.1209672 | 8.218368512 | -3.41405 | 0.0008144 | 0.0023742 | -1.3296 |
| MARCKS | -1.1206612 | 8.431129661 | -4.0946323 | 6.75E-05 | 0.0002588 | 1.004759 |
| UNC119 | -1.1205951 | 8.486081015 | -10.700354 | 2.04E-20 | 4.88E-18 | 35.80953 |
| PLD3 | -1.1203033 | 11.02112487 | -7.4245407 | 6.73E-12 | 1.46E-10 | 16.58498 |
| PLXDC2 | -1.1189122 | 8.872547157 | -4.4925315 | 1.36E-05 | 6.23E-05 | 2.52721 |
| PSRC1 | -1.1173189 | 6.564310468 | -6.0019114 | 1.30E-08 | 1.25E-07 | 9.218396 |
| ZBP1 | -1.1151331 | 5.227439457 | -5.1163075 | 8.98E-07 | 5.51E-06 | 5.127623 |
| SYTL3 | -1.1145661 | 6.780200372 | -5.412794 | 2.28E-07 | 1.63E-06 | 6.448161 |
| TMCC3 | -1.1138593 | 5.636469844 | -5.0066099 | 1.47E-06 | 8.52E-06 | 4.652394 |
| TOX2 | -1.1134811 | 2.978423286 | -4.151987 | 5.39E-05 | 0.0002115 | 1.217302 |
| CSPG4 | -1.1111173 | 5.698681995 | -4.0331996 | 8.56E-05 | 0.0003193 | 0.77975 |
| HLA-F | -1.1093042 | 9.154021822 | -5.8923727 | 2.24E-08 | 2.03E-07 | 8.689363 |
| C1orf115 | -1.1083147 | 3.413866983 | -4.8283237 | 3.24E-06 | 1.74E-05 | 3.896007 |
| SLC26A6 | -1.1080922 | 8.418984681 | -9.0737968 | 4.51E-16 | 2.90E-14 | 25.99478 |
| EVI5 | -1.1075358 | 8.533154164 | -7.0540087 | 5.22E-11 | 9.22E-10 | 14.58597 |
| HLA-DMB | -1.1074908 | 10.14635027 | -4.3044686 | 2.93E-05 | 0.0001228 | 1.793793 |
| RGL1 | -1.1059512 | 5.127415673 | -3.8245255 | 0.0001886 | 0.0006456 | 0.036208 |
| NLRP3 | -1.1035157 | 10.00893741 | -8.0138188 | 2.36E-13 | 7.33E-12 | 19.86072 |
| C1orf106 | -1.1034058 | 4.617419641 | -3.0571662 | 0.0026267 | 0.0067164 | -2.40807 |
| GFRA2 | -1.1030601 | 3.434899604 | -4.3571774 | 2.37E-05 | 0.0001013 | 1.996881 |
| RNASE4 | -1.1027218 | 4.933387702 | -4.5300775 | 1.16E-05 | 5.44E-05 | 2.676536 |
| PNOC | -1.1025847 | 4.113842436 | -5.4436399 | 1.97E-07 | 1.43E-06 | 6.588489 |
| GLDN | -1.1019276 | 3.957503831 | -3.0222381 | 0.0029305 | 0.007401 | -2.50799 |
| FTL | -1.1013699 | 14.04535242 | -10.316902 | 2.23E-19 | 3.80E-17 | 33.46569 |
| OR7E91P | -1.0985276 | 1.51731269 | -4.6957565 | 5.75E-06 | 2.91E-05 | 3.346748 |
| TPRG1 | -1.0976513 | 2.270443914 | -4.7689496 | 4.19E-06 | 2.19E-05 | 3.648597 |
| BVES | -1.0974047 | 2.053599256 | -3.3666036 | 0.0009568 | 0.0027329 | -1.47893 |
| SLAMF1 | -1.094689 | 5.714886445 | -5.1920281 | 6.35E-07 | 4.07E-06 | 5.459927 |
| HAL | -1.09466 | 8.375623675 | -4.0237676 | 8.88E-05 | 0.0003297 | 0.745447 |
| ECHDC3 | -1.094158 | 5.003317135 | -4.3593192 | 2.35E-05 | 0.0001006 | 2.005175 |
| ACE | -1.0940313 | 5.403907461 | -3.1681617 | 0.0018437 | 0.0048941 | -2.08382 |
| APOB48R | -1.0937285 | 10.35774833 | -7.3819587 | 8.54E-12 | 1.82E-10 | 16.35272 |
| GAS2L3 | -1.0915986 | 5.318702095 | -3.7437252 | 0.0002539 | 0.0008442 | -0.24293 |
| C9orf72 | -1.090883 | 9.746384915 | -7.1996635 | 2.35E-11 | 4.49E-10 | 15.36572 |
| WNT6 | -1.0907969 | 2.694301374 | -4.2303492 | 3.95E-05 | 0.0001601 | 1.511507 |
| C21orf67 | -1.0894099 | 5.306101336 | -7.5157941 | 4.03E-12 | 9.28E-11 | 17.08484 |
| GPRIN1 | -1.0889147 | 3.995376764 | -5.4192515 | 2.21E-07 | 1.58E-06 | 6.477493 |
| CD70 | -1.0883443 | 3.205293647 | -3.2446169 | 0.001437 | 0.0039034 | -1.85457 |
| TSKS | -1.0861773 | 2.639187683 | -3.2172305 | 0.001572 | 0.0042337 | -1.93724 |
| SLC12A9 | -1.0844996 | 10.13674506 | -8.8052827 | 2.26E-15 | 1.22E-13 | 24.41596 |
| HLA-DQA1 | -1.0842947 | 9.251075793 | -3.2729785 | 0.0013086 | 0.003593 | -1.76831 |
| MCOLN2 | -1.0837647 | 4.294624089 | -3.7592454 | 0.0002399 | 0.0008027 | -0.1897 |
| BCL2L11 | -1.0828055 | 8.803061513 | -6.8427449 | 1.64E-10 | 2.55E-09 | 13.46967 |
| CARD16 | -1.0822246 | 7.951768358 | -7.3371054 | 1.10E-11 | 2.28E-10 | 16.10876 |
| MAP1LC3A | -1.0800492 | 4.356793667 | -4.4808771 | 1.42E-05 | 6.51E-05 | 2.481053 |
| MICAL1 | -1.0782332 | 10.91654126 | -6.3327524 | 2.41E-09 | 2.82E-08 | 10.85234 |
| KNDC1 | -1.0780127 | 2.038481221 | -4.3736457 | 2.22E-05 | 9.57E-05 | 2.060731 |
| PTK2 | -1.0775951 | 6.791690425 | -2.5998301 | 0.010216 | 0.0221674 | -3.63431 |
| KCTD17 | -1.0761532 | 5.967565313 | -5.5869973 | 9.95E-08 | 7.75E-07 | 7.247713 |
| GGTLC2 | -1.0748153 | 3.80946484 | -4.3202176 | 2.75E-05 | 0.000116 | 1.854271 |
| TNFSF13 | -1.0746194 | 9.742817756 | -7.6739923 | 1.65E-12 | 4.13E-11 | 17.95806 |
| TNFSF14 | -1.0744627 | 3.820466556 | -4.9990347 | 1.52E-06 | 8.79E-06 | 4.619851 |
| SERPINF1 | -1.0741163 | 6.724177631 | -3.8360109 | 0.0001807 | 0.0006224 | 0.076287 |
| PLB1 | -1.06806 | 8.610274821 | -5.7599211 | 4.31E-08 | 3.65E-07 | 8.057962 |
| TMEM169 | -1.0677141 | 2.510578049 | -4.4522647 | 1.60E-05 | 7.22E-05 | 2.368128 |
| TRNP1 | -1.0676604 | 2.643756165 | -3.9085077 | 0.0001377 | 0.0004899 | 0.331557 |
| SRC | -1.0648919 | 8.208755331 | -4.7883452 | 3.86E-06 | 2.03E-05 | 3.729168 |
| GCNT2 | -1.0645488 | 7.625249132 | -5.0398143 | 1.27E-06 | 7.47E-06 | 4.795461 |
| IQSEC1 | -1.0634126 | 10.74628768 | -7.5261896 | 3.81E-12 | 8.79E-11 | 17.14196 |
| LDLR | -1.0628962 | 9.295732112 | -5.5464603 | 1.21E-07 | 9.22E-07 | 7.06014 |
| SPINT1 | -1.0615532 | 7.861327609 | -4.1759239 | 4.90E-05 | 0.0001946 | 1.306706 |
| RETN | -1.0593208 | 3.837733804 | -2.3202318 | 0.0216146 | 0.042734 | -4.29431 |
| TMEM120A | -1.0590508 | 7.315820415 | -8.8748861 | 1.49E-15 | 8.59E-14 | 24.82377 |
| 44084 | -1.0579329 | 3.348542886 | -3.9087076 | 0.0001376 | 0.0004896 | 0.332266 |
| ZNF467 | -1.0572736 | 8.640120734 | -5.310834 | 3.67E-07 | 2.49E-06 | 5.988206 |
| PLCD3 | -1.0562309 | 6.304577866 | -5.1240296 | 8.67E-07 | 5.35E-06 | 5.161353 |
| IL4I1 | -1.0554401 | 5.768337767 | -7.3303715 | 1.14E-11 | 2.36E-10 | 16.07219 |
| TMTC2 | -1.0552003 | 8.265761078 | -3.7000261 | 0.0002977 | 0.0009743 | -0.39182 |
| C10orf11 | -1.0546562 | 6.143358829 | -3.926928 | 0.0001285 | 0.0004598 | 0.397042 |
| TNS3 | -1.0540797 | 10.96888728 | -5.6278788 | 8.18E-08 | 6.47E-07 | 7.437799 |
| KLF2 | -1.053648 | 10.36976547 | -5.4158954 | 2.25E-07 | 1.60E-06 | 6.462246 |
| EFHD2 | -1.0532081 | 11.48263433 | -10.068052 | 1.04E-18 | 1.52E-16 | 31.95274 |
| P2RX7 | -1.0530013 | 8.818023114 | -6.7597616 | 2.56E-10 | 3.78E-09 | 13.03614 |
| HLA-DRB6 | -1.0529194 | 7.347463358 | -2.9936298 | 0.0032031 | 0.0080072 | -2.58907 |
| SOX13 | -1.052494 | 5.096969604 | -3.6708785 | 0.0003307 | 0.0010671 | -0.49032 |
| SIDT2 | -1.0519307 | 10.19703086 | -8.8904454 | 1.36E-15 | 7.91E-14 | 24.91507 |
| IL27 | -1.0512646 | 1.727678112 | -6.2612025 | 3.49E-09 | 3.89E-08 | 10.49451 |
| BRI3 | -1.0497529 | 8.67492691 | -8.6911456 | 4.45E-15 | 2.18E-13 | 23.74955 |
| OSGIN1 | -1.0496848 | 3.532957258 | -5.470259 | 1.74E-07 | 1.27E-06 | 6.710022 |
| IL15 | -1.0484463 | 4.222277145 | -3.4147 | 0.0008126 | 0.0023697 | -1.32754 |
| FRAT1 | -1.0468725 | 8.362546808 | -9.1338771 | 3.14E-16 | 2.09E-14 | 26.35005 |
| PTX3 | -1.0461466 | 6.227226802 | -4.3595069 | 2.35E-05 | 0.0001005 | 2.005901 |
| LOC653653 | -1.0458289 | 7.528643416 | -8.0468801 | 1.95E-13 | 6.15E-12 | 20.04768 |
| DAAM2 | -1.0456345 | 3.343596551 | -2.7705321 | 0.0062716 | 0.0144047 | -3.19758 |
| CTTNBP2NL | -1.0456144 | 7.104297235 | -4.3978639 | 2.01E-05 | 8.77E-05 | 2.154969 |
| RASL11A | -1.0453123 | 4.403126364 | -5.3211534 | 3.50E-07 | 2.39E-06 | 6.034484 |
| RNF175 | -1.0452545 | 6.257935285 | -4.4201537 | 1.83E-05 | 8.11E-05 | 2.242061 |
| SH2B2 | -1.0450564 | 6.104855083 | -6.2421236 | 3.85E-09 | 4.23E-08 | 10.3995 |
| VAT1L | -1.0449376 | 2.103559262 | -2.6019359 | 0.0101561 | 0.0220756 | -3.62908 |
| CCDC109B | -1.0443678 | 7.738911731 | -6.2707044 | 3.32E-09 | 3.73E-08 | 10.54189 |
| JUNB | -1.0439903 | 12.03608339 | -6.2372164 | 3.95E-09 | 4.32E-08 | 10.37509 |
| ATP6V0D1 | -1.0435801 | 10.49338477 | -11.099735 | 1.68E-21 | 6.41E-19 | 38.26355 |
| SLC2A8 | -1.0435745 | 5.302394902 | -4.4188019 | 1.84E-05 | 8.14E-05 | 2.236769 |
| ITGB5 | -1.0430957 | 5.989432345 | -4.0246474 | 8.85E-05 | 0.0003288 | 0.748644 |
| PRKAR2B | -1.0420273 | 9.494638073 | -4.4686071 | 1.50E-05 | 6.82E-05 | 2.432558 |
| CCDC151 | -1.0409608 | 1.339000416 | -5.39959 | 2.42E-07 | 1.72E-06 | 6.388258 |
| IFI27L2 | -1.040514 | 5.767175204 | -5.4574208 | 1.85E-07 | 1.35E-06 | 6.651358 |
| LOC100302650 | -1.0402744 | 5.735197029 | -5.5641707 | 1.11E-07 | 8.54E-07 | 7.141978 |
| NETO2 | -1.0401956 | 8.130513663 | -3.7899376 | 0.0002143 | 0.0007256 | -0.08389 |
| SLC47A1 | -1.037768 | 2.484909466 | -3.9926918 | 0.0001 | 0.0003675 | 0.632891 |
| SLC36A1 | -1.0377236 | 10.01629024 | -7.2653886 | 1.63E-11 | 3.27E-10 | 15.72018 |
| CCDC88B | -1.03682 | 10.94615074 | -8.0123959 | 2.38E-13 | 7.37E-12 | 19.85268 |
| SLC12A8 | -1.0364768 | 2.75236663 | -5.3788495 | 2.67E-07 | 1.88E-06 | 6.294366 |
| CECR2 | -1.0351217 | 2.109810533 | -3.9023086 | 0.000141 | 0.0005003 | 0.309575 |
| MYO1F | -1.034837 | 12.36995125 | -10.437378 | 1.05E-19 | 2.18E-17 | 34.20058 |
| PELI1 | -1.0346162 | 9.840398055 | -5.2926881 | 3.99E-07 | 2.68E-06 | 5.906981 |
| NADK | -1.0334168 | 10.56542865 | -12.694153 | 7.32E-26 | 2.95E-22 | 48.12248 |
| STEAP4 | -1.0332466 | 5.032152197 | -3.1812528 | 0.0017672 | 0.0047142 | -2.04491 |
| RHBDF2 | -1.0321071 | 10.03980856 | -7.7207346 | 1.27E-12 | 3.28E-11 | 18.21764 |
| DKFZp761E198 | -1.0316344 | 10.6780293 | -7.2307252 | 1.98E-11 | 3.88E-10 | 15.53304 |
| IL22RA2 | -1.0316026 | 1.123670758 | -3.594734 | 0.0004342 | 0.001357 | -0.74453 |
| PPM1J | -1.0313762 | 2.785923022 | -3.1162037 | 0.0021785 | 0.005685 | -2.23688 |
| CBR1 | -1.0307869 | 8.056896705 | -5.6776499 | 6.43E-08 | 5.21E-07 | 7.670453 |
| SDC4 | -1.0307247 | 6.397521758 | -4.0599208 | 7.72E-05 | 0.0002911 | 0.877284 |
| RGL3 | -1.0304469 | 2.295638318 | -4.2041107 | 4.39E-05 | 0.0001756 | 1.412509 |
| OLR1 | -1.030426 | 4.004464853 | -3.155708 | 0.0019193 | 0.0050789 | -2.12071 |
| HRH1 | -1.029451 | 2.861523504 | -3.8343299 | 0.0001818 | 0.0006258 | 0.070415 |
| OTUD1 | -1.02929 | 8.483618744 | -6.2018662 | 4.73E-09 | 5.07E-08 | 10.1996 |
| MKX | -1.0287541 | 1.644556031 | -5.4892993 | 1.59E-07 | 1.17E-06 | 6.7972 |
| PTGIR | -1.0285843 | 6.526950381 | -3.3719126 | 0.0009398 | 0.0026933 | -1.46231 |
| RARA | -1.0285481 | 9.888222548 | -9.125 | 3.31E-16 | 2.18E-14 | 26.29751 |
| DUSP1 | -1.0280227 | 12.74452436 | -6.3221102 | 2.55E-09 | 2.95E-08 | 10.79897 |
| SCARA5 | -1.026433 | 1.151211352 | -4.1671755 | 5.08E-05 | 0.0002005 | 1.273983 |
| PELI3 | -1.0250514 | 6.339268533 | -6.8966909 | 1.23E-10 | 1.97E-09 | 13.75301 |
| ARHGAP21 | -1.0241454 | 9.157629428 | -3.6289812 | 0.0003844 | 0.0012182 | -0.63075 |
| PTK6 | -1.0239333 | 5.785363434 | -5.6245422 | 8.31E-08 | 6.56E-07 | 7.422251 |
| CD48 | -1.0221152 | 9.39658566 | -4.2290457 | 3.97E-05 | 0.0001609 | 1.506577 |
| IFNGR1 | -1.0221138 | 10.7261505 | -8.5591815 | 9.73E-15 | 4.41E-13 | 22.98278 |
| JDP2 | -1.0220016 | 8.468442958 | -7.2374968 | 1.90E-11 | 3.76E-10 | 15.56957 |
| CLEC5A | -1.0213318 | 7.453269693 | -2.3726579 | 0.0188703 | 0.0379794 | -4.17586 |
| HLA-DRA | -1.0210324 | 13.59263471 | -3.9266167 | 0.0001286 | 0.0004602 | 0.395933 |
| GPR141 | -1.0209267 | 6.677861263 | -4.7795245 | 4.01E-06 | 2.10E-05 | 3.692496 |
| P4HA2 | -1.0201924 | 2.531430394 | -3.6759816 | 0.0003247 | 0.0010506 | -0.47312 |
| SPI1 | -1.0195146 | 11.43789991 | -9.7098263 | 9.43E-18 | 9.80E-16 | 29.78845 |
| PLA2G4C | -1.0178429 | 3.871525614 | -3.5270752 | 0.000551 | 0.0016757 | -0.96663 |
| VIM | -1.0171504 | 14.74279875 | -5.9405278 | 1.77E-08 | 1.64E-07 | 8.92118 |
| KIF16B | -1.0170393 | 7.875560607 | -3.9579833 | 0.0001142 | 0.0004133 | 0.508017 |
| CLEC4F | -1.0170017 | 1.462376619 | -4.0672603 | 7.51E-05 | 0.0002844 | 0.904165 |
| ID2 | -1.0168764 | 9.333896211 | -4.8911308 | 2.46E-06 | 1.35E-05 | 4.16018 |
| PVRL4 | -1.0151178 | 2.589996438 | -3.9393654 | 0.0001226 | 0.0004409 | 0.441401 |
| GAS2L1 | -1.0140619 | 6.851616905 | -4.4387091 | 1.70E-05 | 7.58E-05 | 2.314822 |
| SORBS3 | -1.0133808 | 8.485986679 | -4.5076225 | 1.27E-05 | 5.90E-05 | 2.587114 |
| RAPH1 | -1.0121142 | 6.858372946 | -4.974728 | 1.70E-06 | 9.69E-06 | 4.515668 |
| DRD5 | -1.0111191 | 2.186634709 | -4.9811 | 1.65E-06 | 9.46E-06 | 4.542944 |
| TAS2R60 | -1.0110371 | 4.026525543 | -3.9073808 | 0.0001383 | 0.0004916 | 0.327558 |
| SRGAP2 | -1.0101793 | 10.53530609 | -9.2684587 | 1.39E-16 | 1.01E-14 | 27.14834 |
| FTH1 | -1.0092876 | 13.11679508 | -8.6679904 | 5.11E-15 | 2.47E-13 | 23.61472 |
| SLC35E4 | -1.0091423 | 5.029247096 | -5.3633511 | 2.87E-07 | 2.00E-06 | 6.224367 |
| ALDH3B1 | -1.0082593 | 10.33085694 | -5.9717482 | 1.51E-08 | 1.43E-07 | 9.072109 |
| TMEM149 | -1.0081718 | 7.939140921 | -7.8717223 | 5.35E-13 | 1.53E-11 | 19.06083 |
| GNS | -1.0075059 | 11.89604496 | -8.3353893 | 3.63E-14 | 1.42E-12 | 21.69214 |
| MX2 | -1.0066586 | 9.622734076 | -5.7874895 | 3.76E-08 | 3.23E-07 | 8.188621 |
| NFKB2 | -1.0064504 | 8.862709472 | -6.9329311 | 1.01E-10 | 1.65E-09 | 13.94402 |
| TNFRSF9 | -1.0059757 | 2.960384372 | -4.6172542 | 8.04E-06 | 3.92E-05 | 3.02691 |
| KCNK6 | -1.0047419 | 7.795656344 | -5.0539107 | 1.19E-06 | 7.08E-06 | 4.856404 |
| BLNK | -1.001631 | 6.693464185 | -2.8297054 | 0.0052676 | 0.012384 | -3.04032 |
| CPPED1 | -1.0004052 | 10.52279687 | -8.2923215 | 4.68E-14 | 1.75E-12 | 21.44523 |
| PCDHB12 | 1.0018604 | 1.468793189 | 4.1304157 | 5.87E-05 | 0.0002283 | 1.137085 |
| ZNF880 | 1.0031945 | 7.060873616 | 6.76590247 | 2.48E-10 | 3.69E-09 | 13.06813 |
| PRKCQ | 1.0036226 | 8.862939103 | 6.03308983 | 1.11E-08 | 1.08E-07 | 9.370089 |
| PTK7 | 1.0041173 | 9.100850093 | 3.83100122 | 0.0001841 | 0.0006328 | 0.058793 |
| ZNF829 | 1.0041728 | 5.549388647 | 7.14577729 | 3.16E-11 | 5.88E-10 | 15.07631 |
| RTKN | 1.0046085 | 6.589885696 | 4.04486481 | 8.19E-05 | 0.0003069 | 0.822265 |
| SCN2A | 1.0047405 | 2.803569243 | 2.36565069 | 0.0192183 | 0.0386128 | -4.19183 |
| KIAA1274 | 1.0055115 | 6.908167813 | 3.34747985 | 0.0010205 | 0.0028911 | -1.53862 |
| CMBL | 1.0077673 | 8.108289373 | 4.38372701 | 2.13E-05 | 9.22E-05 | 2.09991 |
| ZNF253 | 1.0082399 | 8.551401685 | 7.79846763 | 8.13E-13 | 2.22E-11 | 18.65085 |
| KDM5B | 1.0092568 | 11.8362783 | 10.2918691 | 2.60E-19 | 4.35E-17 | 33.31319 |
| ZBTB46 | 1.0099074 | 8.081429033 | 5.34975773 | 3.06E-07 | 2.12E-06 | 6.163086 |
| OR2L1P | 1.0102336 | 3.714749897 | 3.64676378 | 0.0003607 | 0.0011521 | -0.57131 |
| ELFN2 | 1.0115206 | 2.902279514 | 3.15350125 | 0.001933 | 0.0051118 | -2.12723 |
| DNAJC12 | 1.01208 | 5.192923165 | 2.65189982 | 0.0088246 | 0.0195003 | -3.50378 |
| C9orf9 | 1.012681 | 6.313285255 | 5.88942819 | 2.28E-08 | 2.05E-07 | 8.675227 |
| ZC3HAV1L | 1.0152801 | 7.498833741 | 8.25542617 | 5.80E-14 | 2.10E-12 | 21.23409 |
| PIRT | 1.0162087 | 1.674561349 | 5.53274517 | 1.29E-07 | 9.76E-07 | 6.996884 |
| ADAMTS14 | 1.0167444 | 6.167952153 | 2.88555081 | 0.0044568 | 0.0107011 | -2.88915 |
| IRGM | 1.017826 | 5.730708978 | 4.75942153 | 4.37E-06 | 2.27E-05 | 3.609106 |
| FBXL13 | 1.0192439 | 5.035628019 | 5.25701034 | 4.71E-07 | 3.11E-06 | 5.747842 |
| FLG | 1.0199848 | 4.185625046 | 2.89757113 | 0.004298 | 0.0103694 | -2.85626 |
| PLXNB1 | 1.0208519 | 7.545929741 | 3.64596527 | 0.0003617 | 0.0011542 | -0.57399 |
| ALMS1P | 1.0223212 | 2.248845067 | 5.94757667 | 1.70E-08 | 1.59E-07 | 8.955213 |
| SHF | 1.0249642 | 6.074080345 | 5.48935241 | 1.59E-07 | 1.17E-06 | 6.797444 |
| CEL | 1.0250216 | 6.159970128 | 4.89825094 | 2.38E-06 | 1.32E-05 | 4.190286 |
| ZNF571 | 1.0251085 | 7.209387709 | 7.50379836 | 4.32E-12 | 9.81E-11 | 17.01897 |
| LRRC70 | 1.0259224 | 7.789027849 | 5.94563818 | 1.72E-08 | 1.60E-07 | 8.945851 |
| ZNF221 | 1.02664 | 6.732269087 | 6.61559297 | 5.51E-10 | 7.56E-09 | 12.28987 |
| ZNF660 | 1.026778 | 7.474074542 | 7.55827207 | 3.18E-12 | 7.50E-11 | 17.31849 |
| PDE9A | 1.0273807 | 3.206764876 | 2.80380779 | 0.0056875 | 0.013233 | -3.10951 |
| KLF17 | 1.0280674 | 1.8131368 | 5.28982311 | 4.05E-07 | 2.71E-06 | 5.894174 |
| ATP7B | 1.0297238 | 7.307986624 | 3.4446082 | 0.0007334 | 0.0021627 | -1.23246 |
| SMAD1 | 1.0330687 | 8.620606537 | 3.84506763 | 0.0001747 | 0.0006034 | 0.107961 |
| VWCE | 1.0339055 | 6.498192453 | 4.01244135 | 9.27E-05 | 0.0003431 | 0.704342 |
| C10orf68 | 1.0344079 | 3.949222381 | 6.37559522 | 1.93E-09 | 2.31E-08 | 11.06776 |
| GPLD1 | 1.0349731 | 5.098658418 | 5.70656249 | 5.59E-08 | 4.60E-07 | 7.806221 |
| LRP5 | 1.0350872 | 9.577483778 | 5.21817582 | 5.64E-07 | 3.66E-06 | 5.575477 |
| ZNF578 | 1.0351498 | 4.231588847 | 4.00437415 | 9.57E-05 | 0.000353 | 0.675121 |
| TAS2R4 | 1.0368439 | 4.61824039 | 6.2951462 | 2.93E-09 | 3.33E-08 | 10.66397 |
| FAM69B | 1.037213 | 9.210314803 | 4.79776349 | 3.70E-06 | 1.96E-05 | 3.76838 |
| TXNRD3IT1 | 1.0385821 | 6.422459982 | 4.99246289 | 1.57E-06 | 9.04E-06 | 4.591647 |
| C6orf170 | 1.0409871 | 7.39041344 | 7.7456485 | 1.10E-12 | 2.89E-11 | 18.35628 |
| APBA1 | 1.0450324 | 4.285035766 | 2.46060653 | 0.0149526 | 0.0309664 | -3.97163 |
| CXorf22 | 1.0459089 | 1.133549763 | 4.81940267 | 3.37E-06 | 1.80E-05 | 3.858689 |
| TRIM6 | 1.0466102 | 6.719572327 | 4.95817655 | 1.83E-06 | 1.04E-05 | 4.444937 |
| ABCA11P | 1.0468494 | 5.486413899 | 4.11542314 | 6.22E-05 | 0.0002408 | 1.081531 |
| HIP1R | 1.0470529 | 7.676261658 | 5.71483003 | 5.37E-08 | 4.43E-07 | 7.845126 |
| ZNF300 | 1.0471241 | 6.509792803 | 4.5975311 | 8.74E-06 | 4.23E-05 | 2.947195 |
| CRISPLD1 | 1.0502073 | 6.805379253 | 2.95314208 | 0.0036288 | 0.0089474 | -2.70264 |
| NTNG2 | 1.0511978 | 8.002019563 | 3.5181719 | 0.0005684 | 0.0017215 | -0.99559 |
| ZNF876P | 1.0531075 | 3.905702554 | 4.71584918 | 5.28E-06 | 2.69E-05 | 3.429262 |
| STON1 | 1.0535066 | 5.988537184 | 3.96145384 | 0.0001127 | 0.0004088 | 0.520463 |
| KIAA1549 | 1.0540539 | 8.618390404 | 4.45479835 | 1.59E-05 | 7.16E-05 | 2.378104 |
| ZNF626 | 1.0543031 | 6.037349818 | 2.79517308 | 0.0058341 | 0.0135334 | -3.13246 |
| NANOS3 | 1.0544211 | 2.697721953 | 4.84791755 | 2.97E-06 | 1.61E-05 | 3.978151 |
| GPSM1 | 1.0545657 | 9.392962879 | 5.19712008 | 6.21E-07 | 3.98E-06 | 5.482397 |
| TIE1 | 1.0555282 | 7.513221813 | 3.5420907 | 0.0005228 | 0.0015994 | -0.91765 |
| LOC168474 | 1.0567367 | 4.556220125 | 5.42953566 | 2.11E-07 | 1.51E-06 | 6.524257 |
| DOK4 | 1.0581555 | 6.456424771 | 4.61340463 | 8.17E-06 | 3.98E-05 | 3.011331 |
| NPM2 | 1.0584046 | 5.444991278 | 3.54468166 | 0.000518 | 0.0015867 | -0.90918 |
| SAMD13 | 1.0619908 | 5.203113678 | 6.90571103 | 1.17E-10 | 1.88E-09 | 13.80051 |
| ZNF385D | 1.0620185 | 1.767261087 | 2.9847775 | 0.003292 | 0.0082055 | -2.61402 |
| RAB39B | 1.0635587 | 5.934973412 | 6.52783603 | 8.74E-10 | 1.14E-08 | 11.84001 |
| IGF1R | 1.0638078 | 9.440600398 | 4.09192087 | 6.82E-05 | 0.0002612 | 0.994771 |
| KIAA1147 | 1.0641046 | 11.23164151 | 9.81284106 | 5.01E-18 | 5.60E-16 | 30.40903 |
| ZNF471 | 1.0644124 | 6.928324402 | 5.25674742 | 4.72E-07 | 3.12E-06 | 5.746672 |
| DYX1C1 | 1.0655382 | 4.650042972 | 4.46368613 | 1.53E-05 | 6.95E-05 | 2.413138 |
| XKR3 | 1.0677945 | 2.814984271 | 3.81201698 | 0.0001975 | 0.0006727 | -0.00733 |
| C2orf52 | 1.0680892 | 3.193873772 | 6.43431521 | 1.43E-09 | 1.77E-08 | 11.36437 |
| OR2L8 | 1.0684947 | 2.251154835 | 4.44850374 | 1.63E-05 | 7.32E-05 | 2.353326 |
| AKR1C3 | 1.0688906 | 8.504112611 | 4.19986204 | 4.46E-05 | 0.0001784 | 1.396524 |
| WDR31 | 1.0710964 | 5.899443981 | 7.54881596 | 3.35E-12 | 7.87E-11 | 17.26643 |
| ZNF85 | 1.0738957 | 7.988041966 | 7.71385583 | 1.32E-12 | 3.38E-11 | 18.17939 |
| GUCY1B3 | 1.0743781 | 8.195725251 | 3.34351467 | 0.0010342 | 0.0029253 | -1.55095 |
| ZNF323 | 1.0750062 | 6.734587811 | 7.06107976 | 5.02E-11 | 8.94E-10 | 14.62363 |
| HPGD | 1.0763537 | 5.883924567 | 2.43859062 | 0.0158587 | 0.0326139 | -4.0234 |
| PCDHB14 | 1.0769936 | 3.156409061 | 3.56782509 | 0.0004775 | 0.0014772 | -0.83329 |
| ZNF732 | 1.0770669 | 2.678975392 | 5.13229691 | 8.35E-07 | 5.17E-06 | 5.197505 |
| C14orf33 | 1.0782308 | 5.773913934 | 7.81746532 | 7.29E-13 | 2.02E-11 | 18.75702 |
| PPM1H | 1.0800829 | 8.552920308 | 5.36418144 | 2.86E-07 | 1.99E-06 | 6.228114 |
| TERT | 1.0828006 | 4.137134451 | 3.60376399 | 0.0004205 | 0.001319 | -0.71462 |
| ZDHHC15 | 1.0842491 | 4.626007149 | 3.82758394 | 0.0001864 | 0.0006399 | 0.046871 |
| ACSM3 | 1.0848992 | 7.819092226 | 4.56296747 | 1.01E-05 | 4.83E-05 | 2.808126 |
| TSPAN10 | 1.0859954 | 2.325339575 | 5.31868794 | 3.54E-07 | 2.41E-06 | 6.023422 |
| ABCG1 | 1.0875245 | 7.802767341 | 4.5533385 | 1.05E-05 | 5.00E-05 | 2.769526 |
| C14orf128 | 1.0896393 | 3.184546955 | 3.89930851 | 0.0001426 | 0.000505 | 0.298947 |
| OR13D1 | 1.089837 | 3.06270843 | 6.27446956 | 3.26E-09 | 3.67E-08 | 10.56068 |
| PRRT4 | 1.0905177 | 5.993172792 | 3.10986008 | 0.002223 | 0.0057854 | -2.25541 |
| ZNF177 | 1.0909774 | 6.948293916 | 8.12784262 | 1.22E-13 | 4.10E-12 | 20.50683 |
| TFDP2 | 1.0911065 | 9.477707065 | 8.73162956 | 3.50E-15 | 1.74E-13 | 23.98559 |
| LOC100101938 | 1.0911991 | 1.977559444 | 2.4958755 | 0.0135969 | 0.0285044 | -3.88779 |
| DLC1 | 1.0913422 | 8.395795915 | 3.50859243 | 0.0005877 | 0.0017727 | -1.02667 |
| BTBD11 | 1.0940947 | 8.297744317 | 3.9180902 | 0.0001328 | 0.0004742 | 0.365592 |
| AGBL3 | 1.0946681 | 2.854608623 | 6.69723639 | 3.57E-10 | 5.10E-09 | 12.7114 |
| BCAT1 | 1.0949729 | 10.21788045 | 3.65281998 | 0.0003529 | 0.0011286 | -0.55101 |
| VASH2 | 1.0959462 | 4.948480766 | 5.46948915 | 1.74E-07 | 1.28E-06 | 6.706502 |
| ZNF827 | 1.0969263 | 8.135221438 | 3.72530921 | 0.0002716 | 0.0008968 | -0.30585 |
| ZNF665 | 1.0982905 | 5.861487884 | 7.18180228 | 2.59E-11 | 4.91E-10 | 15.26967 |
| SLC18A2 | 1.0995965 | 5.296597254 | 2.75522588 | 0.0065582 | 0.0149712 | -3.23777 |
| DLGAP2 | 1.0997309 | 3.01981121 | 2.50802523 | 0.0131559 | 0.0276908 | -3.85866 |
| TEX9 | 1.1005337 | 5.298591186 | 5.86628052 | 2.55E-08 | 2.28E-07 | 8.564255 |
| CD96 | 1.1013963 | 10.40685206 | 3.27882335 | 0.0012835 | 0.0035331 | -1.75045 |
| SCN11A | 1.1023031 | 3.628528812 | 5.28125838 | 4.21E-07 | 2.81E-06 | 5.855918 |
| OR2L3 | 1.1038675 | 2.556656153 | 4.31537707 | 2.81E-05 | 0.0001181 | 1.835664 |
| RPGRIP1L | 1.1045429 | 6.779974852 | 8.76572988 | 2.86E-15 | 1.49E-13 | 24.1847 |
| AEBP1 | 1.1046761 | 8.207888028 | 4.20555043 | 4.36E-05 | 0.0001748 | 1.417928 |
| TMEM17 | 1.1048777 | 5.72742894 | 7.96102027 | 3.20E-13 | 9.62E-12 | 19.56281 |
| CACNA1C | 1.105069 | 4.940041647 | 5.21027991 | 5.84E-07 | 3.78E-06 | 5.540541 |
| PMS2L2 | 1.1062777 | 7.68038931 | 9.25653471 | 1.49E-16 | 1.08E-14 | 27.07748 |
| ZNF781 | 1.1079166 | 4.728686795 | 5.27401426 | 4.36E-07 | 2.89E-06 | 5.823594 |
| TBC1D16 | 1.1093634 | 6.251218102 | 4.25946262 | 3.52E-05 | 0.0001446 | 1.621924 |
| SPDYE8P | 1.1093881 | 8.726867763 | 9.22933904 | 1.76E-16 | 1.24E-14 | 26.91595 |
| OR2AK2 | 1.1120475 | 3.138106964 | 4.26692832 | 3.41E-05 | 0.0001408 | 1.650335 |
| PCDHGA1 | 1.112983 | 2.818818405 | 4.72835201 | 5.00E-06 | 2.56E-05 | 3.480741 |
| COL14A1 | 1.1134993 | 4.299736876 | 3.09496064 | 0.0023309 | 0.0060333 | -2.29881 |
| DNMT3B | 1.1147363 | 9.114182801 | 5.21810729 | 5.64E-07 | 3.66E-06 | 5.575174 |
| SPNS2 | 1.1156262 | 9.617409499 | 4.66322997 | 6.61E-06 | 3.30E-05 | 3.213733 |
| NBEA | 1.1162148 | 5.718481002 | 3.41984625 | 0.0007984 | 0.0023335 | -1.31123 |
| RNF212 | 1.1189598 | 2.392737022 | 3.27736972 | 0.0012897 | 0.0035478 | -1.7549 |
| DPY19L2P1 | 1.1219125 | 3.128286034 | 3.99331647 | 9.98E-05 | 0.0003668 | 0.635146 |
| TAS2R5 | 1.1221117 | 5.459179245 | 7.21004609 | 2.22E-11 | 4.28E-10 | 15.42161 |
| SLC10A5 | 1.1228808 | 3.791152418 | 4.89551001 | 2.41E-06 | 1.33E-05 | 4.178693 |
| NEDD4 | 1.1243758 | 9.494372987 | 6.72357492 | 3.10E-10 | 4.51E-09 | 12.84799 |
| STOX1 | 1.1287759 | 4.850265911 | 5.39299953 | 2.50E-07 | 1.77E-06 | 6.358396 |
| ZNF439 | 1.136997 | 9.218649066 | 8.05144465 | 1.90E-13 | 6.03E-12 | 20.07351 |
| KLHL31 | 1.1373609 | 3.790292595 | 5.12040236 | 8.81E-07 | 5.43E-06 | 5.145505 |
| NRXN2 | 1.1383402 | 9.353724641 | 3.76315666 | 0.0002365 | 0.0007924 | -0.17625 |
| GAS2 | 1.140142 | 3.642586939 | 5.31073823 | 3.67E-07 | 2.49E-06 | 5.987777 |
| ZCCHC18 | 1.1409381 | 4.531593136 | 6.00022019 | 1.31E-08 | 1.26E-07 | 9.210182 |
| ZNF486 | 1.1414379 | 6.377044166 | 4.69634733 | 5.74E-06 | 2.90E-05 | 3.34917 |
| AMOTL1 | 1.1425645 | 8.185777581 | 3.59791806 | 0.0004293 | 0.0013433 | -0.73399 |
| GATA2 | 1.1434704 | 10.10319352 | 3.84103944 | 0.0001773 | 0.0006117 | 0.093866 |
| MAP7 | 1.1439205 | 9.247579011 | 4.9363755 | 2.01E-06 | 1.13E-05 | 4.352033 |
| LOC728723 | 1.145822 | 4.549014814 | 6.57605645 | 6.78E-10 | 9.10E-09 | 12.08678 |
| CLGN | 1.1472235 | 4.656274199 | 2.87128239 | 0.0046523 | 0.0111032 | -2.92802 |
| ADAMTS3 | 1.1541824 | 3.880287539 | 2.5690875 | 0.0111272 | 0.0239077 | -3.71026 |
| MYO1B | 1.1557763 | 5.56937912 | 3.70159357 | 0.000296 | 0.0009695 | -0.38651 |
| APP | 1.156668 | 9.736950419 | 2.40044432 | 0.0175444 | 0.0356692 | -4.11208 |
| CPA3 | 1.1569043 | 9.518624099 | 2.57474032 | 0.0109543 | 0.0235895 | -3.69636 |
| TRO | 1.1579258 | 5.348408422 | 2.65854352 | 0.00866 | 0.0191756 | -3.48695 |
| TRAT1 | 1.1596594 | 6.60339785 | 4.06202796 | 7.66E-05 | 0.0002894 | 0.884997 |
| OR52H1 | 1.1605064 | 3.286833717 | 6.54435683 | 8.01E-10 | 1.05E-08 | 11.92444 |
| EGFL7 | 1.1606271 | 9.280592006 | 3.66489027 | 0.0003379 | 0.0010867 | -0.51047 |
| GPC2 | 1.1614554 | 5.254394637 | 6.26778161 | 3.37E-09 | 3.78E-08 | 10.52731 |
| PCDHB9 | 1.1617087 | 1.980321495 | 4.178336 | 4.86E-05 | 0.000193 | 1.315738 |
| PCDHGB8P | 1.1633926 | 3.941625894 | 4.98971472 | 1.59E-06 | 9.13E-06 | 4.57986 |
| C6orf225 | 1.1643335 | 4.878579172 | 6.10797594 | 7.62E-09 | 7.72E-08 | 9.736415 |
| CD59 | 1.1668838 | 9.424636993 | 4.46893228 | 1.50E-05 | 6.82E-05 | 2.433842 |
| PRSSL1 | 1.1677432 | 10.71558831 | 4.53879249 | 1.12E-05 | 5.27E-05 | 2.711333 |
| RAG1 | 1.1677564 | 5.161890452 | 4.93000281 | 2.07E-06 | 1.16E-05 | 4.324933 |
| EGF | 1.1679523 | 4.058065386 | 3.04656678 | 0.0027157 | 0.0069188 | -2.4385 |
| LOC149134 | 1.1704341 | 7.354904158 | 5.8468611 | 2.81E-08 | 2.48E-07 | 8.471372 |
| OR2L2 | 1.1707732 | 3.783155595 | 4.01309313 | 9.25E-05 | 0.0003424 | 0.706704 |
| CAPN14 | 1.1729604 | 4.089135284 | 5.11502788 | 9.03E-07 | 5.54E-06 | 5.122037 |
| DNAH6 | 1.1731708 | 3.72190119 | 4.18800378 | 4.68E-05 | 0.0001862 | 1.35198 |
| C21orf128 | 1.1744476 | 4.781137296 | 3.90671965 | 0.0001387 | 0.0004927 | 0.325213 |
| JUB | 1.1747341 | 4.188704982 | 5.52105363 | 1.36E-07 | 1.03E-06 | 6.943044 |
| SLAIN1 | 1.1751404 | 6.494025205 | 3.85164025 | 0.0001705 | 0.0005909 | 0.130987 |
| ZBED3 | 1.1754221 | 6.590700232 | 6.56653871 | 7.13E-10 | 9.53E-09 | 12.03799 |
| CYP4F2 | 1.1794903 | 3.448184409 | 2.95927912 | 0.0035611 | 0.0087952 | -2.68551 |
| ALDH5A1 | 1.179568 | 9.610227674 | 9.37055646 | 7.48E-17 | 5.86E-15 | 27.75615 |
| TRIM74 | 1.1831633 | 3.32053349 | 5.52827116 | 1.32E-07 | 9.95E-07 | 6.976272 |
| MESTIT1 | 1.1858539 | 4.083528774 | 4.38804621 | 2.09E-05 | 9.09E-05 | 2.116717 |
| ZNF709 | 1.185934 | 7.324721264 | 7.5602216 | 3.14E-12 | 7.43E-11 | 17.32923 |
| ELOVL2 | 1.1878421 | 2.33983102 | 3.88496328 | 0.0001505 | 0.0005298 | 0.248223 |
| ZBED2 | 1.1905421 | 5.849730765 | 3.96146046 | 0.0001127 | 0.0004088 | 0.520487 |
| GPR125 | 1.1931236 | 9.400582109 | 7.0436135 | 5.52E-11 | 9.65E-10 | 14.53063 |
| SRGAP3 | 1.1934768 | 7.112957681 | 3.81147283 | 0.0001979 | 0.0006737 | -0.00922 |
| PCDHB8 | 1.1962818 | 1.591586608 | 4.92730073 | 2.10E-06 | 1.17E-05 | 4.31345 |
| CCND2 | 1.1967941 | 12.02769561 | 6.56563438 | 7.17E-10 | 9.57E-09 | 12.03335 |
| ZNF114 | 1.19854 | 4.768699443 | 5.51432971 | 1.41E-07 | 1.06E-06 | 6.912114 |
| ANGPT1 | 1.1989127 | 9.905688975 | 3.79472288 | 0.0002106 | 0.0007138 | -0.06733 |
| MAN1A1 | 1.1993474 | 11.10566552 | 5.22453264 | 5.47E-07 | 3.57E-06 | 5.60363 |
| VANGL2 | 1.2033321 | 5.153617991 | 3.36091509 | 0.0009754 | 0.0027786 | -1.49672 |
| TSPAN5 | 1.2035572 | 5.132900034 | 4.28424989 | 3.18E-05 | 0.0001321 | 1.716405 |
| KHDRBS3 | 1.2042285 | 3.495401977 | 3.29014904 | 0.0012362 | 0.0034202 | -1.71577 |
| RHOBTB1 | 1.2044951 | 7.554154373 | 4.2977737 | 3.01E-05 | 0.0001258 | 1.768136 |
| NKD2 | 1.2053472 | 2.415130222 | 3.15845599 | 0.0019024 | 0.0050391 | -2.11258 |
| BMP3 | 1.2054226 | 2.339105191 | 2.9220561 | 0.0039903 | 0.0097176 | -2.78889 |
| OR2L13 | 1.2072402 | 3.833829454 | 3.94181347 | 0.0001214 | 0.0004374 | 0.450146 |
| C15orf33 | 1.2084914 | 3.456717597 | 7.4948269 | 4.54E-12 | 1.02E-10 | 16.96973 |
| FAM180B | 1.2088576 | 4.5843606 | 5.37533796 | 2.72E-07 | 1.90E-06 | 6.278494 |
| ZNF391 | 1.2104287 | 4.661972182 | 4.34735101 | 2.47E-05 | 0.0001049 | 1.958873 |
| AIF1L | 1.2118552 | 7.300400186 | 3.06002336 | 0.0026032 | 0.0066636 | -2.39985 |
| KCNA6 | 1.2146435 | 4.829435849 | 3.8380434 | 0.0001793 | 0.0006179 | 0.08339 |
| GNRHR | 1.2152502 | 5.532814823 | 6.36737122 | 2.02E-09 | 2.40E-08 | 11.02634 |
| SALL4 | 1.2155244 | 3.74533914 | 3.03296408 | 0.0028339 | 0.0071829 | -2.47742 |
| ZNF681 | 1.2192398 | 7.583196205 | 6.12867745 | 6.86E-09 | 7.05E-08 | 9.83817 |
| LRP12 | 1.2208614 | 7.938093407 | 6.27576941 | 3.24E-09 | 3.65E-08 | 10.56716 |
| ZNF737 | 1.2211288 | 9.024279672 | 6.61571458 | 5.50E-10 | 7.56E-09 | 12.29049 |
| MYOZ3 | 1.2247891 | 6.313008112 | 6.23703657 | 3.95E-09 | 4.32E-08 | 10.3742 |
| ARHGAP5 | 1.2254645 | 9.167344626 | 4.09306666 | 6.79E-05 | 0.0002602 | 0.998991 |
| DSCR6 | 1.2258029 | 1.477744696 | 4.36779219 | 2.27E-05 | 9.77E-05 | 2.038015 |
| LOC342346 | 1.2282424 | 4.183020756 | 6.33025929 | 2.45E-09 | 2.85E-08 | 10.83984 |
| LPHN3 | 1.2325978 | 3.170998639 | 2.73244033 | 0.0070069 | 0.015897 | -3.29723 |
| SPTLC3 | 1.233662 | 5.747500929 | 3.55490133 | 0.0004998 | 0.0015374 | -0.87572 |
| SUCNR1 | 1.2348593 | 7.864274095 | 4.11130178 | 6.32E-05 | 0.0002442 | 1.066288 |
| CABLES1 | 1.2389819 | 7.207063852 | 4.77049124 | 4.17E-06 | 2.18E-05 | 3.654992 |
| DEPDC6 | 1.246354 | 9.057854949 | 7.8051073 | 7.83E-13 | 2.15E-11 | 18.68794 |
| CD69 | 1.2478004 | 10.89057004 | 5.59013052 | 9.81E-08 | 7.64E-07 | 7.262249 |
| DYRK3 | 1.2535245 | 7.489859965 | 6.04241835 | 1.06E-08 | 1.03E-07 | 9.41557 |
| C19orf51 | 1.2561244 | 3.441874843 | 3.62683278 | 0.0003873 | 0.0012256 | -0.63792 |
| PABPC4L | 1.2635319 | 1.926264913 | 3.16360016 | 0.0018711 | 0.0049618 | -2.09735 |
| SLC14A1 | 1.2643627 | 6.849283572 | 4.40133857 | 1.98E-05 | 8.66E-05 | 2.168523 |
| MAST4 | 1.2655292 | 9.566275403 | 6.93763623 | 9.82E-11 | 1.61E-09 | 13.96886 |
| SOX4 | 1.2694871 | 11.87483565 | 7.35711118 | 9.80E-12 | 2.07E-10 | 16.21748 |
| DPY19L2 | 1.2730886 | 7.960977077 | 3.5621348 | 0.0004872 | 0.0015027 | -0.85199 |
| RGS9BP | 1.273479 | 3.287452443 | 5.82039572 | 3.20E-08 | 2.79E-07 | 8.345105 |
| C21orf34 | 1.2735896 | 3.188168091 | 3.55322908 | 0.0005027 | 0.0015456 | -0.8812 |
| GPR87 | 1.2760676 | 2.754049083 | 4.23110659 | 3.94E-05 | 0.0001598 | 1.514372 |
| DMC1 | 1.2767996 | 4.0358984 | 5.08571485 | 1.03E-06 | 6.22E-06 | 4.994349 |
| TBC1D19 | 1.2771589 | 5.844704918 | 9.54662573 | 2.56E-17 | 2.33E-15 | 28.80854 |
| SLC38A4 | 1.282164 | 3.747169537 | 5.59797432 | 9.44E-08 | 7.38E-07 | 7.298663 |
| ESYT3 | 1.2834583 | 3.605448824 | 5.52013933 | 1.37E-07 | 1.03E-06 | 6.938837 |
| ZNF154 | 1.2839119 | 8.210471308 | 6.58427365 | 6.50E-10 | 8.77E-09 | 12.12893 |
| C1QTNF4 | 1.2908313 | 7.998716897 | 3.26771128 | 0.0013316 | 0.0036488 | -1.78438 |
| LOC286467 | 1.2940995 | 7.661551959 | 8.25303292 | 5.88E-14 | 2.12E-12 | 21.22041 |
| PCBP3 | 1.2946389 | 4.068170815 | 3.25866 | 0.001372 | 0.0037478 | -1.81194 |
| RSPH1 | 1.2954797 | 2.392801996 | 5.53380264 | 1.28E-07 | 9.72E-07 | 7.001757 |
| PSD2 | 1.2993778 | 2.956287706 | 4.97209882 | 1.72E-06 | 9.78E-06 | 4.504421 |
| JUP | 1.3000542 | 10.91711522 | 4.90073713 | 2.36E-06 | 1.30E-05 | 4.200806 |
| TOX | 1.304282 | 8.518368316 | 6.17248039 | 5.49E-09 | 5.82E-08 | 10.05417 |
| GPR174 | 1.304695 | 7.46055345 | 6.11732189 | 7.27E-09 | 7.38E-08 | 9.782327 |
| TMEFF1 | 1.3056464 | 3.892644609 | 6.67030255 | 4.12E-10 | 5.83E-09 | 12.57202 |
| WT1 | 1.3074609 | 6.877006766 | 3.61540192 | 0.0004034 | 0.0012707 | -0.67598 |
| SYCP2L | 1.3096295 | 2.875126773 | 3.06316528 | 0.0025775 | 0.0066053 | -2.39081 |
| MAP1A | 1.3105846 | 9.805726751 | 5.01056515 | 1.45E-06 | 8.38E-06 | 4.6694 |
| MEX3A | 1.3107009 | 5.76759565 | 3.82708986 | 0.0001868 | 0.0006407 | 0.045148 |
| TMPRSS11D | 1.310936 | 3.147847469 | 6.42027254 | 1.53E-09 | 1.88E-08 | 11.29329 |
| SMO | 1.3172768 | 5.747250393 | 4.37322302 | 2.22E-05 | 9.58E-05 | 2.05909 |
| GRIK5 | 1.318701 | 8.854408465 | 4.1495262 | 5.44E-05 | 0.0002132 | 1.208134 |
| FLJ13197 | 1.32472 | 7.157079312 | 6.30425326 | 2.80E-09 | 3.20E-08 | 10.70953 |
| MRC2 | 1.3273478 | 8.460688715 | 4.32538146 | 2.70E-05 | 0.0001137 | 1.874139 |
| GPR12 | 1.3314935 | 3.295068147 | 3.06424652 | 0.0025688 | 0.0065849 | -2.38769 |
| LOC26102 | 1.3367548 | 3.570429748 | 5.54599937 | 1.21E-07 | 9.23E-07 | 7.058012 |
| NAALADL2 | 1.3470214 | 2.98233508 | 4.3527335 | 2.41E-05 | 0.0001029 | 1.979684 |
| MMRN1 | 1.350477 | 9.337811778 | 3.94154455 | 0.0001216 | 0.0004377 | 0.449185 |
| PCDHB13 | 1.3526895 | 3.016069666 | 4.2165563 | 4.17E-05 | 0.0001679 | 1.459405 |
| C20orf203 | 1.3559181 | 5.149872643 | 4.93379649 | 2.04E-06 | 1.14E-05 | 4.341063 |
| LOC100188949 | 1.3602275 | 5.856082414 | 6.7042905 | 3.44E-10 | 4.96E-09 | 12.74795 |
| NOG | 1.364181 | 5.998170074 | 3.9648273 | 0.0001113 | 0.000404 | 0.53257 |
| PLCH1 | 1.3648572 | 5.118376779 | 4.01231856 | 9.28E-05 | 0.0003432 | 0.703896 |
| TFPI | 1.3667769 | 9.227616562 | 5.31526432 | 3.60E-07 | 2.44E-06 | 6.008067 |
| EPCAM | 1.3705817 | 3.700101935 | 3.20166972 | 0.0016538 | 0.0044365 | -1.98394 |
| C21orf62 | 1.375764 | 5.158498211 | 7.7574861 | 1.03E-12 | 2.71E-11 | 18.42222 |
| AVPR1B | 1.37675 | 2.963201535 | 5.23565618 | 5.20E-07 | 3.41E-06 | 5.652952 |
| LHX6 | 1.3851326 | 3.852722836 | 3.2727194 | 0.0013098 | 0.0035955 | -1.7691 |
| CDH4 | 1.3864668 | 2.752370465 | 3.09128633 | 0.0023583 | 0.0060963 | -2.30949 |
| MSI2 | 1.4014232 | 9.898191192 | 6.54342208 | 8.05E-10 | 1.06E-08 | 11.91966 |
| SV2A | 1.4040635 | 9.135107303 | 5.97628773 | 1.48E-08 | 1.40E-07 | 9.094095 |
| KIAA0748 | 1.4052031 | 9.699502759 | 7.52032763 | 3.93E-12 | 9.06E-11 | 17.10975 |
| MLLT11 | 1.4057563 | 7.36309207 | 8.24695397 | 6.10E-14 | 2.17E-12 | 21.18566 |
| ZNF704 | 1.4089776 | 4.758396136 | 3.51151375 | 0.0005818 | 0.0017577 | -1.0172 |
| C1orf186 | 1.4127237 | 8.674307365 | 4.97412326 | 1.70E-06 | 9.71E-06 | 4.513081 |
| MYT1 | 1.4127951 | 2.461734091 | 4.42352876 | 1.81E-05 | 8.01E-05 | 2.255278 |
| ZSCAN23 | 1.4235166 | 4.243386094 | 3.88285122 | 0.0001517 | 0.000533 | 0.240767 |
| MEST | 1.4273836 | 9.799421807 | 4.42575597 | 1.79E-05 | 7.95E-05 | 2.264004 |
| GATM | 1.433507 | 6.39608348 | 3.72996376 | 0.000267 | 0.0008831 | -0.28997 |
| OPALIN | 1.4615734 | 2.829688053 | 3.03005344 | 0.0028598 | 0.0072417 | -2.48572 |
| UGT2B28 | 1.4617507 | 2.707693038 | 4.40605243 | 1.94E-05 | 8.51E-05 | 2.186924 |
| CSRP2 | 1.465769 | 3.469415358 | 4.61243071 | 8.20E-06 | 3.99E-05 | 3.007391 |
| LOC100101266 | 1.4733722 | 4.034476527 | 7.03309918 | 5.85E-11 | 1.01E-09 | 14.47469 |
| TSPAN13 | 1.4772654 | 7.68831666 | 5.92371276 | 1.92E-08 | 1.76E-07 | 8.840098 |
| CEP70 | 1.4792443 | 8.166518467 | 7.8696994 | 5.41E-13 | 1.54E-11 | 19.04949 |
| LOC646851 | 1.492064 | 5.498236118 | 7.05642351 | 5.15E-11 | 9.13E-10 | 14.59883 |
| CD200 | 1.494621 | 7.928719584 | 3.60763813 | 0.0004147 | 0.0013035 | -0.70177 |
| KLHL23 | 1.4950202 | 7.230342272 | 8.04231916 | 2.00E-13 | 6.31E-12 | 20.02187 |
| THSD7A | 1.49849 | 3.552428637 | 2.73936238 | 0.0068677 | 0.0155988 | -3.27921 |
| WASF1 | 1.5052516 | 8.921337916 | 5.37396886 | 2.73E-07 | 1.91E-06 | 6.272308 |
| MDFI | 1.5111878 | 5.813729029 | 3.47958475 | 0.00065 | 0.0019369 | -1.12037 |
| TAS2R3 | 1.5115451 | 3.964650872 | 7.33952981 | 1.08E-11 | 2.25E-10 | 16.12192 |
| PALM | 1.5175914 | 6.797650822 | 4.34067879 | 2.53E-05 | 0.0001074 | 1.933103 |
| COL24A1 | 1.5211059 | 9.519583176 | 6.83436215 | 1.71E-10 | 2.66E-09 | 13.42575 |
| ZNF610 | 1.5235577 | 6.272106101 | 8.29811678 | 4.52E-14 | 1.70E-12 | 21.47842 |
| ERG | 1.5247084 | 10.48836732 | 7.04398999 | 5.51E-11 | 9.64E-10 | 14.53263 |
| KIT | 1.5259826 | 11.53758425 | 6.10682645 | 7.66E-09 | 7.76E-08 | 9.730771 |
| MED12L | 1.5311662 | 7.643093295 | 4.37322697 | 2.22E-05 | 9.58E-05 | 2.059105 |
| C1orf150 | 1.5444283 | 7.76149183 | 4.01163223 | 9.30E-05 | 0.000344 | 0.701409 |
| MPL | 1.5446842 | 8.224077159 | 4.78527016 | 3.91E-06 | 2.06E-05 | 3.716378 |
| MEX3B | 1.545144 | 8.46473014 | 7.79040496 | 8.51E-13 | 2.32E-11 | 18.60583 |
| MYO5C | 1.5474952 | 7.95782708 | 4.38790156 | 2.09E-05 | 9.09E-05 | 2.116154 |
| DKFZP434L187 | 1.5673512 | 4.287893631 | 4.69737417 | 5.71E-06 | 2.89E-05 | 3.353381 |
| C9orf93 | 1.578953 | 7.523124517 | 9.53250874 | 2.79E-17 | 2.49E-15 | 28.72397 |
| KIAA1211 | 1.5874853 | 7.531333784 | 4.76199716 | 4.32E-06 | 2.25E-05 | 3.619775 |
| PROM1 | 1.5891334 | 9.3266634 | 2.82208675 | 0.0053881 | 0.0126397 | -3.06073 |
| SORCS1 | 1.5933561 | 3.875146643 | 4.08291252 | 7.06E-05 | 0.000269 | 0.961622 |
| ITM2A | 1.5951205 | 9.872874548 | 5.21532312 | 5.71E-07 | 3.71E-06 | 5.562851 |
| HHIP | 1.6069599 | 2.351559701 | 5.72041743 | 5.22E-08 | 4.34E-07 | 7.871441 |
| MACC1 | 1.6074178 | 7.151515171 | 4.35469933 | 2.39E-05 | 0.0001022 | 1.98729 |
| STK32B | 1.612484 | 6.990761459 | 4.6045099 | 8.48E-06 | 4.12E-05 | 2.975371 |
| SHD | 1.6176789 | 3.710321601 | 3.33112252 | 0.0010782 | 0.0030332 | -1.58943 |
| TMIGD2 | 1.6300137 | 5.625309013 | 4.0768309 | 7.23E-05 | 0.000275 | 0.939276 |
| ATP9A | 1.6476225 | 5.950283524 | 4.89110525 | 2.46E-06 | 1.35E-05 | 4.160072 |
| TSPAN7 | 1.6520699 | 5.446624068 | 3.1501079 | 0.0019542 | 0.0051604 | -2.13726 |
| C9orf43 | 1.6523607 | 5.764929137 | 6.85116101 | 1.57E-10 | 2.45E-09 | 13.5138 |
| ITGA9 | 1.660948 | 8.234522684 | 4.16113106 | 5.20E-05 | 0.0002047 | 1.251406 |
| CYTL1 | 1.6681634 | 8.314330802 | 4.74614442 | 4.63E-06 | 2.40E-05 | 3.554174 |
| ZNF667 | 1.6747709 | 6.499592245 | 4.62979953 | 7.62E-06 | 3.74E-05 | 3.077749 |
| ZNF793 | 1.6805336 | 6.580737216 | 5.72052941 | 5.22E-08 | 4.34E-07 | 7.871968 |
| UGT2B11 | 1.6820344 | 4.115947398 | 3.49684983 | 0.0006123 | 0.0018398 | -1.06468 |
| EMID1 | 1.716884 | 8.485632269 | 6.02463506 | 1.16E-08 | 1.12E-07 | 9.328906 |
| DYTN | 1.7585207 | 4.231034423 | 5.78553324 | 3.80E-08 | 3.26E-07 | 8.179336 |
| ABCB1 | 1.7894813 | 6.858749166 | 5.07868006 | 1.06E-06 | 6.41E-06 | 4.963783 |
| IKZF2 | 1.8061467 | 8.737233306 | 5.40822511 | 2.33E-07 | 1.66E-06 | 6.427422 |
| ZBTB8A | 1.8113652 | 8.436538869 | 8.82785312 | 1.97E-15 | 1.09E-13 | 24.54809 |
| GUCY1A3 | 1.8360199 | 10.74914986 | 5.99713331 | 1.33E-08 | 1.27E-07 | 9.195192 |
| C5orf23 | 1.8438859 | 9.065735278 | 4.15677999 | 5.29E-05 | 0.0002079 | 1.23517 |
| NPR3 | 1.8541838 | 8.118885327 | 3.97543866 | 0.0001069 | 0.0003893 | 0.570707 |
| C19orf77 | 1.8568522 | 9.132546526 | 7.1478066 | 3.12E-11 | 5.83E-10 | 15.08719 |
| CBX2 | 1.8754308 | 8.810488438 | 7.8567821 | 5.82E-13 | 1.65E-11 | 18.97708 |
| ZNF608 | 1.8993328 | 7.10512414 | 5.35697321 | 2.96E-07 | 2.06E-06 | 6.195601 |
| IGLL1 | 1.8997511 | 8.122536837 | 4.06932947 | 7.45E-05 | 0.0002824 | 0.91175 |
| SVOPL | 1.900003 | 3.241431205 | 6.70065055 | 3.51E-10 | 5.03E-09 | 12.72909 |
| SHANK3 | 1.9110635 | 9.324161702 | 4.80065846 | 3.66E-06 | 1.93E-05 | 3.780445 |
| POU4F1 | 1.9314958 | 3.292838403 | 3.82542414 | 0.0001879 | 0.0006436 | 0.03934 |
| B4GALT6 | 1.9365161 | 9.808114397 | 10.3173161 | 2.22E-19 | 3.80E-17 | 33.46822 |
| UMODL1 | 1.9435202 | 6.205201618 | 4.46733982 | 1.51E-05 | 6.86E-05 | 2.427555 |
| ADAM22 | 1.9572584 | 4.659342909 | 7.78791671 | 8.63E-13 | 2.34E-11 | 18.59194 |
| TRH | 1.9851308 | 5.571329165 | 3.18679288 | 0.0017358 | 0.0046387 | -2.0284 |
| MYCN | 1.9993621 | 7.428547937 | 4.94973563 | 1.90E-06 | 1.07E-05 | 4.408931 |
| MYO18B | 2.0130241 | 2.465647203 | 3.86330063 | 0.0001632 | 0.0005692 | 0.171915 |
| VPREB1 | 2.0812084 | 3.061805 | 5.38628576 | 2.58E-07 | 1.82E-06 | 6.328002 |
| BAALC | 2.0947041 | 8.21855022 | 4.18333568 | 4.76E-05 | 0.0001894 | 1.334472 |
| GATSL2 | 2.1009961 | 2.872579104 | 6.07983025 | 8.78E-09 | 8.70E-08 | 9.598406 |
| DNTT | 2.1322915 | 6.108616546 | 2.92184583 | 0.0039929 | 0.0097224 | -2.78948 |
| UGT3A2 | 2.2065553 | 3.990435091 | 5.6062733 | 9.07E-08 | 7.10E-07 | 7.337226 |
| CD34 | 2.4660285 | 10.38021225 | 4.12799632 | 5.92E-05 | 0.0002303 | 1.12811 |
| HPGDS | 2.5351099 | 6.547677223 | 5.49179298 | 1.57E-07 | 1.16E-06 | 6.808633 |
| ARPP21 | 2.6937459 | 2.759706457 | 5.61744786 | 8.60E-08 | 6.76E-07 | 7.389212 |
| FLJ22536 | 2.7165897 | 4.701058529 | 6.98445116 | 7.62E-11 | 1.29E-09 | 14.21647 |
