# Supplementary material for: CDK6 Is a Potential Prognostic Biomarker in Acute Myeloid Leukemia
Source: Front Genet. 2021 Feb 1;11:600227. doi: 10.3389/fgene.2020.600227 (PMC7882723; doi:10.3389/fgene.2020.600227)
Supplement: Supplementary file 3 [file Table_2.docx]

|  | logFC | AveExpr |  | t | P.Value | adj.P.Val | B |
| --- | --- | --- | --- | --- | --- | --- | --- |
| hsa-mir-6503 | -2.30544 | 4.0486 |  | -8.6315 | 6.75E-15 | 1.27E-11 | 21.48659 |
| hsa-mir-582 | -2.16186 | 9.735595 |  | -8.20133 | 8.39E-14 | 7.89E-11 | 18.98475 |
| hsa-mir-9-2 | -1.62871 | 6.876345 |  | -4.0659 | 7.58E-05 | 0.002547 | -1.22988 |
| hsa-mir-9-3 | -1.55897 | 6.871324 |  | -3.86226 | 0.000165 | 0.004767 | -1.96752 |
| hsa-mir-9-1 | -1.5187 | 6.86915 |  | -3.78378 | 0.00022 | 0.006078 | -2.24351 |
| hsa-mir-3150b | -1.39218 | 3.340002 |  | -6.93368 | 1.04E-10 | 2.78E-08 | 11.94032 |
| hsa-mir-4662a | -1.23065 | 3.555875 |  | -4.50015 | 1.32E-05 | 0.00059 | 0.443689 |
| hsa-mir-501 | -1.22568 | 5.858378 |  | -6.20515 | 4.75E-09 | 6.38E-07 | 8.173146 |
| hsa-mir-10a | -1.20196 | 14.6904 |  | -2.49103 | 0.013788 | 0.157188 | -6.07283 |
| hsa-mir-500a | -1.17312 | 8.832115 |  | -6.57948 | 6.84E-10 | 1.43E-07 | 10.07926 |
| hsa-mir-708 | -1.15138 | 4.05471 |  | -3.94891 | 0.000119 | 0.003723 | -1.65745 |
| hsa-mir-3614 | -1.14906 | 2.785953 |  | -5.75258 | 4.54E-08 | 4.49E-06 | 5.960044 |
| hsa-mir-362 | -1.14646 | 4.197709 |  | -7.12506 | 3.66E-11 | 1.38E-08 | 12.96732 |
| hsa-mir-660 | -1.04582 | 5.808897 |  | -6.51926 | 9.38E-10 | 1.76E-07 | 9.768282 |
| hsa-mir-187 | -1.02867 | 1.548909 |  | -4.68951 | 5.96E-06 | 0.00032 | 1.214457 |
| hsa-mir-6718 | -1.01981 | 4.63756 |  | -2.85554 | 0.004884 | 0.069599 | -5.13557 |
| hsa-mir-126 | 1.038538 | 13.3535 |  | 3.500161 | 0.000607 | 0.014638 | -3.20107 |
| hsa-mir-99b | 1.054729 | 12.69946 |  | 3.706403 | 0.000292 | 0.007734 | -2.51095 |
| hsa-mir-125b-2 | 1.057886 | 6.302199 |  | 3.569073 | 0.000477 | 0.012123 | -2.9742 |
| hsa-mir-1266 | 1.078458 | 2.132562 |  | 4.463658 | 1.54E-05 | 0.000659 | 0.297957 |
| hsa-mir-551a | 1.08863 | 4.609426 |  | 4.984485 | 1.64E-06 | 0.000113 | 2.462179 |
| hsa-mir-125b-1 | 1.098028 | 6.164965 |  | 3.593313 | 0.000438 | 0.01128 | -2.89351 |
| hsa-mir-1468 | 1.115343 | 2.608141 |  | 5.879341 | 2.44E-08 | 2.55E-06 | 6.569274 |
| hsa-mir-146a | 1.183765 | 11.08251 |  | 5.564794 | 1.12E-07 | 9.61E-06 | 5.073541 |
| hsa-mir-181b-1 | 1.245933 | 10.48792 |  | 6.06951 | 9.44E-09 | 1.18E-06 | 7.498916 |
| hsa-mir-181b-2 | 1.249293 | 9.96141 |  | 6.264768 | 3.50E-09 | 5.07E-07 | 8.472305 |
| hsa-mir-181a-2 | 1.27033 | 13.34314 |  | 6.845911 | 1.66E-10 | 3.90E-08 | 11.47423 |
| hsa-mir-125a | 1.271041 | 7.823559 |  | 4.527874 | 1.18E-05 | 0.000555 | 0.555029 |
| hsa-mir-181a-1 | 1.292999 | 13.37994 |  | 5.986936 | 1.43E-08 | 1.58E-06 | 7.092982 |
| hsa-mir-130a | 1.307934 | 7.76507 |  | 4.356817 | 2.39E-05 | 0.000939 | -0.12344 |
| hsa-mir-335 | 1.468827 | 7.72626 |  | 4.790543 | 3.85E-06 | 0.000234 | 1.635482 |
| hsa-mir-181c | 1.510893 | 8.155631 |  | 7.492446 | 4.79E-12 | 2.25E-09 | 14.97768 |
| hsa-mir-181d | 1.63189 | 5.542294 |  | 8.063575 | 1.86E-13 | 1.17E-10 | 18.19408 |

Supplementary Table2: DEGS of microRNA between  *CDK6*^high^  and *CDK6*^low^ in non-APL AML patients
